# Multi-start heuristic approaches for one-to-one pickup and delivery problems with shortest-path transport along real-life paths

**DOI:** 10.1371/journal.pone.0227702

**Published:** 2020-02-06

**Authors:** Xin Qi, Zhuo Fu, Jian Xiong, Weixiong Zha

**Affiliations:** 1 School of Traffic and Transportation Engineering, Central South University, Changsha, Hunan, China; 2 School of Transportation and Logistics, East China Jiao Tong University, Nanchang, Jiangxi, China; National Taiwan University of Science and Technology, TAIWAN

## Abstract

The One-to-one Pickup and Delivery Problem with Shortest-path Transport along Real-life Paths (OPDPSTRP) is presented in this paper. It is a variation of the One-to-one Pickup and Delivery Problem (OPDP), which is common in daily life, such as the Passenger Train Operation Plans (PTOP) and partial Taxi-sharing Problem. Unlike the classical OPDP, in the OPDPSTRP, (1) each demand must be transported along the shortest path according to passengers/shippers requirements, and (2) each vehicle should travel along a real-life path. First, six route structure rules are proposed for the OPDPSTRP, and a kind of Mixed-Integer Programming (MIP) models is formulated for it. Second, A Variable Neighborhood Descent (VND), a Variable Neighborhood Research (VNS), a Multi-Start VND (MS_VND) and a Multi-Start VNS (MS_VNS) with five neighborhood operators has been developed to solve the problem. Finally, The Gurobi solver, the VND, the VNS, the MS_VND and the MS_VNS have been compared with each other by 84 random instances partitioned in small size connected graphs, medium size connected graphs and large size connected graphs. From the test results we found that solutions generated by these approaches are often comparable with those found by the Gurobi solver, and the solutions found by these approaches are better than the solutions found by the Gurobi solver when solving instances with larger numbers of demands. In almost all instances, the MS_VND significantly outperforms the VND and the VNS in terms of solution quality, and outperforms the MS_VNS both in terms of solution quality and CPU time. In the instances with large numbers of demands, the MS_VND is still able to generate good feasible solutions in a reasonable CPU time, which is of vital practical significance for real-life instances.

## 1 Introduction

Nowadays, a China high-speed rail network has been formed, it is an urgent problem to design the Passenger Train Operation Plans (PTOP) based on networks, which is different from the general PTOP based on lines. Generally, there are two features in the PTOP that (1) passengers should be transported through the shortest path and (2) trains cannot visit any station more than once. So the PTOP based on networks can be refined as: There are several pickup-delivery demands (pd-pairs) and vehicles in a real-life connected graph. Each pd-pair chosen must be transported through the shortest path from the pickup point to the delivery point according to passenger/shipper requirements. Each vehicle starts at a given location and ends at the final delivery point of the pd-pairs transported by the vehicle, and cannot visit (stop at or pass through) any point more than once, namely each vehicle should travel along a real-life path. Constraints, such as vehicle load capacities, vehicle travel distances, and vehicle stops, need to be considered. This problem can be addressed by introducing a set of maximum-income routes to be driven by a fleet of vehicles to serve a group of known pd-pairs. Referred to as One-to-one Pickup and Delivery Problems with Shortest-path Transport along Real-life Paths (OPDPSTRP), this can be classed under One-to-one Pickup and Delivery Problem (OPDP). Since each pd-pair must be transported along the shortest path and vehicle stops need to be considered, the OPDPSTRP will be studied based on connected graphs, which shouldn’t be abstracted into complete graphs. The OPDPSTRP can also be applied in some transportation problems with these two features of the PTOP based on real-life connected graph, such as partial Taxi-sharing Problem. To the best of our knowledge, the OPDPSTRP has rarely been studied in the literature. So the model of the OPDPSTRP will be studied and efficient algorithms will be proposed for it in this paper.

Section 2 presents related studies, while Section 3 studies the relationships between pd-pairs and presents the model for the OPDPSTRP. Section 4 presents a Variable Neighborhood Descent (VND), a Variable Neighborhood Research (VNS), a Multi-Start VND (MS_VND) and a Multi-Start VNS (MS_VNS) based on 5 new neighborhoods for the OPDPSTRP. Section 5 proposes a set of random instances and analyses the efficiency of the Gurobi solver, the VND, the VNS, the MS_VND and the MS_VNS for the OPDPSTRP. Finally, conclusions and future work are presented in Section 6.

## 2 Literature review

The OPDPSTRP belongs to the General Pickup and Delivery Problem (GPDP), which is an NP-hard problem.

### 2.1 GPDP

Many scholars have carried out research on the GPDP over the past few years, in response to numerous kinds of GPDP being applied in real-life, such as GPDP with Time Windows, Dynamic, Stochastic, Unpaired/Paired, Single/Multi vehicle, Single/Multi depot and Single/Multi commodity.

**Fig 1:**
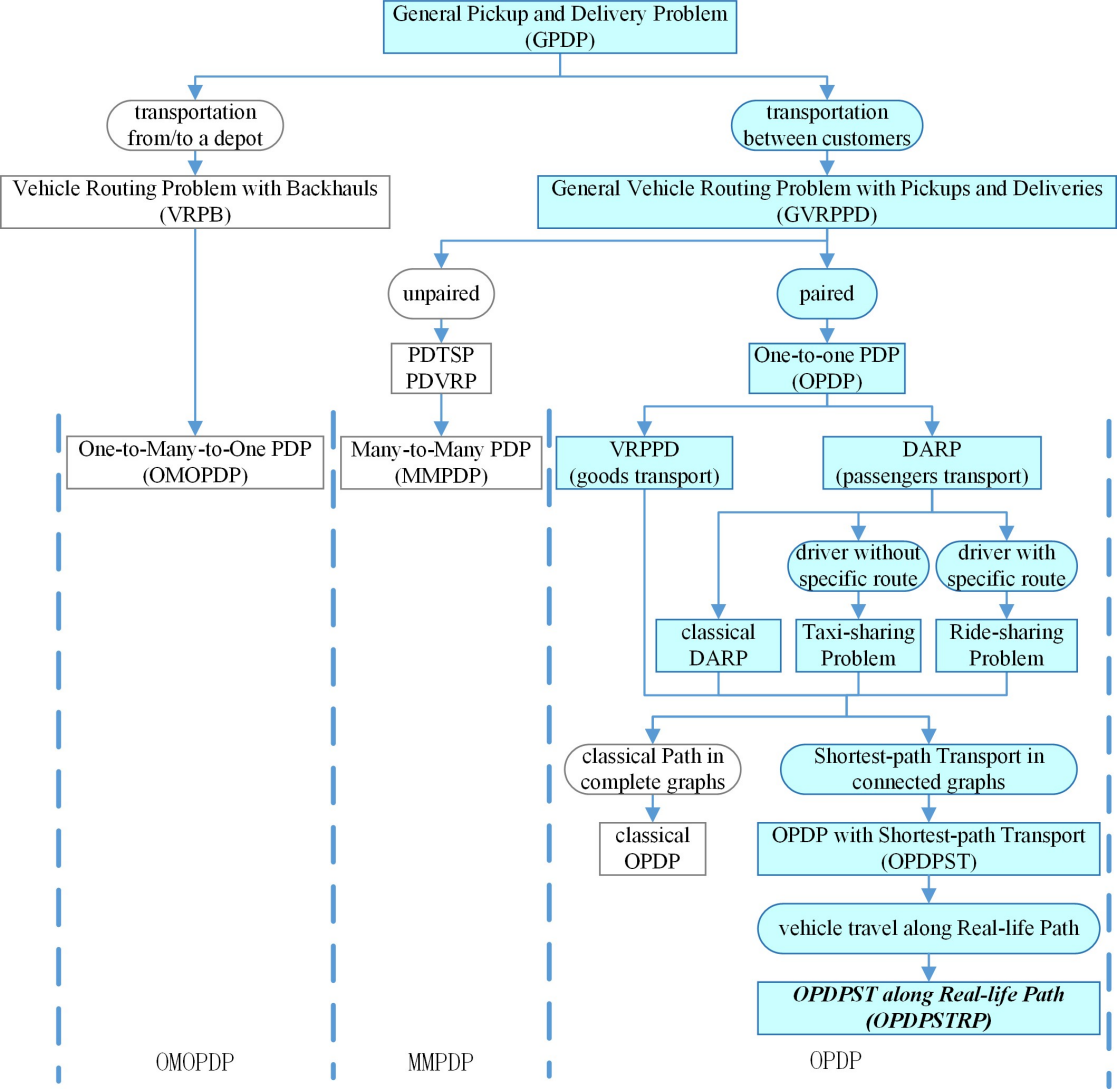
Classification of GPDPs.

Parragh et al. [1], [2] reviewed current GPDP research and divided the studies into two categories, as illustrated in Fig 1. The first category comprises the transportation of goods from a depot to line-haul customers and from back-haul customers to the depot, denoted as the Vehicle Routing Problem with Back-hauls (VRPB). The research on VRPB was reviewed by Koç and Laporte [3]. The second category considers all problems that occur where goods are transported between pickup and delivery locations, denoted as the General Vehicle Routing Problem with Pickups and Deliveries (GVRPPD). In this paper, the problem being studied belongs to the latter category.

The GVRPPD can be further divided into two sub-classes: unpaired and paired. The first sub-class refers to situations where pickup and delivery locations are unpaired and each unit picked up can be used to fulfill the demands of any delivery customer, such as Many-to-many PDP (MMPDP, Rieck et al. [4]). The second GVRPPD sub-class is known as the One-to-one Pickup and Delivery Problem (OPDP), such as the Vehicle Routing Problem with Pickups-Deliveries (VRPPD) and the Dial-A-Ride Problem (DARP). Both types of OPDP consider transportation requests, each associated with an origin and a destination, resulting in paired pickup and delivery points (e.g. Agatz et al. [5], Chen et al. [6] and Ho et al. [7]). The VRPPD addresses goods transportation, while the DARP deals with passenger transportation (Berbeglia et al. [8]). Santos et al. [9] listed differences between the Taxi-sharing Problem and the Ride-sharing Problem, which are two kinds of DARP. They pointed out that the private car owner has a specific trip route in the Ride-sharing Problem, while the taxi driver does not have a specific route in the Taxi-sharing Problem, which is similar to the OPDPSTRP studied in this paper.

Berbeglia et al. [8], [10] divided GPDP into three categories: One-to-Many-to-One PDP (OMOPDP, Gribkovskaia et al. [11]), Many-to-many PDP (MMPDP, Psaraftis [12], [13], Sexton [14], [15] and Camargo et al. [16]) and One-to-one PDP (OPDP). They also listed three kinds of OPDP: the swapping problem, single-commodity PDTSP, and single-commodity Q-delivery, with corresponding solution methods presented in their papers. Recently, research into the OPDP (e.g. Pérez et al. [17], Sahin et al. [18] and Soysal et al. [19]) outnumbered studies into the MMPDP (e.g., Rieck et al. [4]) and OMOPDP (e.g., Zhu et al. [20]).

### 2.2 OPDP, OPDPST and OPDPSTRP

Most classical OPDPs are studied in complete graphs, and pickup points must be visited prior to delivery points (e.g. Fleischmann et al. [21], Cornuéjols et al. [22], Aragão et al. [23], Letchford et al. [24], Li et al. [25], Mahmoudi et al. [26]). The classical OPDP can be easily formulated as a Mixed-Integer Program (MIP), such as those reported in Pérez et al. [17], Sahin et al. [18] and Soysal et al. [19]. In a forthcoming article, we studied a new kind of OPDP, named One-to-one Pickup and Delivery Problems with Shortest-path Transport (OPDPST), in which each pd-pair must be transported along the shortest path, and a new kind of modeling method was proposed for the OPDPST according its new route constructions. The OPDPSTRP is a kind of the OPDPST, in which vehicles should travel along real-life paths in connected graphs, such as Train-operation Plan in China and partial Taxi-sharing Problem and so on. Unlike the classical OPDP in a complete graph, the OPDPST and the OPDPSTRP studied in this paper is described by connected graphs, since numbers of vehicle stops need to be considered.

Vehicles should travel along paths both in the classical OPDP and the OPDPST and the OPDPSTRP, some research conducted on the route structure of the classical OPDP can provide some reference for the OPDPSTRP, although travelling along paths in a complete graph doesn’t means travelling along paths in the corresponding real-life connected graph. Furuhata et al. [27] listed four kinds of ride-sharing patterns for the Ride-sharing Problem. Letchford et al. [24] pointed out that the cheapest path is not always the quickest path, and a comparison of multiple paths between each two points was necessary. Muelas et al. [28] proposed a method for relocating a pd-pair by considering four cases, and the shortest one was chosen as the optimal routing scheme in each local search move. Lin et al. [29] and Wang et al. [30] studied the Ride-sharing Problem (a kind of OPDP) in real-life networks. However, it is not a necessary requirement to transport pd-pairs through the shortest route in these papers.

Additionally, as in the OPDPSTRP, each vehicle starts at its location (regarded as a depot) and ends at the final delivery point of the contents transported by the vehicle, so it can be considered as a multi-depot (vehicles) problem. Most OPDP research is based on single depot, such as that reviewed by Psaraftis [12], [13] (1983), Desrosiers et al. [31], Lin et al. [29], Li et al. [32], Letchford et al. [24], Urra et al. [33], Muelas et al. [28], Qiu et al. [34] and Li et al. [25]. There is also some OPDP research based on multiple depots (vehicles), which is mainly concerned with the Taxi-sharing Problem and Ride-sharing Problem. For example, there is a starting point and an ending point for each vehicle in Hosni et al. [35], Ma et al. [36], Sawaya et al. [37] and Santos et al. [9], whilst only the starting point is considered for each vehicle in Detti et al. [38].

Generally, there is far more research on classical OPDP, and little research focuses directly on the OPDPSTRP proposed in this paper.

### 2.3 Neighborhood and algorithm for OPDP

Cordeau et al. [39] presented eight kinds of local search moves for OPDP: Couple-exchange, Block-exchange, Relocate-couple, Relocate-block, Multi-relocate, 2-opt-L, Double-bridge and Shake. Ribeiro et al. [40] and Ropke et al. [41] modified three large neighborhood removal heuristics and two large neighborhood insertion heuristics from Shaw P. [42], [43] and Potvin et al. [44] for OPDP. Additionally, studies of Grimault et al. [45] and Ho et al. [7] show that solution feasibility of the OPDP is an important issue to the neighborhoods efficiency for the algorithm.

Rodríguez-Martín et al. [46] solved the OPDPTSP by the Greedy Randomized Adaptive Search Procedures (GRASP) and the VND. Sahin et al. [18] proposed an efficient heuristic that combines the strengths of Tabu Search and Simulated Annealing for the OPDPSD. A Iterated Local Search (ILS) is proposed by Li et al. [47]. Ho et al. [7] classified the solution methods for the DARP (an important category of the OPDP). Factorovich et al. [48] propose a combination of cutting planes to find feasible solutions for the pickup and delivery problem with incompatibility constraints. Additionally, some Local Search (LS) meta-heuristics studied for the PDP can also be used for reference. Adaptive Large Neighborhood Search (ALNS) proposed for the PDP by Ropke et al. [41] and Li et al. [25]. Berbeglia et al. [8], [9] reviewed the algorithms for the static and the dynamic PDP. There are also some exact methods proposed for the OPDP, Pérez et al. [17] solve two mixed integer linear programming models of the OPDPTSP by the Cplex solver. Soysal et al. [19] proposed a mixed integer programming model for the green OPDP, and solved it by the Cplex solver.

Generally, research for the OPDP is mainly based on the heuristic techniques, such as the VNS, the VND, the ILS, the ALNS, the hybrid algorithms and so on, because exact methods are often not the most effective way to solve large OPDP.

## 3 Problem definition and mathematical model

### 3.1 Problem definition

In order to define the proposed the OPDPSTRP in mathematical terms, we specify an connected graph *G* = (*N*,*E*,*P*,*K*), in which *N* = {1,…,*n*} for vertexes, *E* = {1,…,*e*} for edges, *P* = {1,…,*p*} for pd-pairs, and *K* = {1,…,*m*} for vehicles. Each pd-pair *i* with demand *q*_*i*_ yields revenue *π*_*i*_. Each vehicle *k*∈*K* has a maximum capacity *Q*^*k*^ and a fixed cost *vc*^*k*^. The transportation cost per unit length of vehicle *k* is *tc*^*k*^. Each vehicle *k* has a stop cost $sc_{n}^{k}$ at node *n*.

The system also obeys the following assumptions.

1. Each pd-pair attribute is different (Perez et al. [17], Psaraftis et al. [49], Miao et al. [50]) and cannot be split (unlike Mitra et al. [51] and Nowak [52], Xia et al. [53]), and must also be transported through the shortest path according to passengers requirements, with pickup point being visited prior to delivery point.
2. Each vehicle must travel along a real-life path beginning with the first pickup point and end at the last delivery point, namely each point cannot be accessed multiple times by a vehicle, a common practice in the Passenger Train Operation Plans and so on. The model with this constraint always can obtain close solutions to the model without this constraint but with less computing time than it, which is elaborated in another initial manuscript, the major results can be found in S3 Appendix.
3. For each vehicle, the travel distance limit (from the first pickup point to the last delivery point) is *D*, and the limit of the number of stops is *M*.
4. The total cost of each vehicle consists of constant cost, travel cost, and stop cost. In order to maximize income, not all pd-pairs need to be transported.
5. There is only one shortest path between any two nodes in the graph.

By defining the afore-mentioned problem, we hope to identify a suitable scheme to help optimize the benefit.

### 3.2 Constants and variables

The constants and variables used in this paper are listed in Table 1.

**Table 1:** Constants and variables.

Notations	Definitions	Attributions
*q*_*i*_	Demand of pd-pair *i*	Constants
*π*_*i*_	Revenue of pd-pair *i*	Constants
*Q*^*k*^	Capacity of vehicle *k*	Constants
*vc*^*k*^	Fixed cost of vehicle *k*	Constants
*tc*^*k*^	Transportation cost per unit length of vehicle *k*	Constants
$sc_{n}^{k}$	Stop cost of vehicle at node *n*	Constants
*le*_*e*_	Length of edge *e*	Constants
*ld*_*index*_*i*,*e*_	Judgment parameter of whether pd-pair *i* moves via edge *e* or not	Constants
*lc*_*i*,*j*_	Length of connecting section for pd-pair *j* to connect to vehicle/pd-pair *i*, *i*∈*P* for pd-pairs and *i* = {*p*+1} for vehicles.	Constants
*connect_to_judge*_*i*,*j*_	Judgment parameter of whether pd-pair *j* can (or cannot) connect to vehicle/pd-pair *i*, *i*∈*P* for pd-pairs and *i* = {*p*+1} for vehicles.	Constants
*connect_after_judge*_*i*,*j*_	Judgment parameter of whether pd-pair *j* can (or cannot) connect after vehicle/pd-pair *i*, *i*∈*P* for pd-pairs and *i* = {*p*+1} for vehicles.	Constants
*sn_od*_*i*,*n*_	Judgment parameter of whether pd-pair *i* can (or cannot) be picked up/delivered at node *n*	Constants
$x_{i,j}^{k}$	Pd-pair *j* connects to pd-pair *i* in vehicle *k* or not, *i*∈*P* for pd-pairs and *i* = {*p*+1} for vehicles.	Variables
$y_{e}^{k}$	Vehicle *k* travels by way of edge *e* with pd-pairs or not	Variables
$u_{i}^{k}$	Sequence number of pd-pair *i* transported by vehicle *k*	Variables
$sn_{n}^{k}$	Vehicle *k* stopping at node *n* or not	Variables

The values of these notations will be studied in Section 3.4.

### 3.3 Mathematical model

The model of the OPDPSTRP is formulated in this section, and the route structure of the OPDPSTRP will be studied for it in Section 3.4.

$$\max\;\{\sum_{k\in K}\sum_{i\in P}\sum_{j\in P\cup \{p+1\}}\pi_{i}\cdot x_{j,i}^{k}-[\sum_{k\in K}\sum_{j\in P}vc^{k}\cdot x_{p+1,j}^{k}+\sum_{k\in K}tc^{k}\cdot (\sum_{e\in E}le_{e}\cdot y_{e}^{k}+\sum_{i\in P\cup \{p+1\}}\sum_{j\in P}lc_{i,j}\cdot x_{i,j}^{k})+\sum_{k\in K}\sum_{n\in N}sc_{n}^{k}\cdot sn_{n}^{k}]\}$$ (1)

$$S.t.\;x_{i,j}^{k}\leq connect\_to\_judge_{i,j}\;\forall k\in K,i\in P\cup \{p+1\},j\in P$$ (2)

$$x_{i,j}^{k}\leq \sum_{i_{0}\in P\cup \{p+1\}}connect\_after\_judge_{i_{0},i}\cdot x_{i_{0},i}^{k}\;\forall k\in K,i\in P,j\in P$$ (3)

$$\sum_{j\in P}connect\_after\_judge_{i,j}\cdot x_{i,j}^{k}\leq 1\;\forall k\in K,i\in P\cup \{p+1\}$$ (4)

$$x_{i,i}^{k}=0\;\forall k\in K,i\in P$$ (5)

$$u_{i}^{k}-u_{j}^{k}+n\cdot x_{i,j}^{k}\leq n-1\;\forall k\in K,i,j\in P$$ (6)

$$\sum_{k\in K}\sum_{i\in P\cup \{p+1\}}x_{i,j}^{k}\leq 1\;\forall \;j\in P$$ (7)

$$\sum_{i\in P}(ld\_index_{i,e}\cdot q_{i}\cdot \sum_{j\in P\cup \{p+1\}}x_{j,i}^{k})\leq Q^{k}\;\forall k\in K,e\in E$$ (8)

$$sn_{n}^{k}\geq sn\_od_{i,n}\cdot \sum_{j\in P\cup \{p+1\}}x_{j,i}^{k}\;\forall k\in K,i\in P,n\in N$$ (9)

$$\sum_{n\in N}sn_{n}^{k}\leq M\;\forall k\in K$$ (10)

$$ld\_index_{i,e}\cdot \sum_{j\in P\cup \{p+1\}}x_{j,i}^{k}\leq y_{e}^{k}\;\forall k\in K,i\in P,e\in E$$ (11)

$$y_{e}^{k}\leq \sum_{j\in P}x_{p+1,j}^{k}\;\forall k\in K,e\in E$$ (12)

$$\sum_{e\in E}le_{e}\cdot y_{e}^{k}+\sum_{i\in P}\sum_{j\in P}lc_{i,j}\cdot x_{i,j}^{k}\leq D\;\forall k\in K$$ (13)

$$x_{i,j}^{k}\in \{0,1\}\;\forall k\in K,i\in P\cup \{p+1\},j\in P$$ (14)

$$y_{e}^{k}\in \{0,1\}\;\forall k\in K,e\in E$$ (15)

$$u_{i}^{k}\in \{1,2,3,\ldots \}\;\forall k\in K,i\in P$$ (16)

$$sn_{n}^{k}\in \{0,1\}\;\forall k\in K,n\in N$$ (17)

The objective function (1) maximizes the total profit, in which $\sum_{k\in K}\sum_{i\in P}\sum_{j\in P\cup \{p+1\}}\pi_{i}\cdot x_{j,i}^{k}$ is the total income, $\sum_{k\in K}\sum_{j\in P}vc^{k}\cdot x_{p+1,j}^{k}$ is the total fixed cost of using vehicles, $\sum_{k\in K}\sum_{e\in E}tc^{k}\cdot le_{e}\cdot y_{e}^{k}$ is the total travel cost due to carry lengths, $\sum_{k\in K}\sum_{i\in P\cup \{p+1\}}\sum_{j\in P}tc^{k}\cdot lc_{i,j}\cdot x_{i,j}^{k}$ is the total traveling cost due to connection lengths and additional lengths, and $\sum_{k\in K}\sum_{n\in N}sc_{n}^{k}\cdot sn_{n}^{k}$ is the total cost of stop nodes. Constraints (2), (3), (4), (5) and (6) determine the order between pd-pairs/vehicle *i* and *j*. Constraints (7) ensure that each pd-pair *i* is transported no more than once. Constraint (8) ensures that each vehicle is not over-loading. Constraint (9) determines whether the vehicle stops at node *n* or not. Constraint (10) ensures that the number of stops for each route (not including the depot/vehicle) does not exceed *M*. Constraint (11) determines whether edge *e* is traveled along or not. Constraints (12) ensure that each route with pd-pairs is assigned to one vehicle. Constraint (13) ensures that the length of each route (not including the depot/vehicle) is not longer than *D*. Constraints (14), (15), (16) and (17) introduce the decision variables.

### 3.4 Route structure of the OPDPSTRP

The route structure of the OPDPSTRP will be studied for the model.

#### 3.4.1 Feasibility of route structure and connection relationship between two pd-pairs or vehicle/pd-pair

**Definition 1:** In a real-life connected graph, if all pd-pairs are transported through the shortest path in a route *i* starting with the first pickup point, and the route is a path, then it is defined that route *i* is Route-Structure-Feasible (RSF for short).

**Definition 2:** If a RSF route stems from inserting pd-pair *i* into route *j*, then it is defined that pd-pair *i* can be inserted into route *j* according route structure. *pd*_*R*_*rs*_*judge*_*i*,*j*_ is defined as the route structure feasibility judgement parameter of inserting (RSFJPI for short, 0: feasible; 1: unfeasible.) pd-pair *i* into route *j*. [*pd*_*R*_*rs*_*judge*_*i*,*j*_] is defined as the route structure feasibility judgement matrix of inserting (RSFJMI for short).

**Definition 3:** If a RSF route is a combination of pd-pair *i* and pd-pair *j*, then it is defined that pd-pair *i* can be combined with pd-pair *j* according route structure. *pd*_*combine*_*rs*_*judge*_*i*,*j*_ is defined as route structure feasibility judgement parameter of combining (RSFJPC for short, 0: feasible; 1: unfeasible) pd-pair *i* with *j*. [*pd*_*combine*_*rs*_*judge*_*i*,*j*_] is defined as the route structure feasibility judgement matrix of combining (RSFJMC for short). It is assumed that each pd-pair can combine with any vehicle, that is *pd*_*combine*_*rs*_*judge*_*p*+*1*,*j*_ = 1(∀*j* = 1,…,*p*).

**Definition 4:** In a RSF route, if pd-pair *j* can be picked up not prior to vehicle/pd-pair *i*, then it is defined that pd-pair *j* can connect to vehicle/pd-pair *i*. The parameter *connect*_*to*_*judge*_*i*,*j*_ (∀*i* = 1,…,*p*+1;*j* = 1,…*p*) is defined as the judgment parameter for pd-pair *j* connecting to vehicle/pd-pair *i*. It is assumed that each pd-pair can connect to any vehicle, that is *connect*_*to*_*judge*_*p*+*1*,*j*_ = 1(∀*j* = 1,…,*p*). One vehicle cannot connect to another vehicle.

**Definition 5:** If pd-pair *i* can connect to vehicle/pd-pair *i* (namely *connect*_*to*_*judge*_*i*,*j*_ = 1), and pd-pair *j* can be delivered not prior to vehicle/pd-pair *i*, then it is defined that pd-pair *j* can connect after vehicle/pd-pair *i*. The parameter *connect*_*after*_*judge*_*i*,*j*_(∀*i* = 1,…,*p*+1;*j* = 1,…*p*) is defined as the judgment parameter for pd-pair *j* connecting after vehicle/pd-pair *i*. It is assumed that each pd-pair can connect after any vehicle, that is, *connect*_*after*_*judge*_*p*+1,*j* =1_(∀*i* = 1,…,*p*).

**Definition 6:** The nodes traveled by vehicle *k* in the route combined by pd-pair *i* and pd-pair *j* can be classified into ***stop nodes*** (stopped at by vehicle *k*) and ***pass nodes*** (passed but not stopped at by vehicle *k*). *p*_*i*_ and *d*_*i*_ are pickup point and delivery point of pd-pair *i* correspondingly. *sn*_*od*_*i*,*n*_ is defined as the judgment parameter of whether pd-pair *i* picks-up/delivers at node *n*.

**Definition 7:** The sections in the route combined with pd-pair *i* and pd-pair *j* include ***weighting sections*** (sections traveled by a vehicle with pd-pairs), and ***connecting sections*** (sections traveled by a vehicle without pd-pairs). The constants *le*_*e*_ and *lc*_*i*,*j*_ are defined as their lengths.

**Fig 2:**
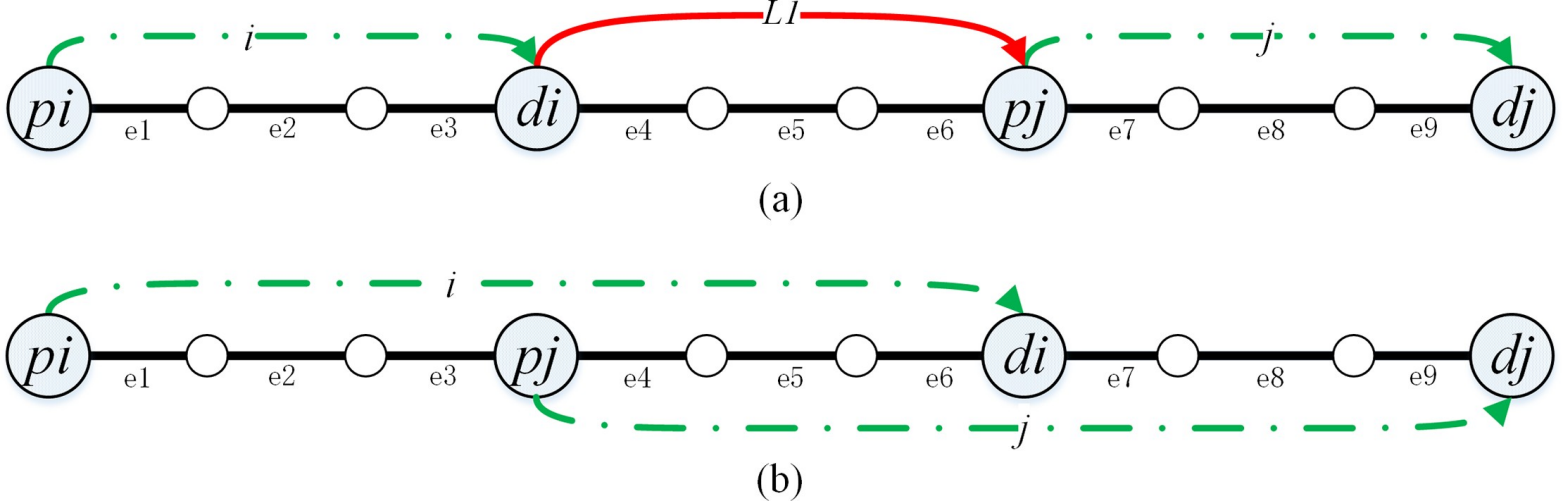
Sections lengths in the route constructed by two pd-pairs.

Table 2 shows the lengths of sections in the paths (beginning with the first pickup point) constructed by pd-pair *i* and pd-pair *j* in Fig 2.

**Table 2:** *le*_*e*_ and *lc*_*i*,*j*_ between two pd-pairs.

Instances	Order	Paths	Length of weighting sections (*le*_*e*_)	Length of connecting sections (*lc*_*i*,*j*_)
**Fig 2(A)**	*j* can connect to *i*	*p*_*i*_*-d*_*i*_*-p*_*j*_*-d*_*j*_	*e*_*1*_+*e*_*2*_+*e*_*3*_+*e*_*7*_+*e*_*8*_+*e*_*9*_	*L1*
	*i* cannot connect to *j*	*-*	*e*_*7*_+*e*_*8*_+*e*_*9*_+*e*_*1*_+*e*_*2*_+*e*_*3*_	∞
**Fig 2(B)**	*j* can connect to *i*	*p*_*i*_*-p*_*j*_*-d*_*i*_*-d*_*j*_	*e*_*1*_+*e*_*2*_+*e*_*3*_+*e*_*4*_+*e*_*5*_+*e*_*6*_+*e*_*7*_+*e*_*8*_+*e*_*9*_	0
	*i* cannot connect to *j*	*-*	*e*_*4*_+*e*_*5*_+*e*_*6*_+*e*_*7*_+*e*_*8*_+*e*_*9*_+*e*_*1*_+*e*_*2*_+*e*_*3*_	∞

The values of *connect*_*to*_*judge*_*i*,*j*_, *connect*_*after*_*judge*_*i*,*j*_ and *lc*_*i*,*j*_ for the routes combined with two pd-pairs are listed in S1 Appendix.

#### 3.4.2 Rules of route construction with more than two pd-pairs/vehicles

Since $x_{i,j}^{k}$ is defined as the decision variable of whether pd-pair *j* connects to pd-pair *i* or not, and *connect*_*after*_*judge*_*i*,*j*_ = 1 means pd-pair *j* can connect after pd-pair *i*, so $x_{i,j}^{k}\cdot connect\_after\_judge_{i,j}=1$ means pd-pair *j* connects after pd-pair *i* successfully (**Definition 4** and **Definition 5**). According to the above research, in a route combined with more than two pd-pairs/vehicle traveled by vehicle *k*, the lengths of the weighting sections and connecting sections can be formulated as $\sum_{e\in E}le_{e}\cdot y_{e}^{k}$ and $\sum_{i\in P}\sum_{j\in P}lc_{i,j}\cdot x_{i,j}^{k}$ respectively, and vehicle *k* stopping at node *n* or not can be determined by $sn_{n}^{k}\geq sn\_od_{i,n}\cdot v_{i}^{k}(\forall i\in P,n\in N)$. However, some rules must be complied with to ensure the feasibility of the route.

***Rule 1*:** Pd-pair *j* can connect to vehicle/pd-pair *i* when *connect*_*to*_*judge*_*i*,*j*_ = 1.
***Rule 2*:** Each pd-pair transported by a vehicle must connect to the vehicle, or another pd-pair that connects after the third pd-pair or vehicle only once.
***Rule 3*:** Each vehicle/pd-pair must not be connected after by more than one pd-pair.
***Rule 4*:** Each pd-pair must not connect to itself.
***Rule 5*:** There cannot be circles in any route.
***Rule 6*:** Each pd-pair not being transported should not connect to any pd-pair or vehicle.

*Rule 1*, *Rule 4*, *Rule 5* and *Rule 6* are apparent.

**Fig 3:**
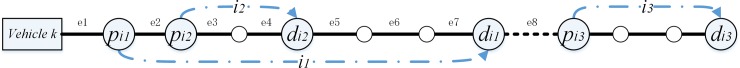
A route structure.

Take Fig 3 for instance. Pd-pairs *i*_1_, *i*_2_ and *i*_3_ are transported by vehicle *k*. The vehicle route must be as follows: pd-pair *i*_1_ connects after vehicle *k* (namely $x_{p+1,i_{1}}^{k}=1$), pd-pair *i*_2_ connects to pd-pair *i*_1_ (namely $x_{i_{1},i_{2}}^{k}=1$), and pd-pair *i*_*3*_ connects after pd-pair *i*_1_ (namely $x_{i_{1},i_{3}}^{k}=1$), according *Rule 2* and *Rule 3*. If $x_{i_{2},i_{3}}^{k}=1$, the length (*e*_5_+*e*_6_+*e*_7_) between points *d*_*i*2_ and *d*_*i*1_ may be double-counted, because pd-pair *i*_2_ does not connect after any pd-pair or vehicle. If $x_{p+1,i_{3}}^{k}=1$, the length (*e*_1_+*e*_2_+*e*_3_+*e*_4+_*e*_5_+*e*_6_+*e*_7_) between point *k* and *d*_*i*1_ may be double-counted, as vehicle *k* has been connected after by pd-pair *i*_1_ already.

All the rules have been considered in Section 3.3 (constraints 2, 3, 4, 5, 6).

#### 3.4.3 A feasible solution for a small instance

**Fig 4:**
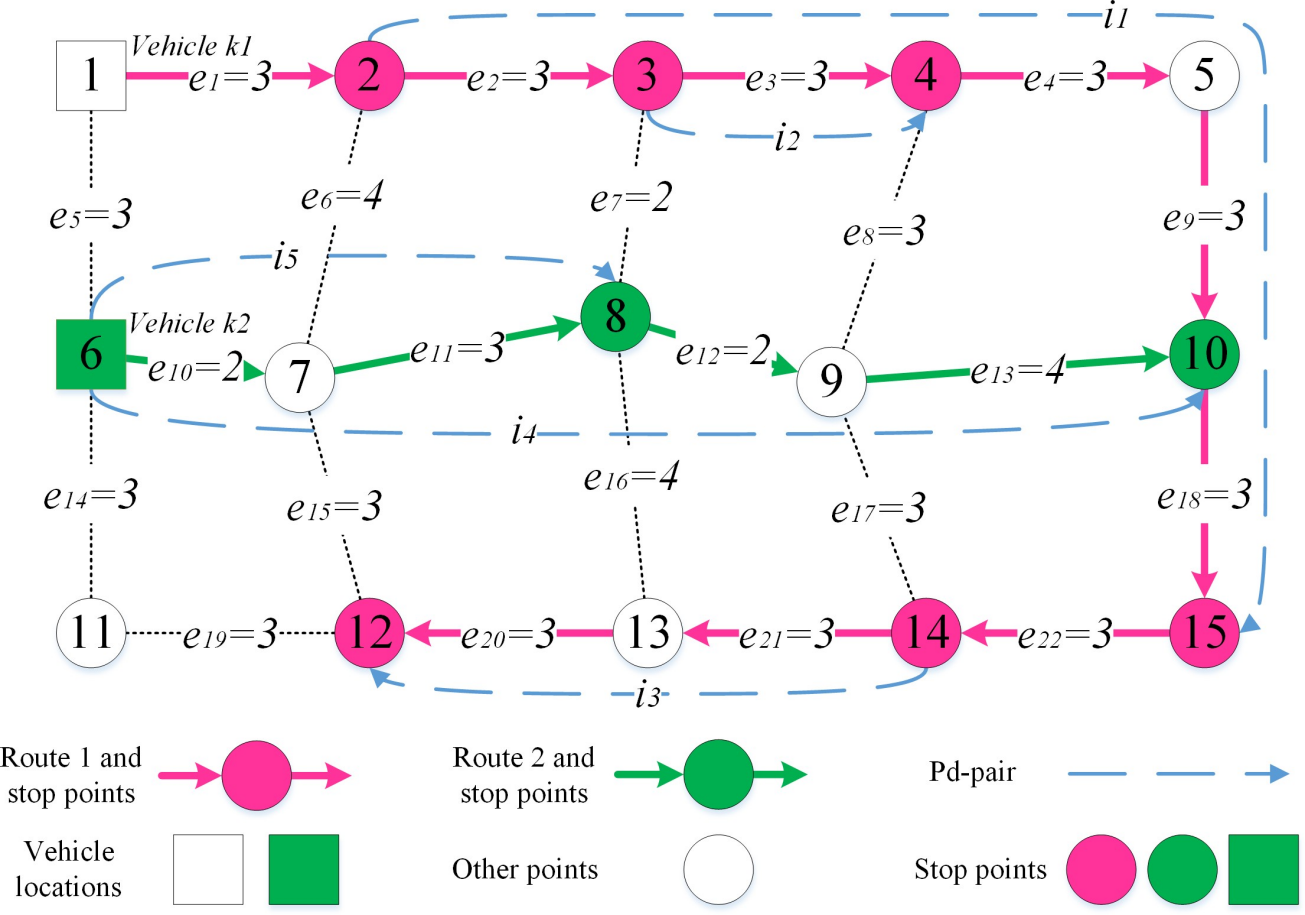
A real-life connected graph with two routes.

Fig 4 is a real-life connected graph, edges lengths are shown in it. In a feasible schedule, six pd-pairs (demand: 1) are transported by two vehicles (capacity: 5, distance limit: 10, stops limit: 6) along two routes. According the above definitions: Route 1 and route 2 are RSF; Pd-pair *i*_5_ can be inserted into route 1 while pd-pair *i*_4_ cannot; Pd-pair *i*_3_ can be combined with *i*_1_ while pd-pair *i*_4_ cannot; Pd-pair *i*_2_ and *i*_3_ can connect to pd-pair *i*_i_, pd-pair *i*_4_ can connect to pd-pair *i*_5_, and pd-pair *i*_5_ can connect to pd-pair *i*_4_; pd-pair *i*_3_ can connect after pd-pair *i*_1_ and *i*_2_, pd-pair *i*_4_ can connect after pd-pair *i*_5_, each pd-pairs can connect after all vehicles; Points 1 and 6 are vehicles locations, points 2, 3, 4, 15, 14, 12, 6, 8 and 10 are stop nodes; *lc*_*k*1,*i*1_ = *le*_*e*1_, *lc*_*i*1,*i*3_ = *le*_*e*22_.

Let *π*_*i*_ = 15, *vc*^*k*^ = 1, *tc*^*k*^ = 1, and $sc_{n}^{k}=1$. The values of the decision variables for the schedule are listed in Table 3 (list non-zero variable only).

**Table 3:** Decision variables for the schedule.

Variables	Route 1	Route 2
$x_{i,j}^{k}$	$x_{k_{1},i_{1}}^{k_{1}}=1$, $x_{i_{1},i_{2}}^{k_{1}}=1$, $x_{i_{1},i_{3}}^{k_{1}}=1$	$x_{k_{2},i_{4}}^{k_{2}}=1$, $x_{i_{4},i_{5}}^{k_{2}}=1$ or $x_{k_{2},i_{5}}^{k_{2}}=1$, $x_{i_{5},i_{4}}^{k_{2}}=1$
$y_{e}^{k}$	$y_{e_{2}}^{k_{1}}=1$, $y_{e_{3}}^{k_{1}}=1$, $y_{e_{4}}^{k_{1}}=1$, $y_{e_{9}}^{k_{1}}=1$, $y_{e_{18}}^{k_{1}}=1$, $y_{e_{21}}^{k_{1}}=1$, $y_{e_{20}}^{k_{1}}=1$	$y_{e_{10}}^{k_{2}}=1$, $y_{e_{11}}^{k_{2}}=1$, $y_{e_{12}}^{k_{2}}=1$, $y_{e_{13}}^{k_{2}}=1$
$sn_{n}^{k}$	$sn_{2}^{k_{1}}=1$, $sn_{3}^{k_{1}}=1$, $sn_{4}^{k_{1}}=1$, $sn_{15}^{k_{1}}=1$, $sn_{14}^{k_{1}}=1$, $sn_{12}^{k_{1}}=1$	$sn_{6}^{k_{2}}=1$, $sn_{8}^{k_{2}}=1$, $sn_{10}^{k_{2}}=1$
$u_{i}^{k}$	$u_{i_{1}}^{k_{1}}\geq u_{i_{2}}^{k_{1}}$, $u_{i_{1}}^{k_{1}}\geq u_{i_{3}}^{k_{1}}$	$u_{i_{4}}^{k_{2}}\geq u_{i_{5}}^{k_{2}}$ or $u_{i_{5}}^{k_{2}}\geq u_{i_{5}}^{k_{2}}$

So $\sum_{k\in K}\sum_{i\in P}\sum_{j\in P\cup \{p+1\}}\pi_{i}\cdot x_{j,i}^{k}=15\times 5=75$, $\sum_{k\in K}\sum_{j\in P}vc^{k}\cdot x_{p+1,j}^{k}=1\times 2=2$, $\sum_{k\in K}tc^{k}\cdot \sum_{e\in E}le_{e}\cdot y_{e}^{k}=1\times (3+3+3+3+3+3+3+2+3+2+4)=32$, $\sum_{k\in K}tc^{k}\cdot \sum_{i\in P\cup \{p+1\}}\sum_{j\in P}lc_{i,j}\cdot x_{i,j}^{k}=1\times (3+3)=6$, $\sum_{k\in K}\sum_{n\in N}sc_{n}^{k}\cdot sn_{n}^{k}=1\times 9=9$. Total profit is 75−(2+32+6+9) = 26.

## 4 Solution approach

### 4.1 Neighborhood

Cordeau et al. [39] presented eight kinds of local search moves for OPDP: Couple-exchange, Block-exchange, Relocate-couple, Relocate-block, Multi-relocate, 2-opt-L, Double-bridge and Shake. Ribeiro et al. [40] and Ropke et al. [41] modified three large neighborhood removal heuristics and two large neighborhood insertion heuristics from Shaw P. [42], [43] and Potvin et al. [44] for OPDP. All those methods above may be of little efficiency for the OPDPSTRP in this paper, as the route structure of the OPDPSTRP is quite different from the classical OPDP, there are not so many pd-pairs can reinsert into a new route in the OPDPSTRP than in the classical OPDP. So five new neighborhood transform methods are presented for the OPDPSTRP.

Additionally, studies of Grimault et al. [45] and Ho et al. [7] show that solution feasibility of an OPDP is an important issue to the efficiency of the algorithm. So route structure feasibility judgement parameter of combining (RSFJPC, **Definition 3** in Section 3.4) were applied in neighborhood transform methods (*Insert*, *Spread*, *Point-delete*) to ensure that each selected pd-pair can be inserted into the selected route. The evolution of RSFJPC will be studied in Section 4.3.

#### 4.1.1 Insert

In *Insert*, pd-pair *i* (may be carried by other routes, may not be carried, with pickup point *p*_*i*_ and delivery point *d*_*i*_) is selected randomly and inserted into a new route *j* chosen according *pd*_*R*_*rs*_*judge*_*i*,*j*_ = 1.

**Fig 5:**
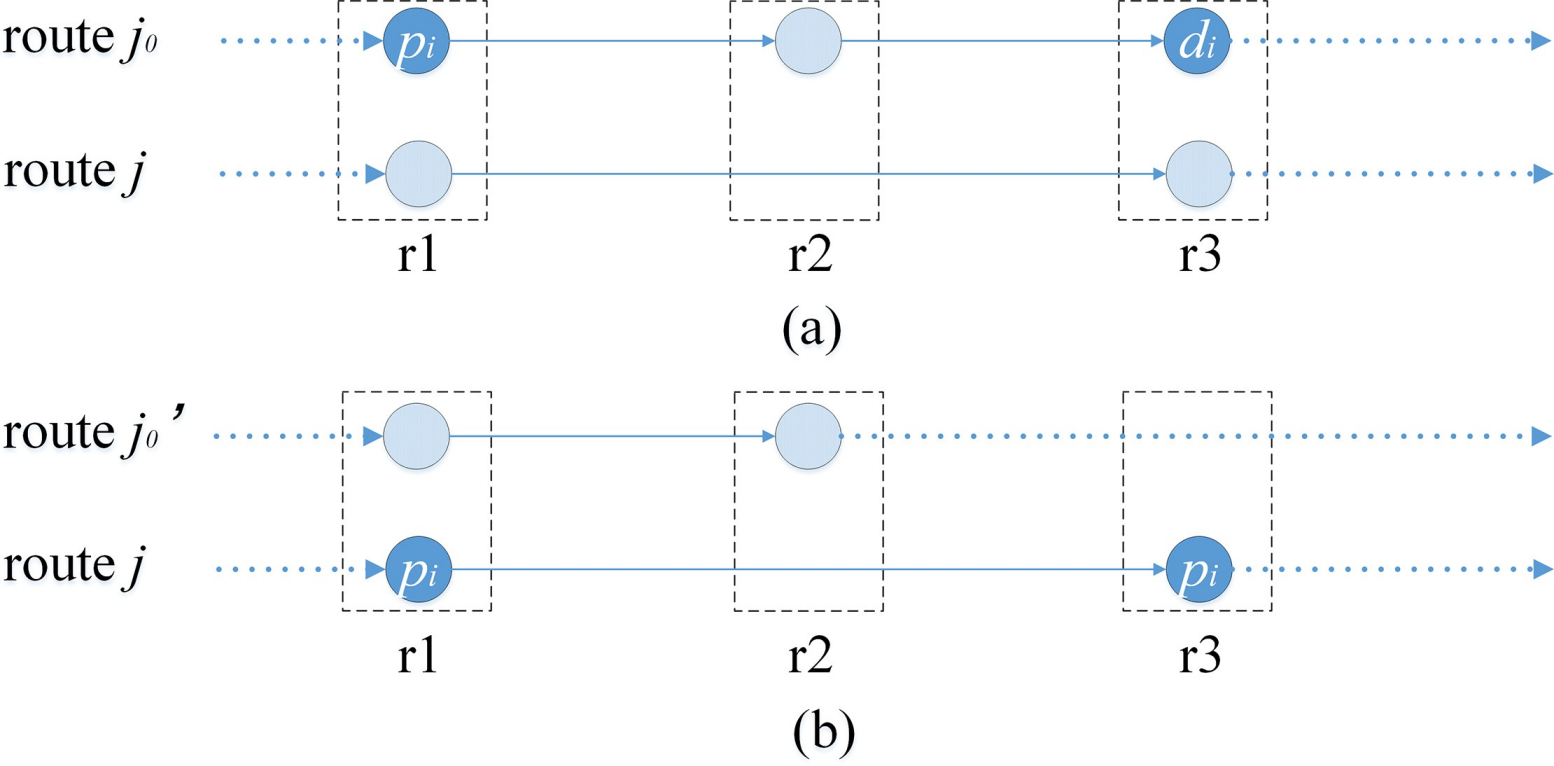
Construction methods for *Insert*.

As in Fig 5, pd-pair *i* is inserted into route *j*_2_ from route *j*_1_, the scheme showed in Fig 5(A) is replaced by Fig 5(B). Stop point *r*_3_ is removed from route *j*_1_ because there is no pd-pair requiring transportation from/to *r*_3_, while structure of route *j*_2_ remains the same. By reducing the number of stops, the route has been improved.

#### 4.1.2 Spread

In *Spread*, a pd-pair is selected and inserted into a new route as an Insert operation. Should the vehicle be overloaded, the success rate can be improved by choosing a new pd-pair *i* from route and transferring this into a new route *j* selected by *pd*_*R*_*rs*_*judge*_*i*,*j*_ = 1, and this cycle will continue until the vehicle is no longer overloaded, or if the cyclic number *k* exceed the preset iterative numbers controlling value *K*. The task of preset value *K* is to control the computing time of this operation.

**Fig 6:**
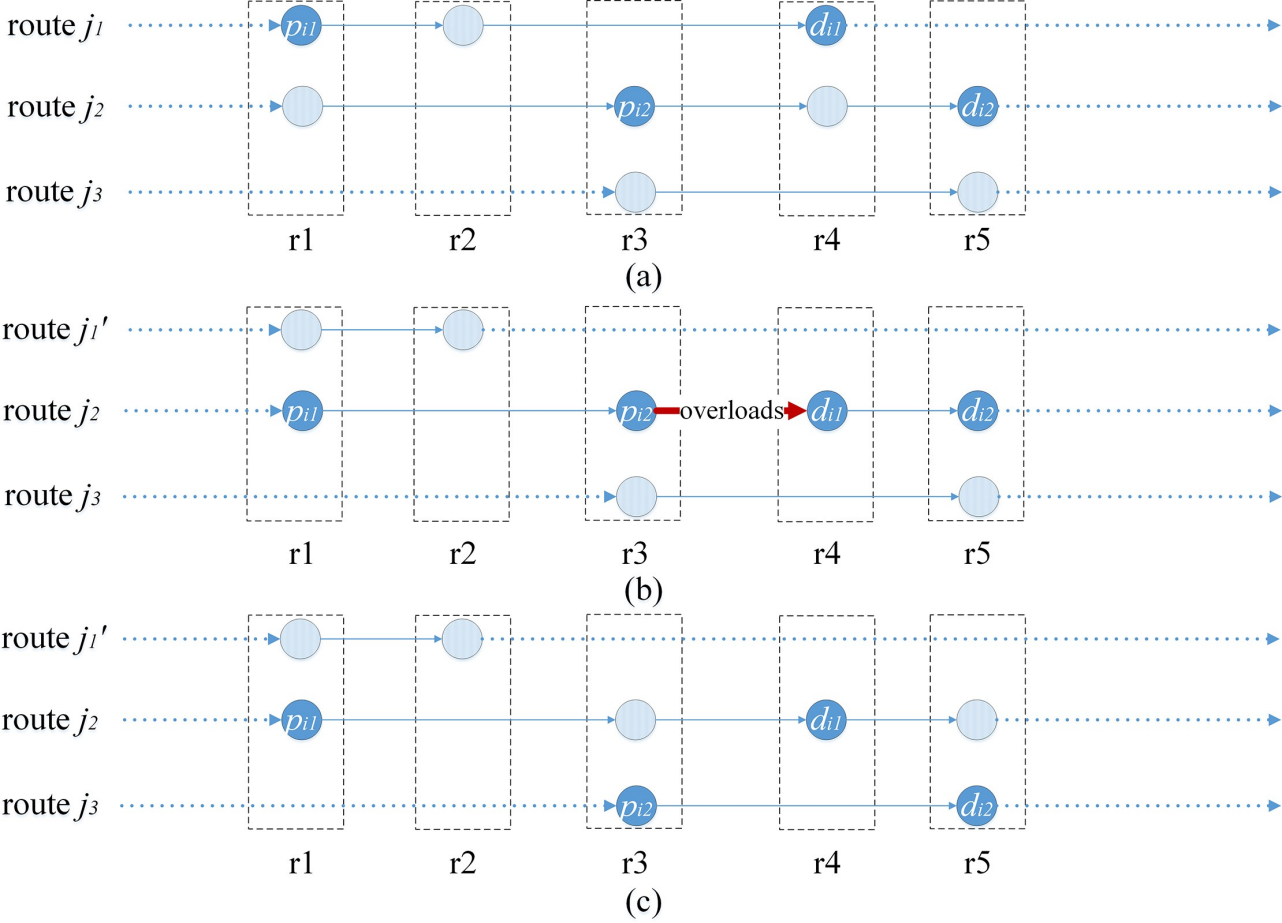
Construction methods for *Spread*.

As illustrated in Fig 6(A), pd-pair *i*_1_ transported through route *j*_1_ is inserted into route *j*_2_, and the new scheme is shown in Fig 6(B). Stop point *r*_4_ is deleted from route *j*_1_ because there is no pd-pair requiring transportation from/to *r*_4_, and the structure of route *j*_2_ remains the same. Since the vehicle is overloaded at section *r*_3_-*r*_4_ in route *j*_2_, pd-pair *i*_2_ is selected from route *j*_2_ and inserted into *j*_3_, and then the new scheme is obtained as Fig 6(C), in which the vehicle is no longer overloaded at section *r*_3_-*r*_4_, while the structure of route *j*_3_ remains unchanged, and the scheme is finally improved.

#### 4.1.3 Point-delete

*Point-delete* starts by choosing a route at random, before isolating the point with the minimal number picking stops and delivery stops on the route, and these pd-pairs *i*∈*P* are subsequently inserted into different routes *j* selected by *pd*_*R*_*rs*_*judge*_*i*,*j*_ = 1, thus making it possible to delete the point from the first route.

**Fig 7:**
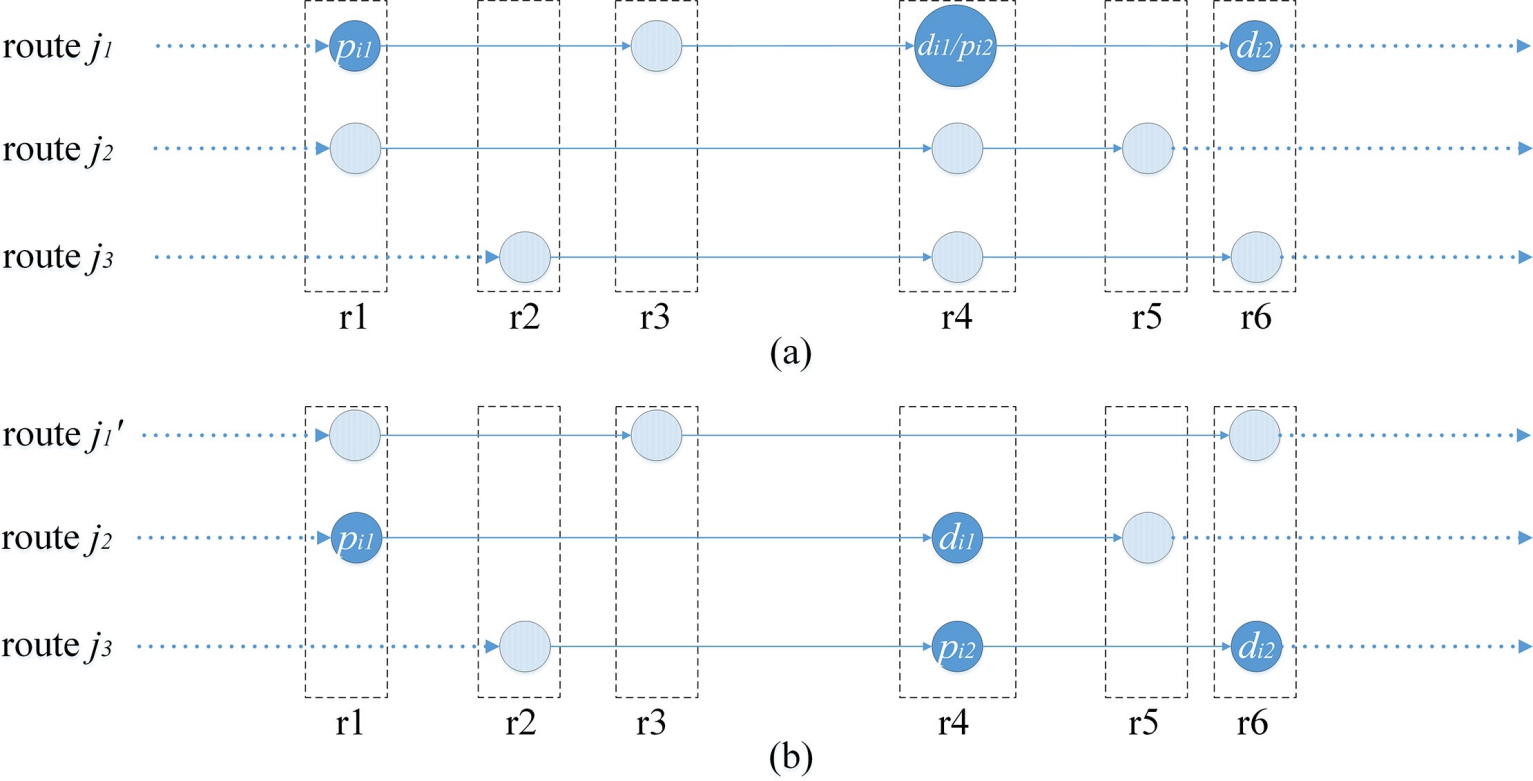
Construction methods for *Point-delete*.

As in Fig 7(A), *Point-delete* is operated on stop point *r*_4_ in route *j*_1_, pd-pair *i*_1_ and *i*_2_ are inserted into route *j*_2_ and *j*_3_ respectively, and then the new scheme is obtained as Fig 7(B). Stop point *r*_4_ is deleted from route *j*_1_ because there is no pd-pair requiring transportation from/to it, while the structure of routes *j*_2_ and route *j*_3_ remains the same. The scheme is improved due to the reduction of stop point *r*_4_.

#### 4.1.4 Route-delete

In the *Route-delete*, net incomes *ne*_*i*_ of each route *i* are computed; route *k* is selected according to the probability *P*_*k*_ = *ne*_*k*_/∑*ne*_*i*_ before being deleted from the scheme. All pd-pairs transported by route *k* are transferred to the state of non-carried. If there is no route with non positive net incomes, then the *Point-delete* should be executed.

#### 4.1.5 Reassign-vehicle

*Reassign-vehicle* is an Assignment Problem (AP) in which: *rv*_*i*,*j*_ = 1 means route *i* being transported by vehicle *j*, and *rv*_*benefit*_*i*,*j*_ is defined as its income. In this strategy, vehicles are reassigned to routes to achieve the best scheme by the Gurobi solver in Matlab.

#### 4.1.6 Opt(k) and perturbation

Since *Reassign-vehicle* may delivers the most significant change to the solution but cannot always improve the solution and requires more computer memory and time, it’s better to be chosen according to a low probability to perturb the local best solution, so a kind of *Perturbation* is proposed to shock the local best solution instead of *Reassign-vehicle*. In *Perturbation*, *Insert*, *Spread*, *Point-delete*, *Rout-delete* and *Reassign-vehicle* are chosen according to the ***operators choosing probabilities*** *p*_1_, *p*_2_, *p*_3_, *p*_4_ and *p*_5_ respectively.

Above all, *Insert*, *Spread*, *Point-delete*, *Rout-delete*, and *Perturbation* are defined as operators *opt*(*k*)(*k* = 1,2,3,4 and 5) in this paper.

### 4.2 Route construction methods of neighborhood

Operators for routes construction and adjusting can be classed into three separate categories: pd-pair insertion, pd-pair deletion, and route deletion.

#### 4.2.1 Route construction method for pd-pair insert

Muelas et al. [28] arrayed two pd-pairs by comparing the distance between pickup/delivery points of them based on four cases. Different from their methods, in this paper, a new approach is proposed to find the right locations in a new route and insert a pd-pair into it.

Firstly, it is evident that the section between any two neighbor stop points *r*_*i*_ and *r*_*i*+1_ in route *R* is the shortest path since all pd-pairs must be transported along the shortest path. Once pd-pair *k* needs to be inserted into route *R*(*r*_1_−*r*_2_−*r*_3_…*r*_*n*_), we have to find the right inserting location of pickup point *p*_*k*_ and delivery point *d*_*k*_ in route *R* first. Let $l_{r_{i},r_{j}}$ is the distance between *r*_*i*_ and *r*_*j*_ and let $l_{R(r_{i}-r_{j}-r_{k})}=l_{r_{i},r_{j}}+l_{r_{j},r_{k}}$ is the length of section *r*_*i*_−*r*_*j*_−*r*_*k*_ in route *R*. A point *r*_*i*_ meeting the condition $l_{r_{i},r_{i+1}}=l_{r_{i},p_{k}}+l_{p_{k},r_{i+1}}$ and a point *r*_*j*_ meeting the condition $l_{r_{j},r_{j+1}}=l_{r_{j},d_{k}}+l_{d_{k},r_{j+1}}$ need to be found in route *R*. Finally, *p*_*k*_ and *d*_*k*_ need to be inserted into the rear of the two points respectively, and we may get three types of results as follows: both *r*_*i*_ and *r*_*j*_ can be found, only one of *r*_*i*_ and *r*_*j*_ can be found, neither *r*_*i*_ nor *r*_*j*_ can be found.

Both *r*_*i*_ and *r*_*j*_ can be found:

➢*r*−*p*−*r*−*d*−*r*

**Fig 8:**
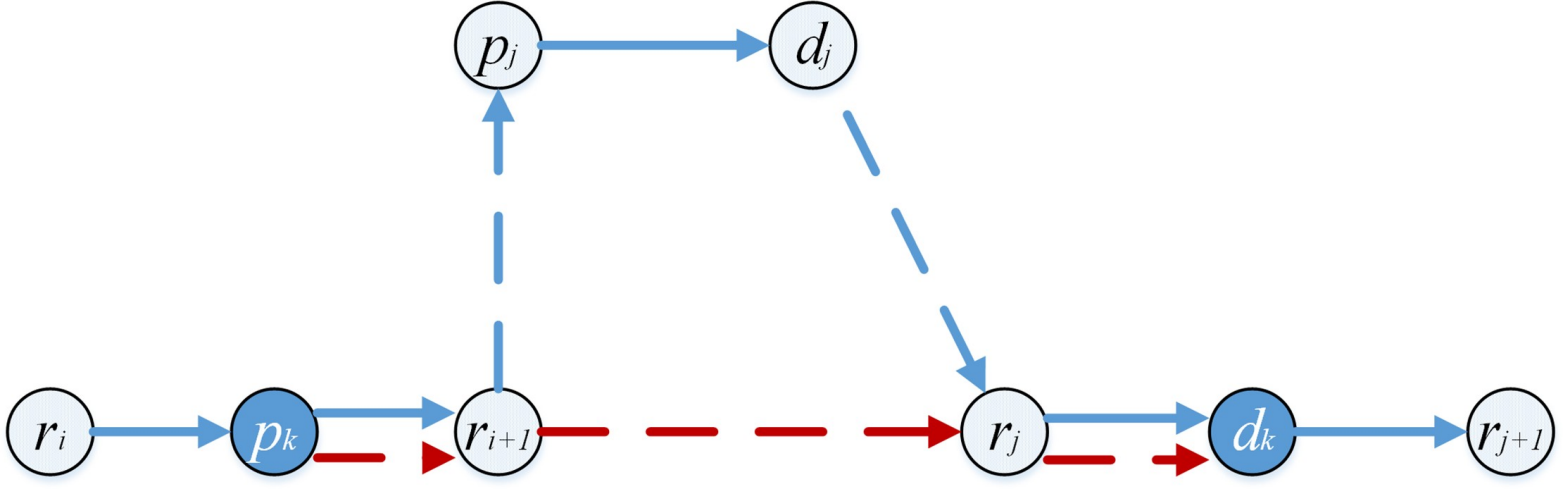
Unfeasible case-Both *r*_*i*_ and *r*_*j*_ are found and *i*<*j*.

If both *r*_*i*_ and *r*_*j*_ can be found, and *i*<*j*, the structure form of the new route *R*' must be *r*−*p*−*r*−*d*−*r*. In order to ensure feasibility of the route structure, pd-pair *k* should be transported along the shortest path in route *R*’, that is, $l_{R'(p_{k}-r_{i+1}\ldots r_{j}-d_{k})}=l_{p_{k},d_{k}}$. Otherwise, pd-pair *k* cannot be inserted into *R*. As in Fig 8, $l_{R'(p_{k}-r_{i+1}\ldots r_{j}-d_{k})}\neq l_{p_{k},d_{k}}$, so the route structure is unfeasible.

➢*r*−*p*−*d*−*r*

**Fig 9:**
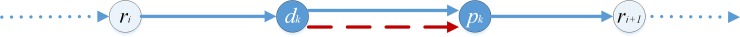
Unfeasible case-Both *r*_*i*_ and *r*_*j*_ are found and *i* = *k*.

If both *r*_*i*_ and *r*_*j*_ can be found, and *i* = *j*, the route structure form of *R*' must be *r*−*p*−*d*−*r*. In order to ensure feasibility of the route structure, *r*_*i*_−*p*_*k*_−*d*_*k*_ must be the shortest path, that is, $l_{R'(r_{i}-p_{k}-d_{k})}=l_{r_{i},d_{k}}$. Otherwise, pd-pair *k* cannot be inserted into route *R*. As in Fig 9, $l_{R'(r_{i}-p_{k}-d_{k})}\neq l_{r_{i},d_{k}}$, so the route structure is unfeasible.

➢ Other cases

**Fig 10:**
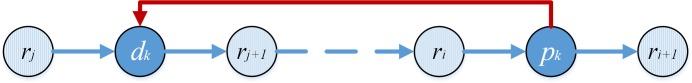
Unfeasible case-Both *r*_*i*_ and *r*_*j*_ are found and *i*>*j*.

If both *r*_*i*_ and *r*_*j*_ are found, and *i*>*j*, pd-pair *k* are opposite to route *R* and cannot be inserted into it, as in Fig 10.

**Fig 11:**
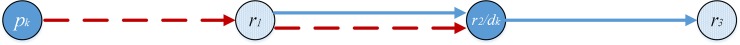
Case-*r*_2_ is *d*_*k*_.

Because there is only one shortest path between any two nodes in this paper, if there is more than one *r*_*i*_ or *r*_*j*_ satisfying the criteria, it must be sure that *p*_*k*_ or *d*_*k*_ is in route *R* already, then point *p*_*k*_ or *d*_*k*_ need not be inserted into it repeatedly. As in Fig 11, $l_{r_{1},r_{2}}=l_{r_{1},d_{k}}+l_{d_{k},r_{2}}$ and $l_{r_{2},r_{3}}=l_{r_{2},d_{k}}+l_{d_{k},r_{3}}$, *r*_2_ is *d*_*k*_, so *R*(…*r*_1_−*r*_2_−*r*_3_…) remains the same after inserting *d*_*k*_ into it.

Only one of *r*_*i*_ and *r*_*j*_ can be found:

➢*r*−*p*−*r*−*d*

**Fig 12:**
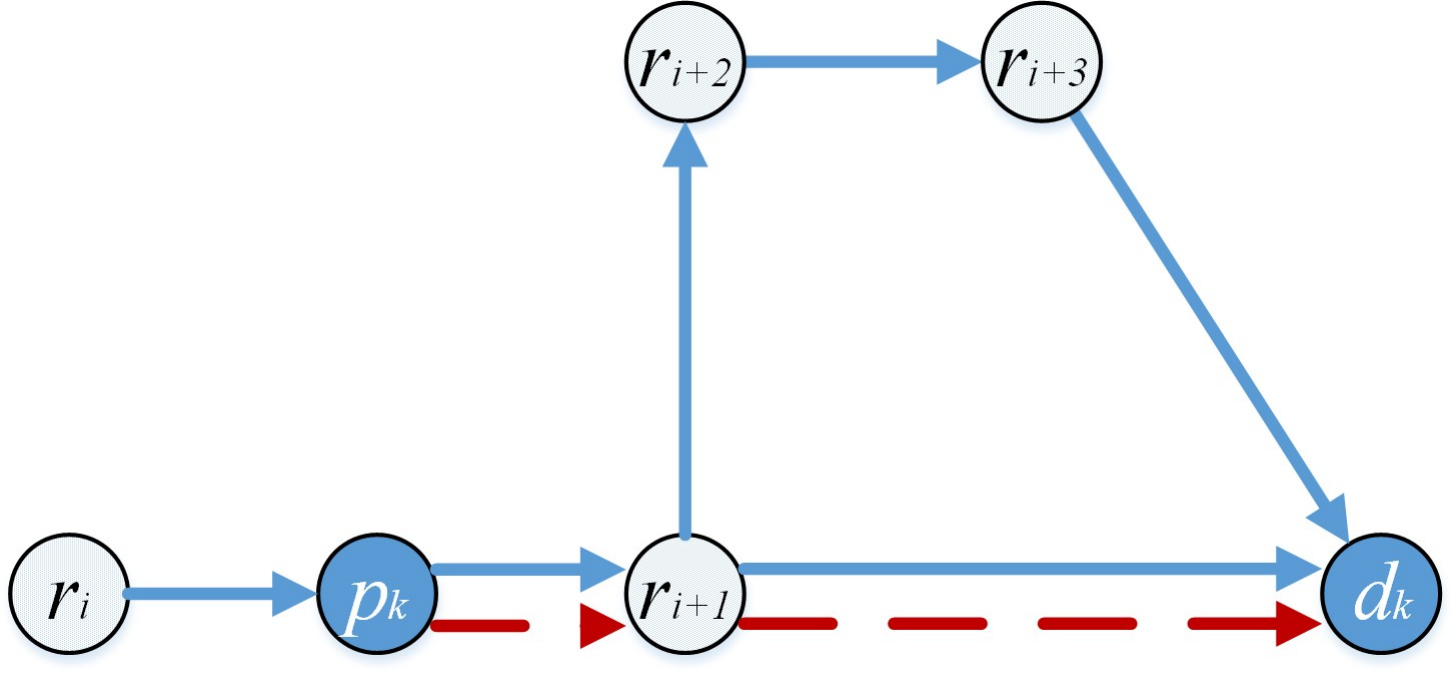
Unfeasible case-Only *r*_*i*_ is found.

If only *r*_*i*_ can be found, the route structure form of *R*' must be *r*−*p*−*r*−*d*. Pd-pair *k* must be transported along the shortest path in route *R*', that is, $l_{R'(p_{k}-r_{i+1}\ldots r_{i+3}-d_{k})}=l_{p_{k},d_{k}}$. Otherwise, pd-pair *k* cannot be inserted into *R*. As in Fig 12, $l_{R'(p_{k}-r_{i+1}\ldots r_{i+3}-d_{k})}=l_{p_{k},d_{k}}$, so the route structure is unfeasible.

➢*p*−*r*−*d*−*r*

**Fig 13:**
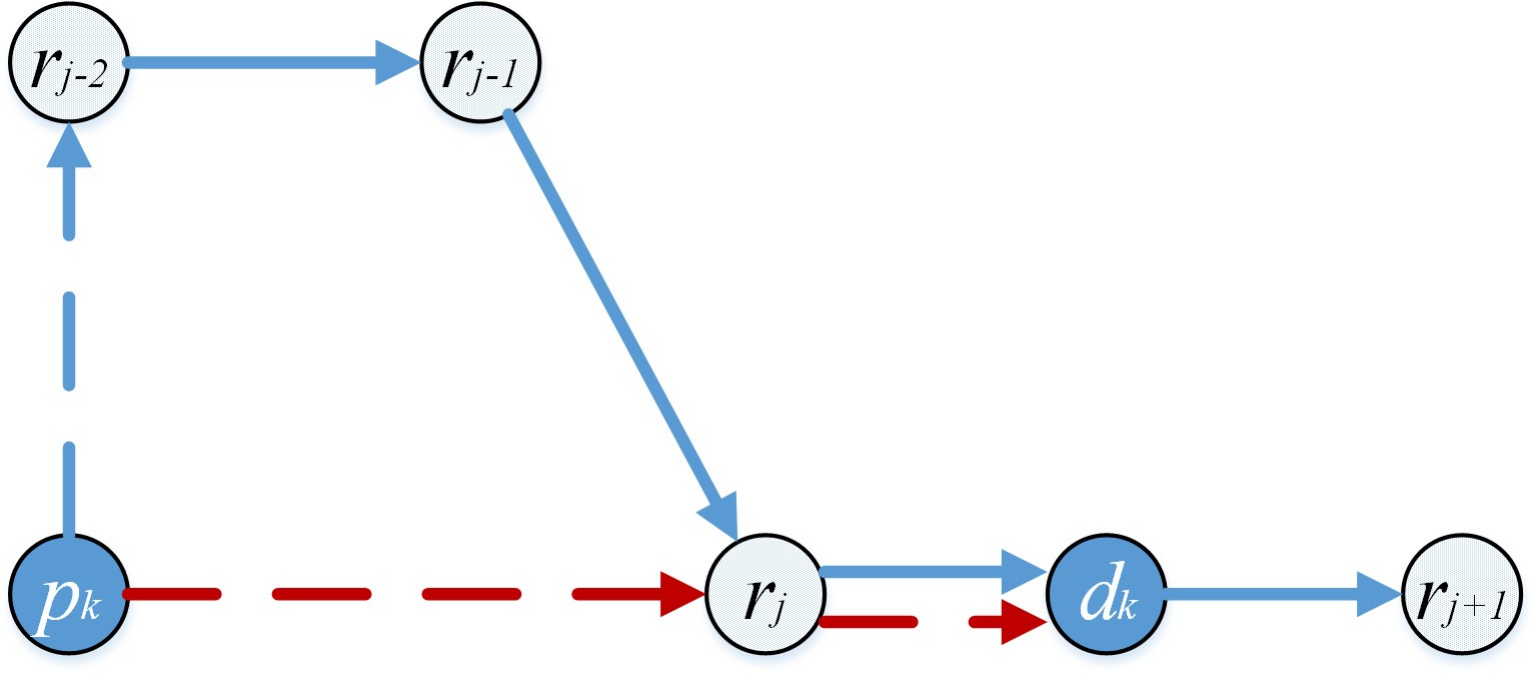
Unfeasible case-Only *r*_*j*_ are found.

If only *r*_*j*_ can be found, the route structure form of *R*' must be *p*−*r*−*d*−*r*. Pd-pair *k* must be transported along the shortest path in route *R*', that is, $l_{R'(p_{k}-r_{j-2}\ldots r_{j}-d_{k})}=l_{p_{k},d_{k}}$. Otherwise pd-pair *k* cannot be inserted into route *R*. As in Fig 13, $l_{R'(p_{k}-r_{j-2}\ldots r_{j}-d_{k})}\neq l_{p_{k},d_{k}}$, so the route structure is unfeasible.

Neither *r*_*i*_ nor *r*_*j*_ can be found:

➢*p*−*r*−*r*−*d*

**Fig 14:**
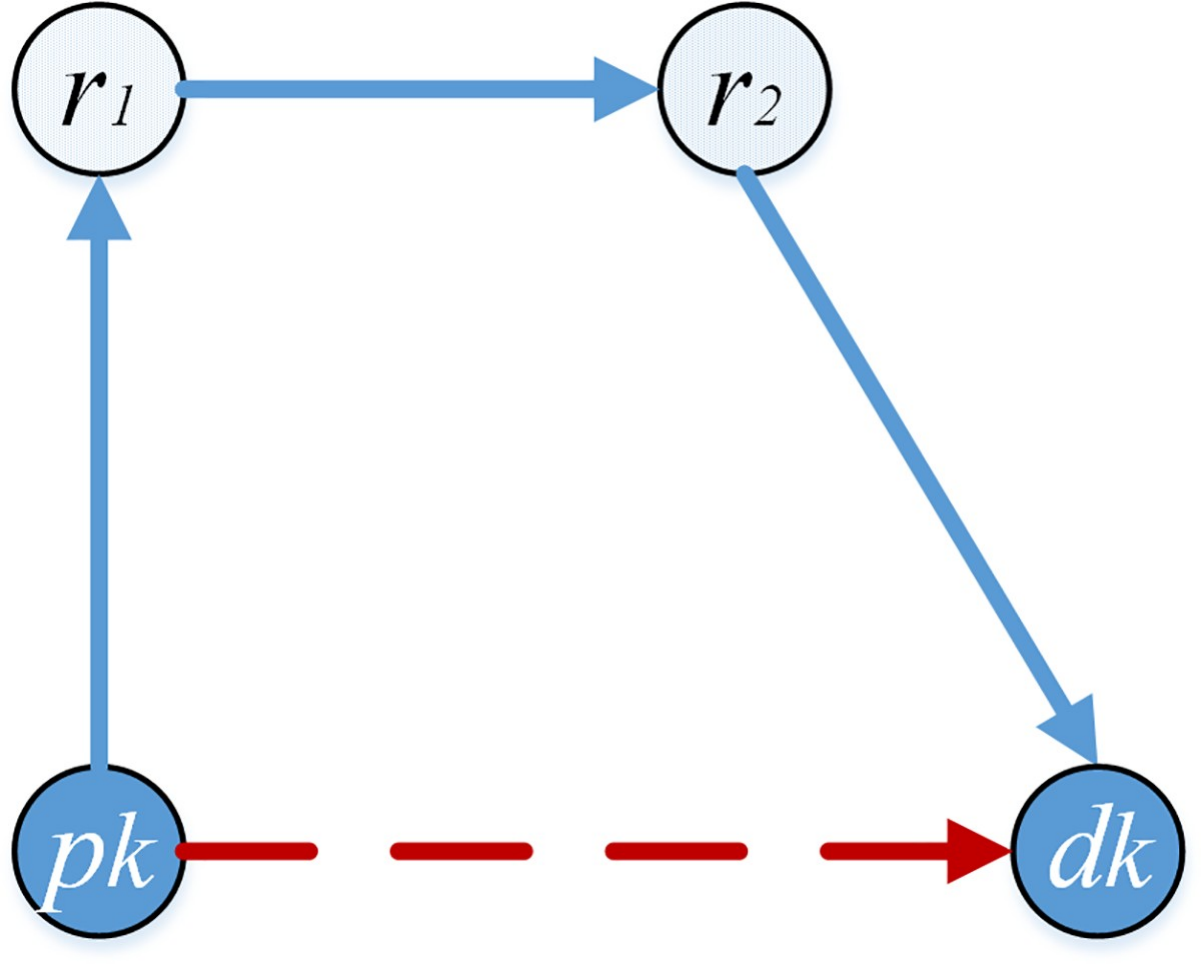
Unfeasible case I-Neither *r*_*i*_ nor *r*_*j*_ are found.

If none of *r*_*i*_ and *r*_*j*_ can be found, and $l_{R'(p-r_{1}\ldots r_{n}-d)}=l_{p_{k},d_{k}}$, the route structure form of route *R*' must be *p*−*r*−*r*−*d*, pd-pair *k* is transported along the shortest path in route *R*', and the route structure must be feasible, that is, pd-pair *k* can be inserted into route *R*. As in Fig 14, $l_{R'(p-r_{1}\ldots r_{n}-d)}\neq l_{p_{k},d_{k}}$, so the route structure is unfeasible.

➢*p*−*d*−*r*−*r*

**Fig 15:**
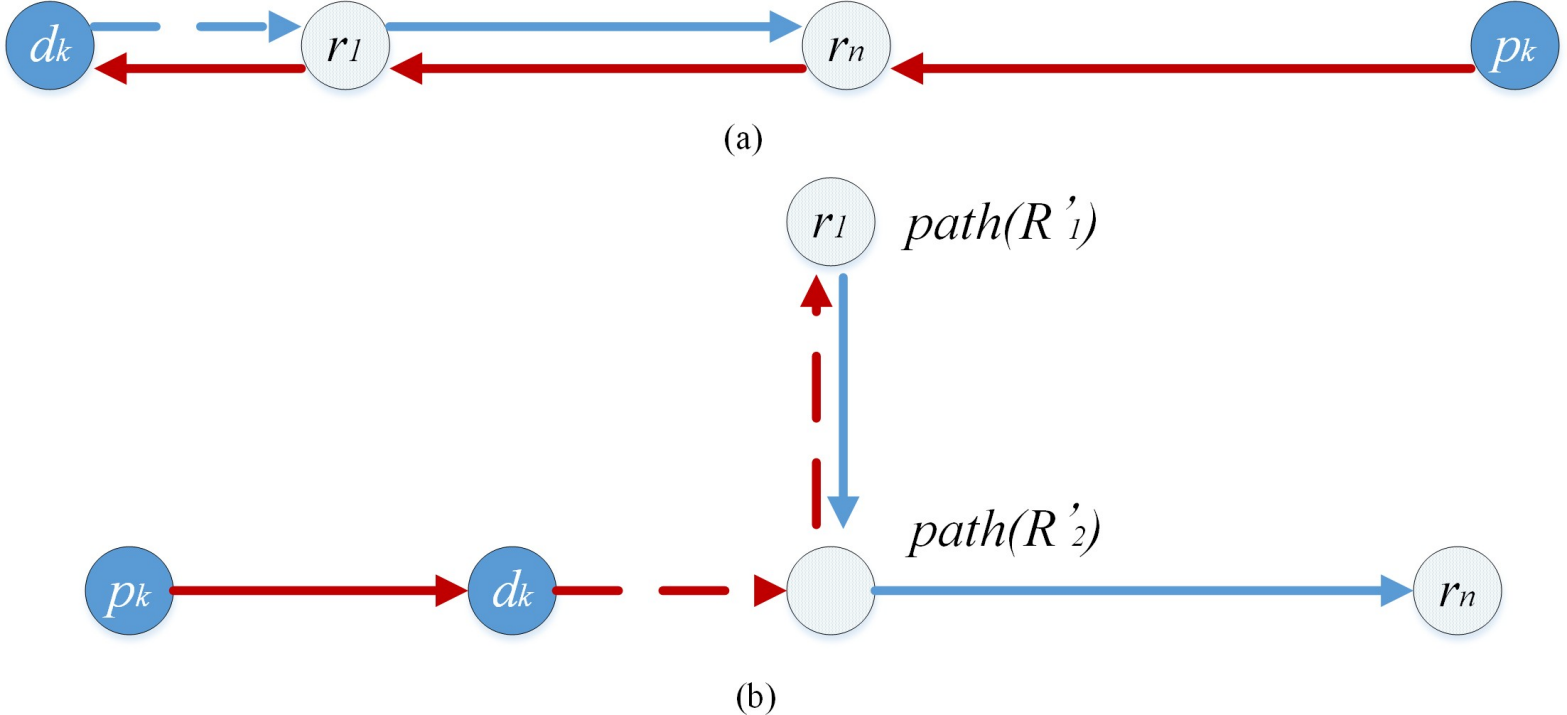
Unfeasible case II-Neither *r*_*i*_ nor *r*_*j*_ are found.

If neither *r*_*i*_ nor *r*_*j*_ are found, and $l_{R'(p-r_{1}\ldots r_{n}-d)}\neq l_{p_{k},d_{k}}\cap l_{d_{k},r_{1}}<=l_{r_{n},p_{k}}$, pd-pair *k* must be connected to the front of route *R*, the route structure form of *R*' must be *p*−*d*−*r*−*r* to minimize the length of *R*'. Set *path*(*R*) is the vehicle path which consists of all pass points in route *R*. Pd-pair *k* must be transported along the shortest path and each point must not be visited more than once in route *R*' to ensure feasibility of the route structure, that is, $l_{p_{k},r_{1}}=l_{p_{k},d_{k}}+l_{d_{k},r_{1}}$ and $l_{d_{k},path(R{'}_{2})}=l_{d_{k},path(R{'}_{1})}+l_{path(R{'}_{1}),path(R{'}_{2})}$. Otherwise pd-pair *k* cannot be inserted into route *R*. As in Fig 15(A), the route structure is unfeasible because $l_{p_{k},r_{1}}\neq l_{p_{k},d_{k}}+l_{d_{k},r_{1}}$. As in Fig 15(B), the route structure is unfeasible because $l_{d_{k},path(R{'}_{2})}\neq l_{d_{k},path(R{'}_{1})}+l_{path(R{'}_{1}),path(R{'}_{2})}$.

➢*r*−*r*−*p*−*d*

**Fig 16:**
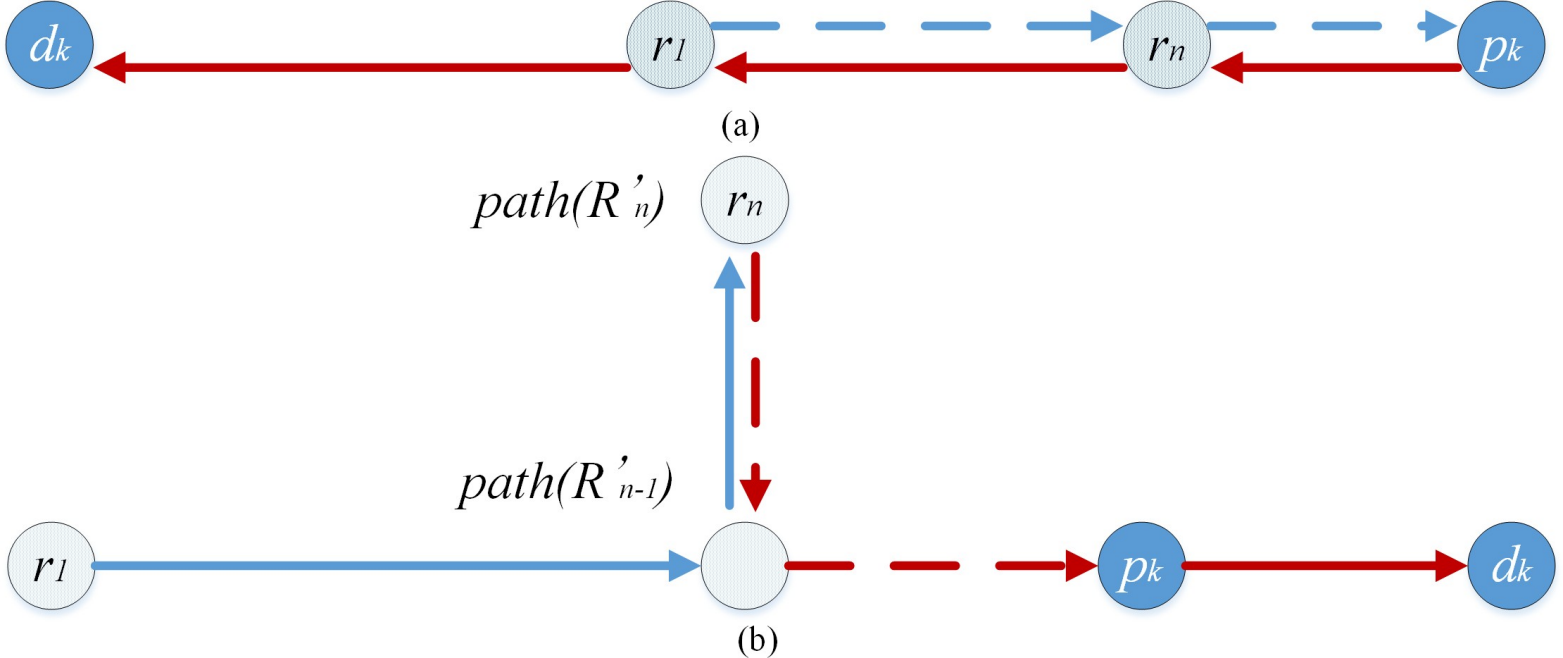
Unfeasible case-III-Neither *r*_*i*_ nor *r*_*j*_ is found.

If neither *r*_*i*_ nor *r*_*j*_ are found, and $l_{R'(p-r_{1}\ldots r_{n}-d)}\neq l_{p_{k},d_{k}}\cap l_{d_{k},r_{1}}>l_{r_{n},p_{k}}$, pd-pair *k* must be connected to the back of route *R*, and the route structure form of route *R*' must be *r*−*r*−*p*−*d* to minimize the length of route *R*’. Pd-pair *k* must be transported along the shortest path and each point must not be visited more than once in route *R*' to ensure feasibility of the route structure, that is, $l_{r_{n}{,d}_{k}}=l_{r_{n},p_{k}}+l_{p_{k},d_{k}}$ and $l_{path(R{'}_{n-1}),p_{k}}=l_{path(R{'}_{n-1}),path(R{'}_{n})}+l_{path(R{'}_{n}),d_{k}}$. As in Fig 16(A), the route structure is unfeasible because $l_{r_{n},d_{k}}\neq l_{r_{n},p_{k}}+l_{p_{k},d_{k}}$. As in Fig 16(B), the route structure is unfeasible because $l_{path(R{'}_{n-1}),p_{k}}\neq l_{path(R{'}_{n-1}),path(R{'}_{n})}+l_{path(R{'}_{n}),d_{k}}$.

#### 4.2.2 Route construction method for pd-pair delete

Point *p*_*k*_ or *d*_*k*_ needs to be deleted if there is no other pd-pair that needs picking up from it or delivering to it after deleting pd-pair *k* from route *R*. Otherwise, it should remain in route *R*.

#### 4.2.3 Route construction method for route delete

For Route-delete strategy, all pd-pairs are removed and route is deleted.

### 4.3 Evolution of route structure feasibility judgement matrix

#### 4.3.1 Evolution theories for route structure feasibility judgement matrix

At each local search move step, RSFJMC [*pd*_*combine*_*rs*_*judge*_*i*,*j*_] will remain the same, while RSFJMI [*pd*_*R*_*rs*_*judge*_*i*,*j*_] will change with the changes of route *j*. In order to update [*pd*_*R*_*rs*_*judge*_*i*,*j*_] quickly, some theories will be studied as follows.

**Theorem 1:** A necessary and sufficient condition of which pd-pair *k* can be inserted into route *m* according route structure is that pd-pair *k* can be combined with all pd-pair transported by route *m* according route structure.

**Lemma 1:** If pd-pair *i* is inserted into route *m*, and a new route *m*' is obtained, pd-pair *j* which cannot be combined with pd-pair *i* according route structure cannot be inserted into route *m*' according route structure.

**Lemma 2:** Assuming pd-pair *k* cannot be inserted into route *m* according route structure. If all pd-pairs which cannot be combined with pd-pair *k* according route structure are deleted from route *m*, then pd-pair *k* can be inserted into the new route *m*' evolving from route *m* according route structure.

**Lemma 3:** A new route *R*_*m*_ can be constructed with pd-pairs according route structure, which can be combined with each other according route structure.

The proofs of **Theorem 1**, **Lemma 1**, **Lemma 2**, and **Lemma 3** are provided in S2 Appendix.

#### 4.3.2 Evolution methods for route structure feasibility judgement matrix

As RSFJMC will remain the same at each local search move step, while RSFJMI will change as routes evolve. If RSFJMI is updated at each step, too much computing time will be spent on carried out the algorithm. In this section, evolution methods of RSFJMI will be studied to ensure that the calculation time is acceptable.

According to the route construction methods in Section 4.2, the evolution of route structures can be grouped into two categories: pd-pair deletion and pd-pair insertion. The RSFJMC and RSFJMJ can be calculated after an initial feasible solution is generated. At each local search move step, the RSFJMI will be updated by methods as follows.

**4.3.2.1 Evolution of RSFJMI for pd-pair insertion.** When pd-pair *i* is inserted into route *k*, and a new route *k*' is acquired. In order to reduce calculating time, let *L*_1_ = {*l*|[*pd*_*combine*_*rs*_*judge*](*l*,*i*)==0}, [*pd*_*R*_*rs*_*judge*](:,*k*') for route *k*’ can be updated by formula (18) according to **Lemma 1**.

$$[pd\_R\_rs\_judge](j,k')=0{\;\forall j\in L}_{1}$$ (18)

**4.3.2.2 Evolution of RSFJMI for pd-pair deletion.** When pd-pair *i* is deleted form route *k*, and a new *k*' is acquired. In order to reduce calculating time, Let *L*_1_ = {*l*|[*pd*_*combine*_*rs*_*judge*](*l*,*i*)==0}, and let *L*_2_ = {*p*_*l*_−*d*_*l*_}, a set of pd-pairs transported by route *k*'. [*pd*_*R*_*rs*_*judge*](:,*k*') for route *k*' can be updated by formula (19) according to **Theorem 1** and **Lemma 2**.

$$[pd\_R\_rs\_judge](j,k')=\prod_{l\in L_{2}}pd\_combine\_rs\_judge_{j,l}\;\forall j\in L_{1}$$ (19)

Additionally, **Lemma 3** can be used when new routes are generated, such as for the generation of new routes.

### 4.4 Generation of initial solution

It is important to obtain a higher-level initial solution for such a large-scale and complex problem. In this paper, a generation method of initial solution is proposed, based on the idea of maximum saving. *p*_*i*_ and *d*_*i*_ are defined as pickup point and delivery point of pd-pair *i* correspondingly. *l*(*k*,*l*) is defined as the length between point *k* and point *l*.

**Fig 17:**
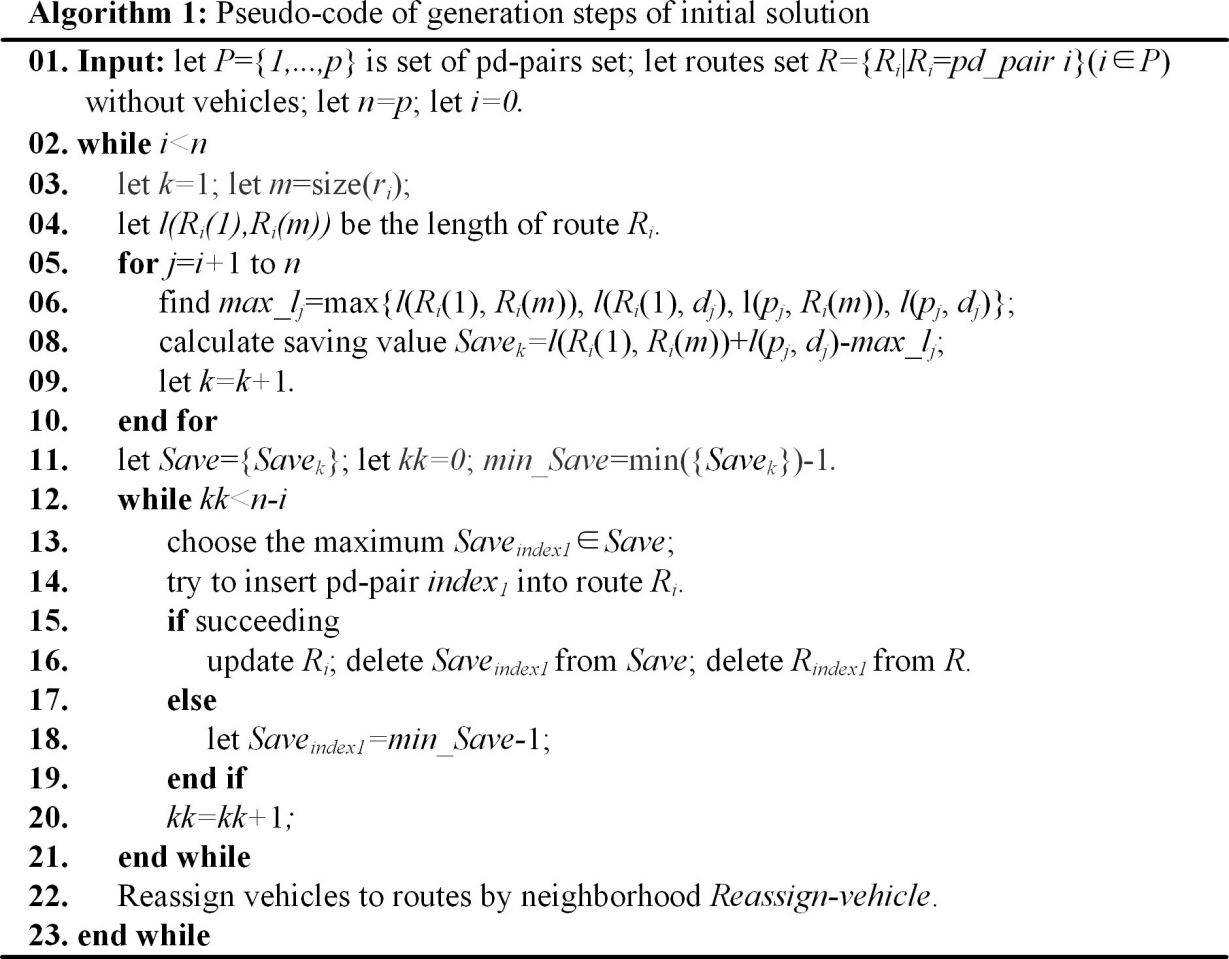
Algorithm 1. Pseudo-code of generation steps of initial solution.

Generation steps of initial solution are presented in **Algorithm 1 (Fig 17)**.

### 4.5 Algorithm

Many Local Search (LS) meta-heuristics are studied for the VRP, such as the ALNS proposed by Ropke et al. [41], Pisinger et al. [54], Aksen et al. [55] and Li et al. [25]. Multi-start ILS (MS_ILS), consisting in restarting an ILS from several initial solutions to diversify the search, is proposed by Prins et al. [56], Li et al. [47], Rivera et al. [57], Sana et al. [58] and Gustavo et al. [59]. The VND, a kind of local search in which wider and wider neighborhoods are successively explored, is studied by Salehipour et al. [60]. Variable Neighborhood Search (VNS), which is similar to the VND but with perturbation, is studied by Hansen et al. [61], which can reduces the impact of the initial solution on the algorithm performance (Brimberg et al. [62]).

Since the major neighbourhood operator is pd-pair insertion and a choosing pd-pair need be inserted a fixed position in a route at each local search move step in the OPDPSTRP, solutions are not easy to be changed and always cannot be improved by only one operator obviously. That is to say that new methods which is different from traditional LS need be proposed for the OPDPSTRP. A basic VND and a basic VNS are proposed for the OPDPSTRP based on the above five neighborhoods. A new MS_VND and a new MS_VNS are developed to improve the efficiency of the proposing neighborhoods and algorithms in this paper.

#### 4.5.1 VND and VNS

**Fig 18:**
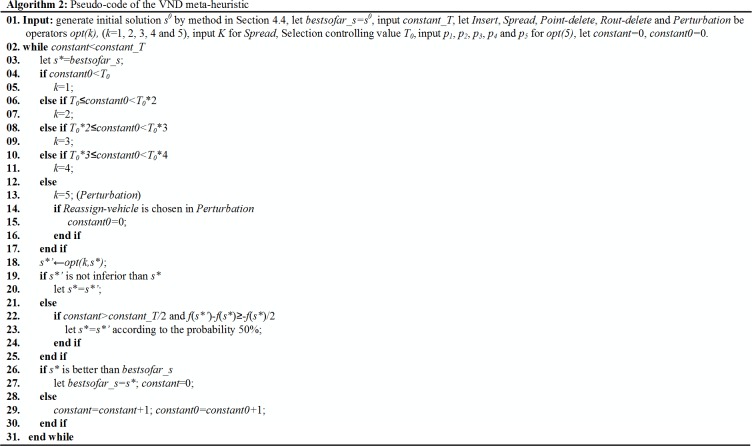
Algorithm 2. Pseudo-code of the VND meta-heuristic.

**Fig 19:**
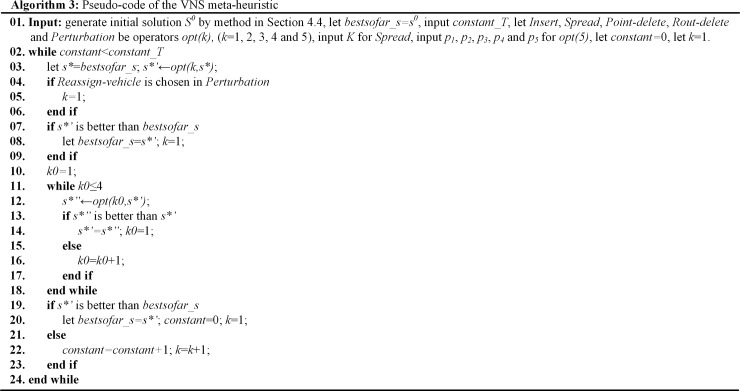
Algorithm 3. Pseudo-code of the VNS meta-heuristic.

A VND and a VNS are processed to solve the OPDPSTRP, Pseudo-code of them are presented as in **Algorithm 2 (Fig 18)** and **Algorithm 3 (Fig 19)**.

#### 4.5.2 MS_VND and MS_VNS

In order to achieve a near optimal solution of this problem, we propose a new MS_VND metaheuristic and a new Multi-Start Variable Neighborhood Search (MS_VNS) metaheuristic.

**Fig 20:**
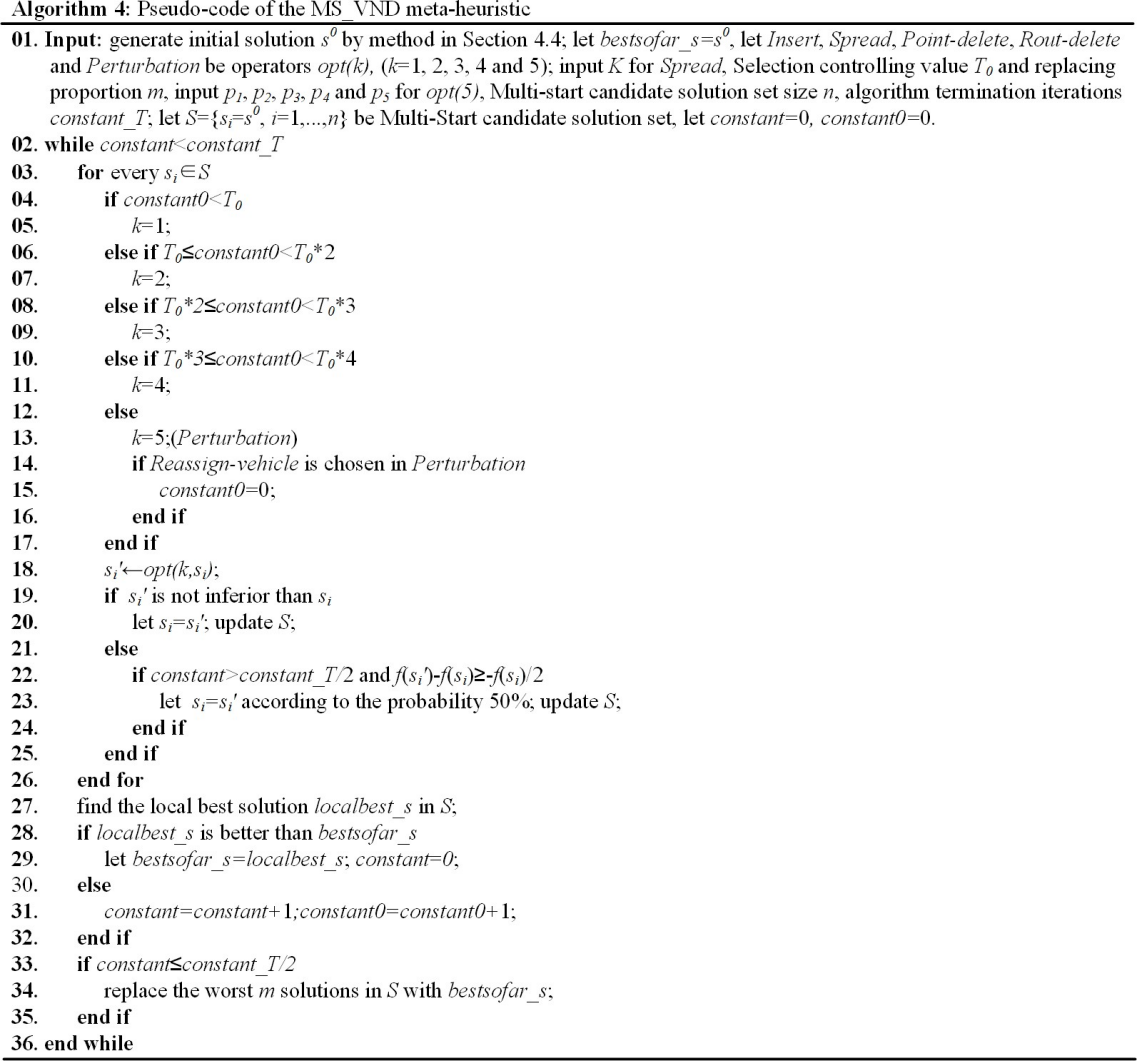
Algorithm 4. Pseudo-code of the MS_VND meta-heuristic.

The steps of MS_VND in this paper are presented as in **Algorithm 4 (Fig 20)**.

**Fig 21:**
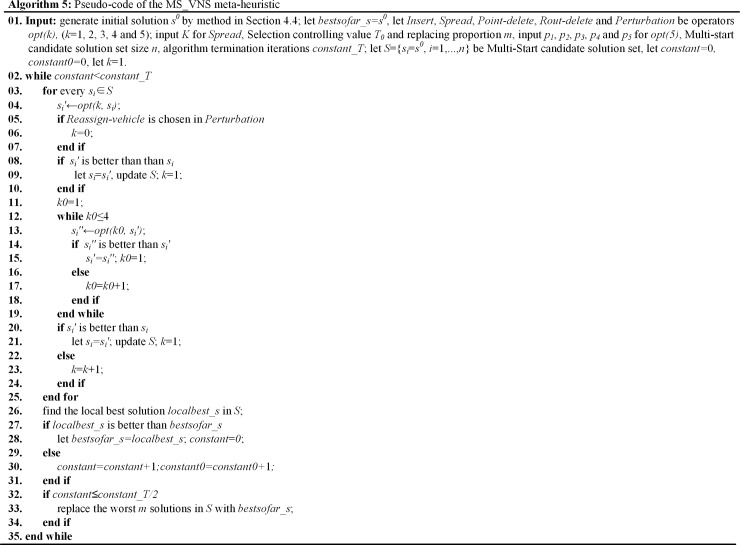
Algorithm 5. Pseudo-code of the MS_VNS meta-heuristic.

The steps of MS_VNS in this paper are presented as in **Algorithm 5 (Fig 21)**.

As in **Algorithm 4** and **Algorithm 5**, the MS_VND and the MS_VNS have been improved in the following ways: (1) A Multi-start candidate solution set with size of *n* is acquired from the initial solution and updated to diversify the search, and parts of worse candidate solutions are replaced by the best solution according ***replacing proportion m*** if the solution is improving. (2) Five new operators (*opt1*, *opt2*, *opt3*, *opt4*, *opt5*) are utilized to improve the solution, which is different from traditional VNS. (3) In MS_VND, each candidate solution is transformed only once at a step (Multi-start-candidate-solution and One-operator), which is different from traditional LS (One-candidate-solution and Local search). (4) A new local solution inferior to the primary one can also be accepted on the basis of two hypotheses: the iterations of that the solution keep the same are more than half of the presetting value, and the evaluation of the new solution is not drastically worse than the primary one.

Changes and complexity ranking (from low to high) of operators can be primarily concluded as: *Insert*<*Spread*<*Point-delete*<*Route-delete*<*Perturbation* according to the features of them. And this ranking will be tested further in Section 5.

## 5 Instances and computational results

To evaluate the performance of the VNS, the VND, the MS_VND and the MS_VNS, we designed 84 of the OPDPSTRP instances partitioned in small size connected graphs (3×4), medium size connected graphs (6×8) and large size connected graphs (10×10) at random. The Gurobi MIP solver (version 7.5.2) was used to compare with these approaches proposed in this paper. The following sections will describe the generation of instances, list the parameter values used in these approaches, and provide test results and optimality gaps.

### 5.1 Generation of instances

Each instance name has a format *m*0×*m*1−*m*2−*m*3−*m*4−*L*/*H*, in which *m*0×*m*1 is the size of a connected graph, each edge in this graph is deleted according to 1/*m*2, 1/*m*3 is the pd-pairs generation probability between each two nodes, 1/*m*4 is the vehicle generation percentage for each node and *L*/*H* is low or high cost. Consider instance *3*×*4-10-3-3-L* as an example: the size of the incomplete digraph is 3×4(12 nodes and 144 node-pairs), the deletion probability of each edge is 1/10, pd-pair generation probability between each two nodes is 1/3, the vehicle generation percentage for each node is 1/3 and *L* means low cost. It has been checked that there is only one shortest path between any two nodes in each graph. Data of these 84 instances can be found in S3 Appendix.

### 5.2. Parameter setting

All experiments were conducted on a desktop equipped with an Intel(R) Core(TM) i7-4510U 2.00 gigahertz processor and 8 Gigabyte RAM. The operating system of this PC is a 64-bit Window 8. The Gurobi solver 7.5.2 was used to solve the MIP model, and all algorithms in this paper was coded in Matlab R2015a.

The MIP model is solved by the Gurobi solver with termination conditions wherein computing time is over 108000 seconds or the Gurobi_Gap (the gap between the best feasible objective value and the upper bound of the MIP model, which will be introduced in Section 5.3) is less than 5%. The long preset time aims to ensure that the Gurobi solver can obtain at least one feasible solution served as a comparison indicator with the proposed approaches, although in some cases it failed to achieve this goal. We have provided the upper bounds found by Gurobi solver as well, for a more in depth reference to the performance of the proposed approaches.

The parameter setting of the algorithms have been tested based on 9 instances mention in S4 Appendix (including small size connected graphs, medium size connected graphs and large size connected graphs), the results are studied in S4 Appendix. The algorithms parameter setting is tuned by determining a trade off between solution quality and CPU time after numerous experiments.

According to S4 Appendix, the operators sequence is determined as *Insert*, *Spread*, *Point-delete*, *Rout-delete* and *Perturbation* (*k* = 1, 2, 3, 4 and 5) in these approaches, and the values of the parameters are gathered in Table 4.

**Table 4:** Parameter setting for the VND, the VNS, the MS_VND and the MS_VNS.

Symbol	Definition	Value
*N*	Size of Multi-Start candidate solution set	90
*constant*_*T*	Algorithm termination iterations	*constant*_*T* = *exp*(−20/(2+*num*_*pd*_*pairs*))*700
*T*_0_	Selection controlling value	20
*K*	Iterative numbers controlling value for ***Spread***	3
*p*_*k*_	Operator choosing probabilities in ***Perturbation***	9/24, 7/24, 1/24, 1/24, 6/24 for *Insert*, *Spread*, *Point-delete*, *Rout-delete*, and *Reassign-vehicle*
*m*	Replacing proportion for Multi-Start solution set	1/8

Note: *num_pd_pairs* in the “Value” column of the *constant_T* means the number of pd-pairs.

### 5.3 Test results

The abbreviations of the experimental indicators and corresponding definitions are listed in Table 5.

**Table 5:** Abbreviation of experiment indicators and definitions.

Abbreviation	Definition
***Gurobi_UB***	The upper bound of the MIP model obtained by the Gurobi solver in a preset running time.
***Gurobi_LB***	The best feasible objective value found by the Gurobi solver in a preset running time.
***VND_LB_Avg***	The average feasible objective value obtained by the ***VND*** after a preset number of iterations.
***VNS_LB_Avg***	The average feasible objective value obtained by the ***VNS*** after a preset number of iterations.
***MS_VND_LB_Avg***	The average feasible objective value obtained by the ***MS_VND*** after a preset number of iterations.
***MS_VNS_LB_Avg***	The average feasible objective value obtained by the ***MS_VNS*** after a preset number of iterations.
***VND_LB_Best***	The best feasible objective value obtained by the ***VND*** after a preset number of iterations.
***VNS_LB_Best***	The best feasible objective value obtained by the ***VNS*** after a preset number of iterations.
***MS_VND_LB_Best***	The best feasible objective value obtained by the ***MS_VND*** after a preset number of iterations.
***MS_VNS_LB_Best***	The best feasible objective value obtained by the ***MS_VNS*** after a preset number of iterations.
***Gurobi_Gap***	The gap between ***Gurobi_LB*** and ***Gurobi_UB***: (***Gurobi_UB****-****Gurobi_LB***)/***Gurobi_UB***.
***VND_Gap_Avg***	The gap between ***VND_LB_Avg*** and ***Gurobi_LB***: (***Gurobi_LB***-***VND_LB_Avg***)/***Gurobi_LB***.
***VNS_Gap_Avg***	The gap between ***VNS_LB_Avg*** and ***Gurobi_LB***: (***Gurobi_LB***-***VNS_LB_Avg***)/***Gurobi_LB***.
***MS_VND_Gap_Avg***	The gap between ***MS_VND_LB_Avg*** and ***Gurobi_LB***: (***Gurobi_LB***-***MS_VND_LB_Avg***)/***Gurobi_LB***.
***MS_VNS_Gap_Avg***	The gap between ***MS_VNS_LB_Avg*** and ***Gurobi_LB***: (***Gurobi_LB***-***MS_VNS_LB_Avg***)/***Gurobi_LB***.
***VND_Gap_Best***	The gap between ***VND_LB_Best*** and ***Gurobi_LB***: (***Gurobi_LB***-***VND_LB_Best***)/***Gurobi_LB***.
***VNS_Gap_Best***	The gap between ***VNS_LB_Best*** and ***Gurobi_LB***: (***Gurobi_LB***-***VNS_LB_Best***)/***Gurobi_LB***.
***MS_VND_Gap_Best***	The gap between ***MS_VND_LB_Best*** and ***Gurobi_LB***: (***Gurobi_LB***-***MS_VND_LB_Best***)/***Gurobi_LB***.
***MS_VNS_Gap_Best***	The gap between ***MS_VNS_LB_Best*** and ***Gurobi_LB***: (***Gurobi_LB***-***MS_VNS_LB_Best***)/***Gurobi_LB***.
***Gurobi_Time***	CPU time for solving the MIP model by the Gurobi solver (second).
***VND_Time***	Average CPU time for solving the MIP model by the ***VND*** (second).
***VNS_Time***	Average CPU time for solving the MIP model by the ***VNS*** (second).
***MS_VND_Time***	Average CPU time for solving the MIP model by the ***MS_VND*** (second).
***MS_VNS_Time***	Average CPU time for solving the MIP model by the ***MS_VNS*** (second).

Results solved by the Gurobi solver, the VND, the VNS, the MS_VND and the MS_VNS are shown in Table 6 (small size graphs), Table 7 (medium size graphs) and Table 8 (large size graphs). Each instance has been solved 10 times by each algorithm.

**Table 6:** Computational results for instances of small size graphs.

Instances	Pd-pairs	Vehicles	Gurobi			Time (second)	VND				Time (second)	VNS				Time (second)	MS_VND				Time (second)	MS_VNS				Time (second)
			*UB*	*LB*	*Gap*		*LB*		*Gap*			*LB*		*Gap*			*LB*		*Gap*			*LB*		*Gap*		
							*Avg*	*Best*	*Avg*	*Best*		*Avg*	*Best*	*Avg*	*Best*		*Avg*	*Best*	*Avg*	*Best*		*Avg*	*Best*	*Avg*	*Best*	
3-4-10-1-1-L	132	132	34216	32681	4.49%	57334	32928	33109	-0.76%	-1.31%	7	31081	33102	4.90%	-1.29%	8	33132	**33152**	-1.38%	-1.44%	48	30836	31517	5.65%	3.56%	267
3-4-10-1-1-H	132	132	136978	110919	19.02%	108565	134549	135887	-21.30%	-22.51%	9	130067	135881	-17.26%	-22.50%	7	135935	**135973**	-22.55%	-22.59%	51	135908	135932	-22.53%	-22.55%	373
3-4-10-1-3-L	132	44	34429	30121	12.51%	108686	32252	33918	-7.07%	-12.61%	7	28891	31630	4.08%	-5.01%	4	33751	**33928**	-12.05%	-12.64%	64	30738	31828	-2.05%	-5.67%	231
3-4-10-1-3-H	132	44	154606	151500	2.01%	52132	127017	134431	16.16%	11.27%	9	124213	135222	18.01%	10.74%	5	154210	**154240**	-1.79%	-1.81%	53	128448	136410	15.22%	9.96%	282
3-4-10-1-10-L	132	14	34072	32946	3.30%	96339	31787	32034	3.52%	2.77%	7	31567	32781	4.19%	0.50%	7	31802	**32895**	3.47%	0.15%	45	32619	32776	0.99%	0.52%	396
3-4-10-1-10-H	132	14	136215	134367	1.36%	104729	128286	129385	4.53%	3.71%	6	127461	132468	5.14%	1.41%	5	131817	**133199**	1.90%	0.87%	59	126228	130829	6.06%	2.63%	363
3-4-10-3-1-L	42	42	9918	9918	0.00%	2214	9903	**9905**	0.15%	0.13%	4	9900	9900	0.18%	0.18%	2	9905	**9905**	0.13%	0.13%	14	9903	**9905**	0.15%	0.13%	95
3-4-10-3-1-H	42	42	51214	51214	0.00%	718	51190	51192	0.05%	0.04%	4	51190	51206	0.05%	0.02%	3	51214	**51214**	0.00%	0.00%	30	51210	**51214**	0.01%	0.00%	106
3-4-10-3-3-L	47	16	9945	9945	0.00%	39032	9913	9913	0.32%	0.32%	4	9923	**9942**	0.22%	0.03%	3	9942	**9942**	0.03%	0.03%	26	9913	9913	0.32%	0.32%	95
3-4-10-3-3-H	43	15	49033	49033	0.00%	93	49028	**49031**	0.01%	0.00%	5	49018	49026	0.03%	0.01%	2	49028	**49031**	0.01%	0.00%	18	49029	**49031**	0.01%	0.00%	76
3-4-10-3-10-L	42	5	6546	6546	0.00%	7	6447	**6546**	1.51%	0.00%	5	6336	6398	3.21%	2.26%	2	6529	**6546**	0.26%	0.00%	25	6544	**6546**	0.03%	0.00%	90
3-4-10-3-10-H	48	5	34863	34863	0.00%	16	32771	33312	6.00%	4.45%	4	32246	32246	7.51%	7.51%	2	33410	**33799**	4.17%	3.05%	21	32246	32246	7.51%	7.51%	99
3-4-10-5-1-L	25	25	5610	5610	0.00%	11	5563	**5610**	0.84%	0.00%	3	5546	5548	1.14%	1.11%	2	5608	**5610**	0.04%	0.00%	22	5545	5548	1.16%	1.11%	49
3-4-10-5-1-H	23	23	22563	22563	0.00%	15	22563	**22563**	0.00%	0.00%	3	22563	**22563**	0.00%	0.00%	2	22563	**22563**	0.00%	0.00%	11	22563	**22563**	0.00%	0.00%	46
3-4-10-5-3-L	33	11	8405	8405	0.00%	8	8252	8351	1.82%	0.64%	3	8252	8351	1.82%	0.64%	1	8258	**8405**	1.75%	0.00%	20	8214	8238	2.27%	1.99%	50
3-4-10-5-3-H	30	10	30603	30603	0.00%	6	30595	**30598**	0.03%	0.02%	3	30514	30575	0.29%	0.09%	1	30596	**30598**	0.02%	0.02%	14	30598	**30598**	0.02%	0.02%	55
3-4-10-5-10-L	28	3	3274	3274	0.00%	1	3274	**3274**	0.00%	0.00%	3	3192	**3274**	2.50%	0.00%	2	3274	**3274**	0.00%	0.00%	14	3274	**3274**	0.00%	0.00%	54
3-4-10-5-10-H	24	3	11372	11372	0.00%	2	11250	**11372**	1.07%	0.00%	3	11340	11340	0.28%	0.28%	2	11366	**11372**	0.05%	0.00%	13	11213	11340	1.40%	0.28%	41
3-4-10-10-1-L	12	12	2184	2184	0.00%	1	2184	**2184**	0.00%	0.00%	2	2184	**2184**	0.00%	0.00%	1	2184	**2184**	0.00%	0.00%	5	2184	**2184**	0.00%	0.00%	17
3-4-10-10-1-H	13	13	13907	13907	0.00%	2	13907	**13907**	0.00%	0.00%	2	13907	**13907**	0.00%	0.00%	1	13907	**13907**	0.00%	0.00%	7	13907	**13907**	0.00%	0.00%	24
3-4-10-10-3-L	17	6	3026	3026	0.00%	1	2774	2774	8.33%	8.33%	2	2774	2774	8.33%	8.33%	2	2921	**3026**	3.47%	0.00%	14	2816	2899	6.94%	4.20%	33
3-4-10-10-3-H	13	3	12840	12840	0.00%	1	12431	**12840**	3.19%	0.00%	1	12226	12226	4.78%	4.78%	1	12533	**12840**	2.39%	0.00%	7	12840	**12840**	0.00%	0.00%	20
3-4-10-10-10-L	13	2	820	820	0.00%	1	795	**820**	3.05%	0.00%	1	789	811	3.78%	1.10%	2	817	**820**	0.37%	0.00%	14	811	811	1.10%	1.10%	31
3-4-10-10-10-H	14	2	5607	5607	0.00%	1	5420	5420	3.34%	3.34%	2	5482	**5607**	2.23%	0.00%	1	5607	**5607**	0.00%	0.00%	11	5545	**5607**	1.11%	0.00%	19
Average			33844	32261	1.78%	23746	31878	32432	1.03%	-0.06%	4	31278	32457	2.31%	0.42%	3	33346	**33501**	-0.82%	-1.43%	25	31797	32415	1.06%	0.21%	121

Note: The best feasible objective values found by the Heuristic Approaches in this paper is indicated in boldface. The indexes in this table are introduced in Table 5

**Table 7:** Computational results for instances of medium size graphs.

Instances	Pd-pairs	Vehicles	Gurobi			Time (second)	VND				Time (second)	VNS				Time (second)	MS_VND				Time (second)	MS_VNS				Time (second)
			*UB*	*LB*	*Gap*		*LB*		*Gap*			*LB*		*Gap*			*LB*		*Gap*			*LB*		*Gap*		
							*Avg*	*Best*	*Avg*	*Best*		*Avg*	*Best*	*Avg*	*Best*		*Avg*	*Best*	*Avg*	*Best*		*Avg*	*Best*	*Avg*	*Best*	
6-8-10-10-1-L	235	235	-	-	-	-	116833	116865	-	-	13	116891	116914	-	-	14	116983	**117008**	-	-	-	117005	117024	-	-	491
6-8-10-10-1-H	236	236	-	-	-	-	516404	516437	-	-	26	516507	516537	-	-	15	516535	**516582**	-	-	-	516517	516579	-	-	687
6-8-10-10-3-L	225	75	104232	67284	35.45%	108326	100221	100307	-48.95%	-49.08%	17	96598	96856	-43.57%	-43.95%	10	100432	**100513**	-49.27%	-49.39%	41	99254	100446	-47.52%	-49.29%	562
6-8-10-10-3-H	226	76	473459	373627	21.09%	108944	466957	468647	-24.98%	-25.43%	16	467052	467072	-25.00%	-25.01%	7	468728	**468760**	-25.45%	-25.46%	39	468757	468772	-25.46%	-25.47%	632
6-8-10-10-10-L	217	22	78211	60219	23.00%	108178	60717	61050	-0.83%	-1.38%	26	60625	60684	-0.67%	-0.77%	10	61149	**61701**	-1.54%	-2.46%	33	60961	61082	-1.23%	-1.43%	745
6-8-10-10-10-H	258	26	338092	263477	22.07%	108796	279376	284043	-6.03%	-7.81%	27	274997	279218	-4.37%	-5.97%	15	293349	**297011**	-11.34%	-12.73%	46	286728	296510	-8.82%	-12.54%	1085
6-8-10-25-1-L	94	94	49933	49933	0.00%	39349	49862	49885	0.14%	0.10%	13	49829	49897	0.21%	0.07%	5	49885	49892	0.10%	0.08%	18	49898	**49902**	0.07%	0.06%	461
6-8-10-25-1-H	92	92	197280	197280	0.00%	33588	197255	**197264**	0.01%	0.01%	11	197264	**197264**	0.01%	0.01%	4	197264	**197264**	0.01%	0.01%	17	197264	**197264**	0.01%	0.01%	377
6-8-10-25-3-L	101	34	46332	46332	0.00%	29776	45883	45889	0.97%	0.96%	15	45879	45881	0.98%	0.97%	5	45887	45889	0.96%	0.96%	20	45934	**46040**	0.86%	0.63%	376
6-8-10-25-3-H	90	30	166326	166308	0.01%	38317	161644	161901	2.80%	2.65%	19	161508	161901	2.89%	2.65%	4	162355	**163673**	2.38%	1.58%	23	161901	161901	2.65%	2.65%	481
6-8-10-25-10-L	96	10	21552	21552	0.00%	2224	21370	21487	0.84%	0.30%	18	21487	21487	0.30%	0.30%	8	21458	21499	0.44%	0.25%	18	21503	**21534**	0.23%	0.08%	489
6-8-10-25-10-H	90	9	73877	73614	0.36%	10146	71004	**72560**	3.55%	1.43%	25	68163	68862	7.40%	6.46%	8	71712	**72560**	2.58%	1.43%	35	71621	72003	2.71%	2.19%	653
6-8-10-50-1-L	42	42	16469	16469	0.00%	99	16357	**16424**	0.68%	0.27%	15	16350	16404	0.72%	0.39%	2	16416	16416	0.32%	0.32%	24	16400	16416	0.42%	0.32%	76
6-8-10-50-1-H	44	44	79372	79372	0.00%	51	79228	79288	0.18%	0.11%	13	79265	79299	0.13%	0.09%	4	79327	**79348**	0.06%	0.03%	26	79259	79259	0.14%	0.14%	244
6-8-10-50-3-L	52	18	19935	19935	0.00%	193	19415	19421	2.61%	2.58%	11	19421	19421	2.58%	2.58%	7	19495	**19689**	2.21%	1.23%	20	19454	19521	2.41%	2.08%	241
6-8-10-50-3-H	33	11	51849	51849	0.00%	11	51267	51790	1.12%	0.11%	9	51005	51005	1.63%	1.63%	2	51204	**51849**	1.24%	0.00%	13	51025	51064	1.59%	1.51%	139
6-8-10-50-10-L	44	5	6183	6183	0.00%	6	6016	**6182**	2.70%	0.02%	9	6099	**6182**	1.36%	0.02%	2	6182	**6182**	0.02%	0.02%	14	6182	**6182**	0.02%	0.02%	240
6-8-10-50-10-H	30	3	17691	17691	0.00%	5	17691	**17691**	0.00%	0.00%	9	17691	**17691**	0.00%	0.00%	2	17691	**17691**	0.00%	0.00%	9	17691	**17691**	0.00%	0.00%	189
6-8-10-100-1-L	19	19	8589	8589	0.00%	4	8589	**8589**	0.00%	0.00%	6	8589	**8589**	0.00%	0.00%	1	8589	**8589**	0.00%	0.00%	6	8589	**8589**	0.00%	0.00%	49
6-8-10-100-1-H	20	20	44893	44893	0.00%	4	44893	**44893**	0.00%	0.00%	6	44893	**44893**	0.00%	0.00%	1	44893	**44893**	0.00%	0.00%	6	44893	**44893**	0.00%	0.00%	28
6-8-10-100-3-L	27	9	9247	9247	0.00%	5	9240	**9247**	0.08%	0.00%	4	9114	9237	1.44%	0.11%	2	9237	9237	0.11%	0.11%	12	9237	9237	0.11%	0.11%	119
6-8-10-100-3-H	22	8	33852	33852	0.00%	3	33841	**33852**	0.03%	0.00%	2	33771	33818	0.24%	0.10%	1	33852	**33852**	0.00%	0.00%	10	33852	**33852**	0.00%	0.00%	139
6-8-10-100-10-L	25	3	2781	2781	0.00%	3	2734	**2734**	1.69%	1.69%	2	2734	**2734**	1.69%	1.69%	1	2734	**2734**	1.69%	1.69%	8	2734	**2734**	1.69%	1.69%	101
6-8-10-100-10-H	18	2	9165	9165	0.00%	2	9165	**9165**	0.00%	0.00%	2	9165	**9165**	0.00%	0.00%	1	9165	**9165**	0.00%	0.00%	6	9165	**9165**	0.00%	0.00%	50
6-8-10-200-1-L	14	14	6250	6250	0.00%	3	6236	6241	0.22%	0.14%	2	6232	6232	0.29%	0.29%	1	6242	**6250**	0.13%	0.00%	9	6232	6232	0.29%	0.29%	45
Average			68620	60417	3.64%	21003	65157	65508	-2.12%	-2.52%	10	64752	64969	-1.70%	-1.93%	4	65816	**66085**	-2.58%	-2.84%	17	65507	65927	-2.39%	-2.65%	281

Note: The best feasible objective values found by the Heuristic Approaches in this paper is indicated in boldface. The indexes in this table are introduced in Table 5.

**Table 8:** Computational results for instances of large size graphs.

Instances	Pd-pairs	Vehicles	Gurobi			Time (second)	VND				Time (second)	VNS				Time (second)	MS_VND				Time (second)	MS_VNS				Time (second)
			*UB*	*LB*	*Gap*		*LB*		*Gap*			*LB*		*Gap*			*LB*		*Gap*			*LB*		*Gap*		
							*Avg*	*Best*	*Avg*	*Best*		*Avg*	*Best*	*Avg*	*Best*		*Avg*	*Best*	*Avg*	*Best*		*Avg*	*Best*	*Avg*	*Best*	
10-10-10-50-1-L	188	188	-	-	-	-	131474	131521	-	-	11	131586	131619	-	-	50	131635	**131703**	-	-	73	131600	131610	-	-	210
10-10-10-50-1-H	199	199	-	-	-	-	603526	603650	-	-	15	603728	603802	-	-	39	603842	**603900**	-	-	74	603775	603827	-	-	284
10-10-10-50-3-L	222	74	-	-	-	-	130314	131933	-	-	29	130665	131811	-	-	77	131860	**133212**	-	-	152	131714	131901	-	-	1063
10-10-10-50-3-H	203	68	-	-	-	-	539915	541851	-	-	16	544218	546792	-	-	69	545060	547189	-	-	71	546634	**548831**	-	-	977
10-10-10-50-10-L	186	19	59967	56465	5.84%	108527	53118	54924	5.93%	2.73%	28	52816	53080	6.46%	5.99%	61	54623	**55662**	3.26%	1.42%	116	54145	55339	4.11%	1.99%	1327
10-10-10-50-10-H	203	21	344505	249154	27.68%	108420	230001	231733	7.69%	6.99%	28	234325	235742	5.95%	5.38%	97	233858	239642	6.14%	3.82%	59	237027	**241101**	4.87%	3.23%	1750
10-10-10-100-1-L	110	110	77092	75443	2.14%	108457	75436	75455	0.01%	-0.02%	23	75421	75447	0.03%	-0.01%	27	75441	75447	0.00%	-0.01%	36	75452	**75458**	-0.01%	-0.02%	480
10-10-10-100-1-H	87	87	280912	280912	0.00%	11413	280787	280791	0.04%	0.04%	20	280769	280786	0.05%	0.04%	31	280828	**280898**	0.03%	0.00%	43	280805	280817	0.04%	0.03%	728
10-10-10-100-3-L	105	31	62249	61238	1.62%	70048	59433	59561	2.95%	2.74%	12	59400	59409	3.00%	2.99%	20	59910	**60408**	2.17%	1.36%	64	59432	59444	2.95%	2.93%	460
10-10-10-100-3-H	87	29	219504	212445	3.22%	108757	211073	211698	0.65%	0.35%	27	209740	211570	1.27%	0.41%	21	211848	**213138**	0.28%	-0.33%	30	211582	211588	0.41%	0.40%	680
10-10-10-100-10-L	113	12	26582	25394	4.47%	108226	23305	23627	8.23%	6.96%	6	23992	24553	5.52%	3.31%	89	24644	**24996**	2.95%	1.57%	67	24772	**24996**	2.45%	1.57%	604
10-10-10-100-10-H	76	8	66552	66552	0.00%	222	65458	65458	1.64%	1.64%	3	65715	66229	1.26%	0.49%	40	66431	**66552**	0.18%	0.00%	42	66404	**66552**	0.22%	0.00%	105
10-10-10-200-1-L	44	44	30204	30204	0.00%	64	30152	**30181**	0.17%	0.08%	4	30121	30121	0.27%	0.27%	19	30153	30166	0.17%	0.13%	35	30141	**30181**	0.21%	0.08%	114
10-10-10-200-1-H	49	49	139537	139537	0.00%	90	139466	139514	0.05%	0.02%	4	139505	139514	0.02%	0.02%	40	139515	**139528**	0.02%	0.01%	20	139528	**139528**	0.01%	0.01%	62
10-10-10-200-3-L	45	15	22980	22980	0.00%	22	22269	22281	3.09%	3.04%	4	22150	22281	3.61%	3.04%	19	22753	**22819**	0.99%	0.70%	31	22602	22651	1.64%	1.43%	60
10-10-10-200-3-H	60	20	139836	134403	3.89%	81579	127257	130248	5.32%	3.09%	6	125947	127112	6.29%	5.42%	36	130157	**131375**	3.16%	2.25%	58	125983	126554	6.26%	5.84%	80
10-10-10-200-10-L	54	6	11920	11920	0.00%	10	11920	**11920**	0.00%	0.00%	4	11920	**11920**	0.00%	0.00%	51	11920	**11920**	0.00%	0.00%	25	11920	**11920**	0.00%	0.00%	111
10-10-10-200-10-H	64	7	60260	60260	0.00%	24	57697	57830	4.25%	4.03%	4	57830	57830	4.03%	4.03%	31	58073	**60260**	3.63%	0.00%	23	57830	57830	4.03%	4.03%	98
10-10-10-500-1-L	20	20	12331	12331	0.00%	4	12301	12303	0.24%	0.23%	2	12284	12300	0.38%	0.25%	13	12302	**12310**	0.24%	0.17%	16	12300	12300	0.25%	0.25%	36
10-10-10-500-1-H	13	13	42093	42093	0.00%	2	42093	**42093**	0.00%	0.00%	1	42093	**42093**	0.00%	0.00%	22	42093	**42093**	0.00%	0.00%	6	42093	**42093**	0.00%	0.00%	18
10-10-10-500-3-L	13	5	32671	32671	0.00%	1	32671	**32671**	0.00%	0.00%	1	32671	**32671**	0.00%	0.00%	13	32671	**32671**	0.00%	0.00%	7	32671	**32671**	0.00%	0.00%	19
10-10-10-500-3-H	19	7	36724	36724	0.00%	2	36415	36609	0.84%	0.31%	2	36318	36318	1.11%	1.11%	19	36562	**36724**	0.44%	0.00%	13	36512	36609	0.58%	0.31%	35
10-10-10-500-10-L	18	2	3366	3366	0.00%	1	3366	**3366**	0.00%	0.00%	3	3366	**3366**	0.00%	0.00%	16	3366	**3366**	0.00%	0.00%	8	3366	**3366**	0.00%	0.00%	28
10-10-10-500-10-H	22	3	20093	20093	0.00%	2	20046	20093	0.23%	0.00%	2	19951	19951	0.71%	0.71%	13	20093	**20093**	0.00%	0.00%	13	19951	19951	0.71%	0.71%	40
10-10-10-1000-1-L	12	12	7392	7392	0.00%	2	7392	**7392**	0.01%	0.01%	1	7392	**7392**	0.00%	0.00%	13	7392	**7392**	0.00%	0.00%	3	7392	**7392**	0.00%	0.00%	11
Average			67560	63129	1.88%	27149	61594	61905	1.59%	1.24%	8	61673	61903	1.54%	1.29%	30	62093	**62586**	0.91%	0.43%	28	61988	62236	1.11%	0.88%	266

Note: The best feasible objective values found by the Heuristic Approaches in this paper is indicated in boldface. The indexes in this table are introduced in Table 5.

**Fig 22:**
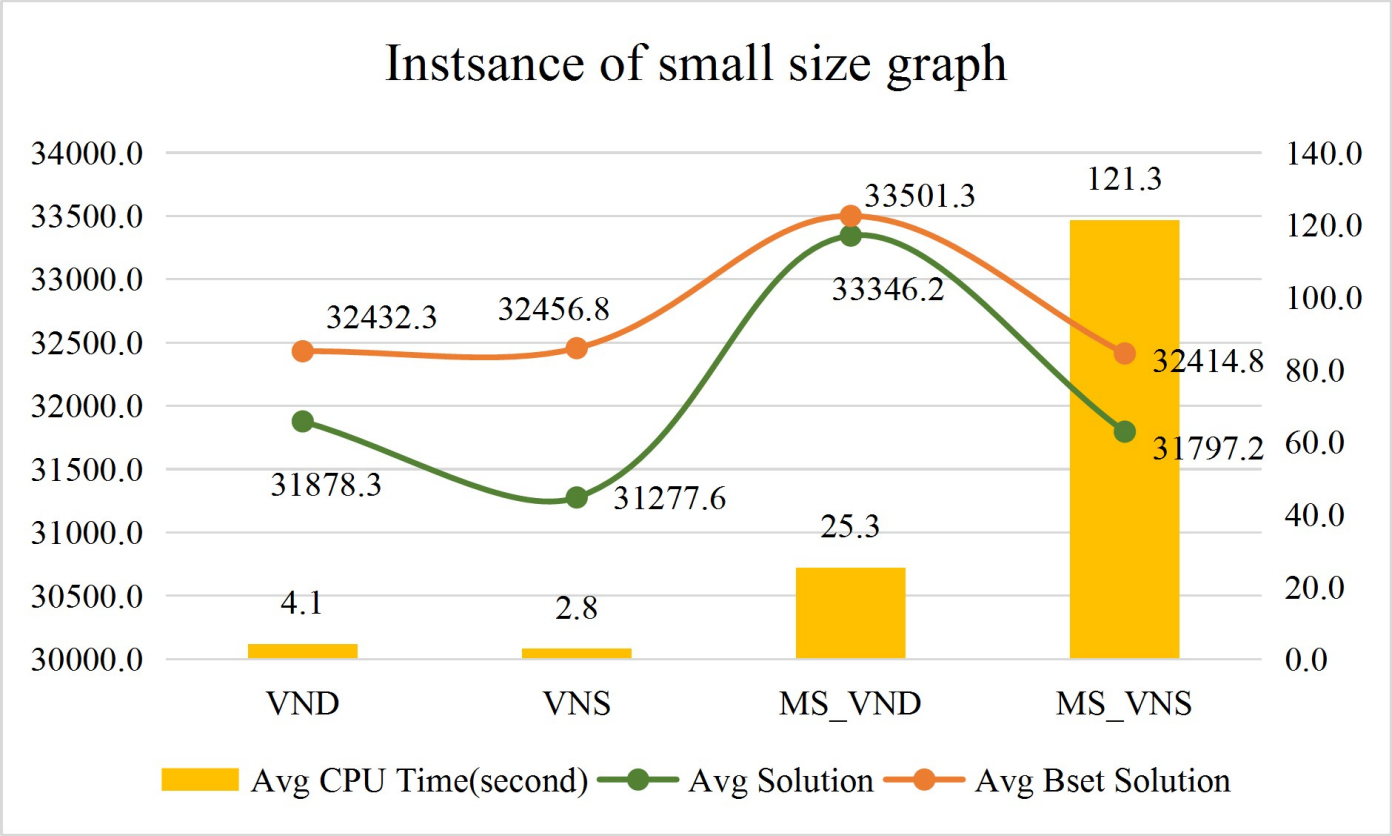
Performance of the approaches for instances of small size graphs.

**Fig 23:**
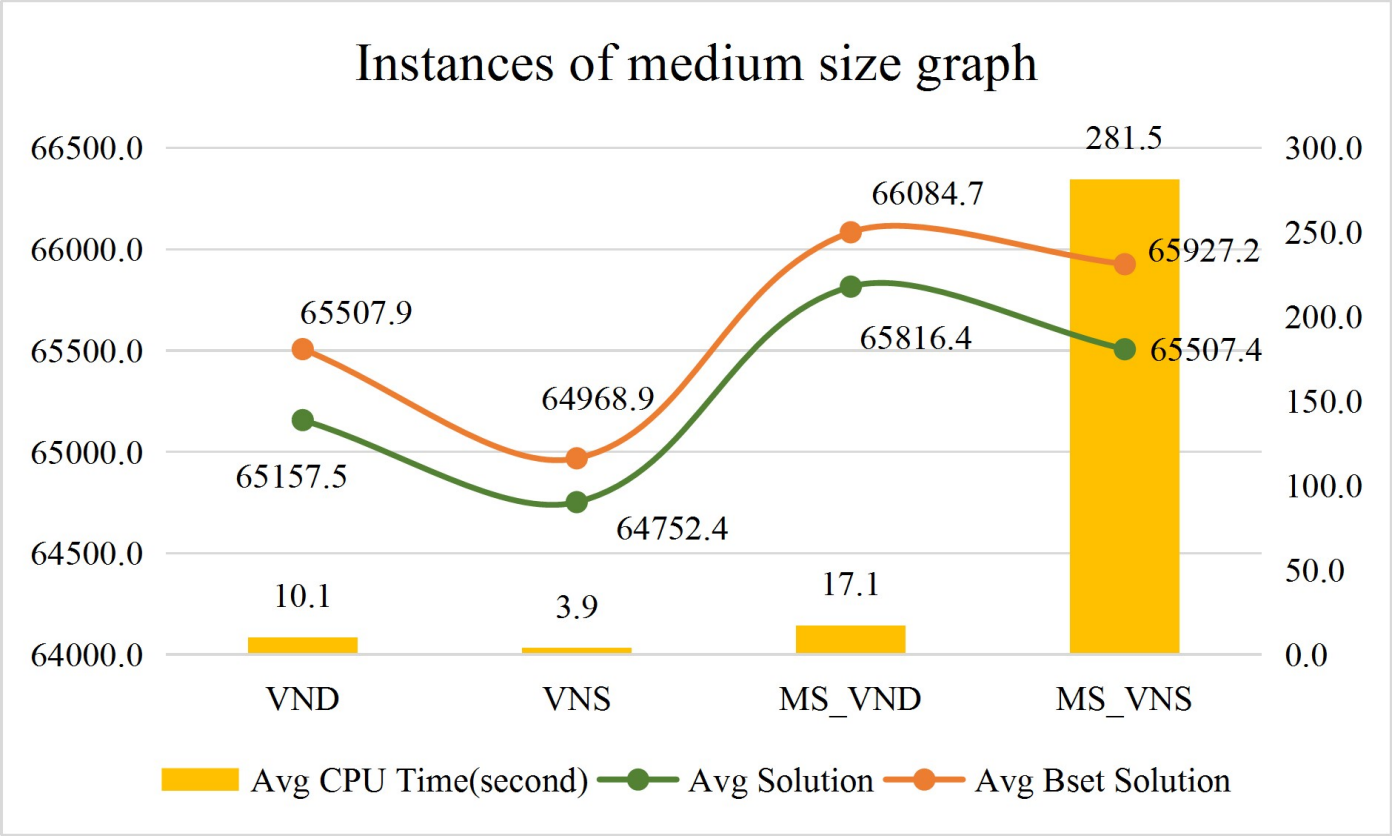
Performance of the approaches for instances of medium size graphs.

**Fig 24:**
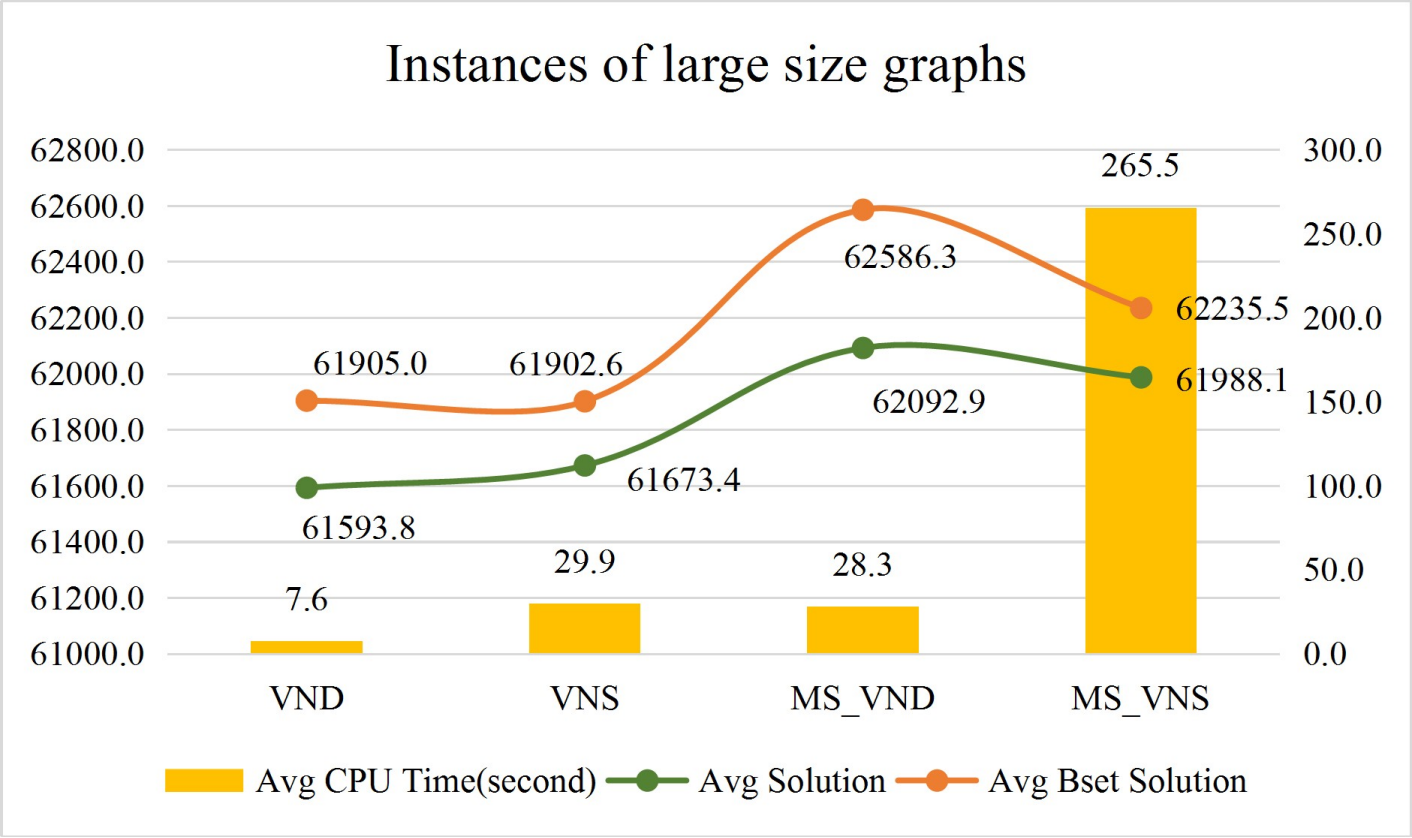
Performance of the approaches for instances of large size graphs.

Comparison of the average calculation efficiency of the VND, the VNS, the MS_VND and the MS_VNS are shown in Figs 22–24.

The above results shows that:

1. CPU time of the Gurobi solver, the VND, the VNS, the MS_VND and the MS_VNS are independent of road network scale. Numbers of pd-pairs and vehicles are the major influential factors to the CPU time of the Gurobi solver, numbers of pd-pairs are the major influential factors to the CPU time of the VND, the VNS, the MS_VND and the MS_VNS.
2. In general, instances with no more than 100 pd-pairs can get an optimum solution by the Gurobi solver in preset termination CPU time (108000 seconds). Instances with more than 5000 (pd-pairs×vehicles) always cannot receive the optimal solution with no more than 20% *Gap_Gurobi* within an acceptable CPU time (108000 seconds) by Gurobi solver. Large instances with over 40000 (pd-pairs×vehicles) always cannot get feasible solutions within 108,000 seconds by the Gurobi solver.
3. In almost all instances, the VND, the VNS, the MS_VND and the MS_VNS with the operators proposed in this paper always can acquire the optimal solution with no more than 10% *Gap* to the Gurobi solver within an acceptable CPU time. The MS_VND can acquire the optimal solution with no more than 5% *Gap* to the Gurobi solver within an acceptable CPU time. The MS_VNS always takes a little more CPU time than the MS_VND to solve the instances with more pd-pairs.
4. The VND, the VNS, the MS_VND and the MS_VNS can even get better solutions than the Gurobi solver for the instances with large numbers of pd-pairs and vehicles.
5. In almost all instances, the MS_VND significantly outperforms the VND, the VNS and the MS_VNS in terms of average solution quality (Figs 22–24). The MS_VND acquires almost all the best average solutions and most of the best solution for each instance (boldface numerical value in Table 6, Table 7 and Table 8).

## 6 Conclusions

A new Pickup and Delivery Problem with new route structure, the OPDPSTRP, which is proposed in real-life connected graphs, is introduced and formulated in a new way. Five operators are proposed for the OPDPSTRP. A basic VND, a basic VND, a new MS_VND, and a new MS_VNS are developed for this problems and compared with the Gurobi solver. The test results show that the VND, the VNS, the MS_VND and the MS_VNS always can acquire the optimal solution with no more than 10% *Gap* to the Gurobi solver within an acceptable CPU time. In almost all instances, the MS_VND significantly outperforms the VND the VNS and the MS_VNS in terms of solution quality. The MS_VNS always takes a little more CPU time than the MS_VND to solve the instances with more pd-pairs. In the large instances, the MS_VND is still able to generate good feasible solutions in a reasonable CPU time, which is of vital practical significance for real-life instances.

Our future work will concentrate on three aspects: (1) In order to improve the algorithm efficiency of the OPDPSTRP, path feasible strategy for neighborhoods will be studied. (2) There is only one shortest path between any two nodes in the connected graph in this paper, but there may be more than one shortest path between two nodes in some other real-life road networks, which we are going to study in the future. (3) The OPDPSTRP with time windows will be studied in the future.

## Supporting information

S1 Appendix Values of certain relative notations for the routes combined with two pd-pairs/vehicles. (DOC)

S2 Appendix Proof of Theorem 1, Lemma 1, Lemma 2, Lemma 3. (DOC)

S3 Appendix Instances and relative research. (DOC)

S4 Appendix Appendix Parameter setting for the VND, the VNS, the MS_VND and the MS_VNS. (DOC)
