# Supplementary material for: Multi-start heuristic approaches for one-to-one pickup and delivery problems with shortest-path transport along real-life paths
Source: PLoS One. 2020 Feb 6;15(2):e0227702. doi: 10.1371/journal.pone.0227702 (PMC7004362; doi:10.1371/journal.pone.0227702)
Supplement: S1 Appendix — (DOC) [file pone.0227702.s001.doc]

**S1 Appendix. Values of certain relative notations for the routes combined with two pd-pairs/vehicle.**

|  | $lc_{i,j}^{l}$ | $lc_{j,i}^{l}$ | $connect\_to\_judge_{i,j}^{l}$ | $connect\_to\_judge_{j,i}^{l}$ | $connect\_after\_judge_{i,j}^{l}$ | $connect\_after\_judge_{j,i}^{l}$ |
| --- | --- | --- | --- | --- | --- | --- |
|  | *0* | $\infty$ | *1* | *0* | *0* | *0* |
|  | *0* | *0* | *1* | *1* | *0* | *1* |
|  | *0* | $\infty$ | *1* | *0* | *1* | *0* |
|  | *0* | *0* | *1* | *1* | *1* | *1* |
|  | $\infty$ | $\infty$ | *0* | *0* | *0* | *0* |
|  | $\infty$ | $\infty$ | *0* | *0* | *0* | *0* |
|  | $\infty$ | $\infty$ | *0* | *0* | *0* | *0* |
|  | $\infty$ | $\infty$ | *0* | *0* | *0* | *0* |
|  | *0* | *0* | *1* | *1* | *1* | *1* |
|  | $\infty$ | $\infty$ | *0* | *0* | *0* | *0* |
|  | $\infty$ | *0* | *0* | *1* | *0* | *1* |
|  | $\infty$ | $\infty$ | *0* | *0* | *0* | *0* |
|  | $\infty$ | $\infty$ | *0* | *0* | *0* | *0* |
|  | *0* | $\infty$ | *1* | *0* | *1* | *0* |
|  | *L1* | $\infty$ | *1* | *0* | *1* | *0* |
|  | *0* | $\infty$ | *1* | *0* | *1* | *0* |
|  | $\infty$ | $\infty$ | *0* | *0* | *0* | *0* |
|  | $\infty$ | $\infty$ | *0* | *0* | *0* | *0* |
|  | $\infty$ | $\infty$ | *0* | *0* | *0* | *0* |
|  | $\infty$ | $\infty$ | *0* | *0* | *0* | *0* |
|  | $\infty$ | $\infty$ | *0* | *0* | *0* | *0* |
|  | $\infty$ | $\infty$ | *0* | *0* | *0* | *0* |
|  | $\infty$ | $\infty$ | *0* | *0* | *0* | *0* |
|  | $\infty$ | $\infty$ | *0* | *0* | *0* | *0* |
|  | *L1* | *L2* | *1* | *1* | *1* | *1* |
|  | $\infty$ | $\infty$ | *0* | *0* | *0* | *0* |
|  | $\infty$ | $\infty$ | *0* | *0* | *0* | *0* |
|  | *L1* | $\infty$ | *1* | *0* | *1* | *0* |
|  | *0* | $\infty$ | *1* | *0* | *1* | *0* |
|  | *L1* | $\infty$ | *1* | *0* | *1* | *0* |
