# Supplementary material for: Multi-start heuristic approaches for one-to-one pickup and delivery problems with shortest-path transport along real-life paths
Source: PLoS One. 2020 Feb 6;15(2):e0227702. doi: 10.1371/journal.pone.0227702 (PMC7004362; doi:10.1371/journal.pone.0227702)
Supplement: S2 Appendix — (DOC) [file pone.0227702.s002.doc]

**S2 Appendix. Proof of Theorem 1, Lemma 1, Lemma 2, Lemma 3.**

1. **Proof of Theorem 1**

**Proof:**

- Sufficiency

If pd-pair *k* can be combined with all pd-pairs transported by route *m*, then the feasibility of inserting pd-pair *k* into route *m* can be proved using three kinds of cases mentioned in Section 4.2.

**Case 1:** both *r_i_* and *r_i_* can be found

As $l_{r_{i},r_{i+1}}=l_{r_{i},p_{k}}+l_{p_{k},r_{i+1}}$ and $l_{r_{j},r_{j+1}}=l_{r_{j},d_{k}}+l_{d_{k},r_{j+1}}$, so $r_{i}-p_{k}-r_{i+1}$ and $r_{j}-d_{k}-r_{j+1}$ are both the shortest paths, the structure of new route *m’* generated by inserting pd-pair *k* into route *m* can be separated into two categories: $R'_{m}(r_{1}...r_{i}-p_{k}-r_{i+1}...r_{j}-d_{k}-r_{j+1}...r_{n})$ and $R'_{m}(r_{1}...r_{i}-p_{k}-d_{k}-r_{i+1}...r_{n})$.

1. If the new structure of route *m’* is $R'_{m}(r_{1}...r_{i}-p_{k}-r_{i+1}...r_{j}-d_{k}-r_{j+1}...r_{n})$, namely there are no less than one point between *p_k_* and *d_k_*. Let *Z_1_*=$\left\{r_{1},...,r_{i}\right\}$, *Z_2_*=$\left\{r_{\text{i}+1},...,r_{j}\right\}$, and *Z_3_*=$\left\{r_{\text{j}+1},...,r_{n}\right\}$. Obviously each pd-pair in route *m* can also be transported by$R'_{m}(r_{1}...r_{i}-p_{k}-r_{i+1}...r_{j}-d_{k}-r_{j+1}...r_{n})$since $l_{r_{i},r_{i+1}}=l_{r_{i},p_{k}}+l_{p_{k},r_{i+1}}$ and $l_{r_{j},r_{j+1}}=l_{r_{j},d_{k}}+l_{d_{k},r_{j+1}}$. As for any pd-pair *r_s_-r_t_* they can be combined with pd-pair *k*:

If $\text{r}_{\text{s}}\in\text{Z}_{1}$, $r_{t}\in Z_{2}$, then *r_t_* is in the unique shortest path $p_{k}-r_{t}-d_{k}$;

If $\text{r}_{\text{s}}\in\text{Z}_{2}$, $r_{t}\in Z_{2}$, then *r_s_* and *r_t_* are both in the unique shortest path $p_{k}-r_{\text{s}}-r_{t}-d_{k}$;

If $\text{r}_{\text{s}}\in\text{Z}_{2}$, $r_{t}\in Z_{3}$, then *r_s_* is in the unique shortest path $p_{k}-r_{\text{s}}-d_{k}$.

Above all, all pd-pairs including pd-pair *k* can be transported in $R'_{m}(r_{1}...r_{i}-p_{k}-r_{i+1}...r_{j}-d_{k}-r_{j+1}...r_{n})$, so route *m’* is RSF (**Definition 1** in Section 3.4), namely pd-pair *k* can be inserted into route *m* (**Definition 2** in Section 3.4).

1. If the new structure of route *m’* is $R'_{m}(r_{1}...r_{i}-p_{k}-d_{k}-r_{i+1}...r_{n})$, all pd-pairs including *k* can be transported in $R'_{m}(r_{1}...r_{i}-p_{k}-r_{i+1}...r_{j}-d_{k}-r_{j+1}...r_{n})$obviously, so route *m’* is RSF (**Definition 1** in Section 3.4), namely pd-pair *k* can be inserted into route *m* (**Definition 2** in Section 3.4).

**Case 2:** Only one of *r_i_* and *r_j_* can be found

The structure of new route *m’* generated by inserting pd-pair *k* into route *m* can be separated into two categories: $R'_{m}(r_{1}...r_{i}-p_{k}-r_{i+1}...r_{n}-d_{k})$ and $R'_{m}(p_{k}-r_{1}...r_{\text{j}}-d_{k}-r_{\text{j}+1}...r_{n})$.

1. If the new structure of route *m’* is $R'_{m}(r_{1}...r_{i}-p_{k}-r_{i+1}...r_{n}-d_{k})$, namely there are no less than one points between *p_k_* and *d_k_*. Let *Z_1_*=$\left\{r_{1},...,r_{i}\right\}$ and *Z_2_*=$\left\{r_{\text{i}+1},...,r_{\text{n}}\right\}$. Obviously each pd-pair in route *m* is can also be transported by$R'_{m}(r_{1}...r_{i}-p_{k}-r_{i+1}...r_{n}-d_{k})$since $l_{r_{i},r_{i+1}}=l_{r_{i},p_{k}}+l_{p_{k},r_{i+1}}$. As for any pd-pair $r_{\text{s}}-r_{t}$ these can be combined with pd-pair *k*:

If $\text{r}_{\text{s}}\in\text{Z}_{1}$, $r_{t}\in Z_{2}$, then *r_t_* is in the unique shortest path $p_{k}-r_{t}-d_{k}$;

If $\text{r}_{\text{s}}\in\text{Z}_{2}$, $r_{t}\in Z_{2}$, then *r_s_* and *r_t_* are both in the unique shortest path $p_{k}-r_{\text{s}}-r_{t}-d_{k}$.

Above all, all pd-pairs including pd-pair *k* can be transported in $R'_{m}(r_{1}...r_{i}-p_{k}-r_{i+1}...r_{n}-d_{k})$, so route *m’* is RSF (**Definition 1** in Section 3.4), namely pd-pair *k* can be inserted into route *m* (**Definition 2** in Section 3.4).

1. If the new structure of route *m’* is $R'_{m}(p_{k}-r_{1}...r_{\text{j}}-d_{k}-r_{\text{j}+1}...r_{n})$, namely there are no less than one points between *p_k_* and *d_k_*. Let *Z_1_*=$\left\{r_{1},...,r_{\text{j}}\right\}$ and *Z_2_*=$\left\{r_{\text{j}+1},...,r_{\text{n}}\right\}$. Obviously each pd-pair in route *m* is can also be transported by$R'_{m}(p_{k}-r_{1}...r_{\text{j}}-d_{k}-r_{\text{j}+1}...r_{n})$since $l_{r_{j},r_{j+1}}=l_{r_{j},d_{k}}+l_{d_{k},r_{j+1}}$. As for any pd-pair $r_{\text{s}}-r_{t}$ these can be combined with pd-pair *k*:

If $\text{r}_{\text{s}}\in\text{Z}_{1}$, $r_{t}\in Z_{2}$, then *r_t_* is in the unique shortest path $p_{k}-r_{t}-d_{k}$;

If $\text{r}_{\text{s}}\in\text{Z}_{1}$, $\text{r}_{\text{t}}\in\text{Z}_{1}$, then *r_s_* and *r_t_* are both in the unique shortest path $p_{k}-r_{\text{s}}-r_{t}-d_{k}$.

Above all, all pd-pairs including pd-pair *k* can be transported in $R'_{m}(p_{k}-r_{1}...r_{\text{j}}-d_{k}-r_{\text{j}+1}...r_{n})$, so route *m’* is RSF (**Definition 1** in Section 3.4), namely pd-pair *k* can be inserted into route *m* (**Definition 2** in Section 3.4).

**Case 3:** None of *r_i_* and *r_j_* can be found

The structure of new route *m’* generated by inserting pd-pair *k* into route *m* can be separated into three categories: $R'_{m}(p_{k}-d_{k}-r_{1}...r_{n})$, $R'_{m}(r_{1}...r_{\text{n}}-p_{k}-d_{k})$and $R'_{m}(p_{k}-r_{1}...r_{\text{n}}-d_{k})$.

As for the first two categories, the structure of$path(r_{1}...r_{n})$ has not been changed, because pd-pair *k* can be combined with all pd-pair $r_{\text{s}}-r_{t}$($r_{\text{s}},r_{t}\in\{r_{1},...,r_{n}\}$), and all pd-pairs including pd-pair *k* can be transported in route *m’* obviously, so route *m’* is RSF (**Definition 1** in Section 3.4), namely pd-pair *k* can be inserted into route *m* (**Definition 2** in Section 3.4).

The third category has been proved in **Case 1**.

(2) Necessity

If there is any pd-pair that can’t be combined with pd-pair *k* in route *m*, then pd-pair *k* can’t be inserted into route *m* clearly.

This completes the proof.

1. **Proof of Lemma 1**

**Proof:**

**Lemma 3** can be deduced from **Theorem 1**.

1. **Proof of Lemma 2**

**Proof:**

**Lemma 3** can be deduced from **Theorem 1**.

1. **Proof of Lemma 3**

**Proof:**

Since pd-pair *i* ($i\in P$) can be combined with each other, considering pd-pair *i_1_* ($i_{1}\in P$) as a route *R_1_*, so pd-pair *i_2_* ($i_{2}\in P$) can be inserted into *R_1_* and a new route *R_2_* is acquired (**Theorem 1**), then pd-pair *i_2_* can be inserted into *R_1_*, and so on. Finally *R_m_* is acquired comprised of all pd-pair *i* ($i\in P$).

This completes the proof.
