# Supplementary material for: Multi-start heuristic approaches for one-to-one pickup and delivery problems with shortest-path transport along real-life paths
Source: PLoS One. 2020 Feb 6;15(2):e0227702. doi: 10.1371/journal.pone.0227702 (PMC7004362; doi:10.1371/journal.pone.0227702)
Supplement: S3 Appendix — (DOC) [file pone.0227702.s003.doc]

**S3 Appendix. Instances and relative research.**

Relative data for the instances can be found online at:

https://www.researchgate.net/publication/337151397_Instances_for_the_OPDPSTRP.
