# Supplementary material for: Multi-start heuristic approaches for one-to-one pickup and delivery problems with shortest-path transport along real-life paths
Source: PLoS One. 2020 Feb 6;15(2):e0227702. doi: 10.1371/journal.pone.0227702 (PMC7004362; doi:10.1371/journal.pone.0227702)
Supplement: S4 Appendix — (DOC) [file pone.0227702.s004.doc]

**S4 Appendix. Parameter setting for the VND, the VNS, the MS_VND and the MS_VNS.**

- **parameter setting for the MS_VND**

**Table A**

| **Initial parameter setting for the MS_VND** | | |
| --- | --- | --- |
| **Symbol** | **Definition** | **Value** |
| *p_k_* | Operator choosing probabilities in ***Perturbation*** | 2/7, 2/7, 1/7, 1/7, 1/7 for *Insert*, *Spread*, *Point-delete*, *Rout-delete*, and *Reassign-vehicle* |
| *n* | Size of Multi-Start candidate solution set | 90 |
| *m* | replacing proportion for Multi-Start solution set | 1/8 |
| *T_0_* | Selection controlling value | 50 |
| *constant_T* | Algorithm termination iterations | *constant_T=exp(-20/(2+num_pd-pairs))*500*, *num_pd-pairs* is number of pd-pairs |
| *K* | Iterative numbers controlling value for ***Spread*** | 3 |

Firstly, the parameter setting for the MS_VND is tuned by determining a trade off between solution quality and CPU time after numerous experiments, the initial values are gathered in Table A.

**Table B**

| **Testing instances** | | |
| --- | --- | --- |
| **Instance** | **Cargoes** | **Pd-pairs** |
| 10-10-10-50-1-1 | 188 | 188 |
| 10-10-10-200-3-1 | 45 | 15 |
| 10-10-10-500-1-1 | 20 | 20 |
| 6-8-10-10-10-4 | 258 | 26 |
| 6-8-10-25-3-4 | 90 | 30 |
| 6-8-10-50-10-4 | 30 | 3 |
| 3-4-10-1-10-4 | 132 | 14 |
| 3-4-10-3-3-4 | 43 | 15 |
| 3-4-10-5-1-1 | 25 | 25 |

Secondly, each parameter is tested by fixing the other parameters, and then the final values are determined based on 9 instances (including small size connected graph, medium size connected graph and large size connected graph, as in Table B), each instance is tested 3 times, and average solution and CPU time for each instances are counted, which are proposed as “solution” and “CPU time” in the following sections. Again and again, we get the final parameter setting for the MS_VND, and show them in Table C.

**Table C**

| **Final parameter setting for the MS_VNS** | | |
| --- | --- | --- |
| **Symbol** | **Definition** | **Value** |
| *choosing sequence* | Choosing sequence of operators | *Insert/Spread/Point-delete/Route-delete/Perturbation* |
| *p_k_* | Operator choosing probabilities in ***Perturbation*** | 9/24, 7/24, 1/24, 1/24, 6/24 for *Insert*, *Spread*, *Point-delete*, *Rout-delete*, and *Reassign-vehicle* |
| *n* | Size of Multi-Start candidate solution set | 90 |
| *m* | replacing proportion for Multi-Start solution set | 1/8 |
| *T_0_* | Selection controlling value | 20 |
| *constant_T* | Algorithm termination iterations | *constant_T=exp(-20/(2+num_pd-pairs))*700*, *num_pd-pairs* is number of pd-pairs |
| *K* | Iterative numbers controlling value for ***Spread*** | 3 |

Six testing figures are proposed for each parameter as in Table D.

**Table D**

| **Introduction of figures** | | | | | | |  |
| --- | --- | --- | --- | --- | --- | --- | --- |
| **Figure** | **Definition** | ***choosing sequence*** | ***p_k_*** | ***n*** | ***m*** | ***T0*** | ***constant_T*** |
| Average Improvement Efficiency of all instances | average improvement times/average transformation times of all instances (%) | √ |  |  |  |  |  |
| Average Improvement/Time Efficiency of all instances | average improvement times/average CPU time of all instances(times/second) | √ |  |  |  |  |  |
| Normalized Solutions | solution of each instance /max solution of all instance according to different parameter values (0~1) |  | √ | √ | √ | √ | √ |
| Average Solution of all instances | average solution of all instances according to different parameter values ( value) |  | √ | √ | √ | √ | √ |
| Average Time Efficiency of all instances | average solution/average CPU time of all instances according to different parameter values ( value/second) |  | √ | √ | √ | √ | √ |
| Average CPU Time of all instances | average CPU time of all instances according to different parameter values (second) |  | √ | √ | √ | √ | √ |

The testing results are shown as follows.

(1) choosing sequence of operators and testing result of *p_k_*

Fig A shows the performance of four operators which are chosen separately in the MS_VND (operator 5 is removed).


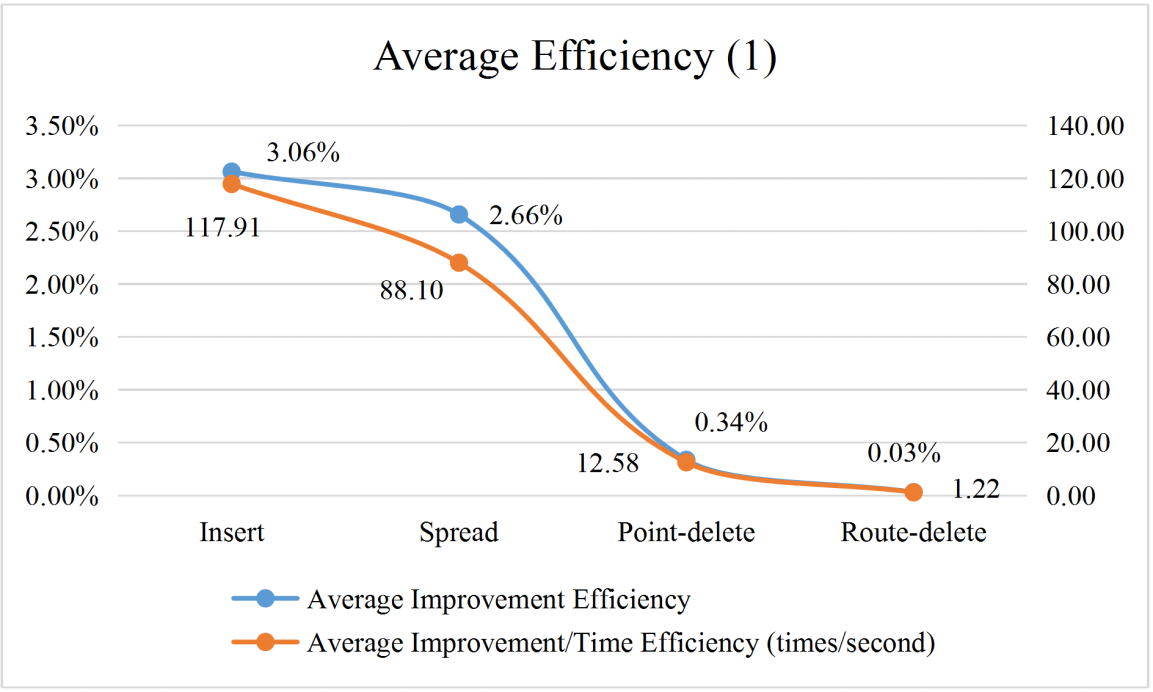


**Fig A Average Efficiency (1)**

According to Fig A, the choosing sequence of the operators is determined as *Insert/Spread/ Point-delete/Route-delete* for the VND, the VNS, the MS_VND and the MS_VNS, and operator choosing ratios between then are determined as 9:7:1:1 for the Perturbation.


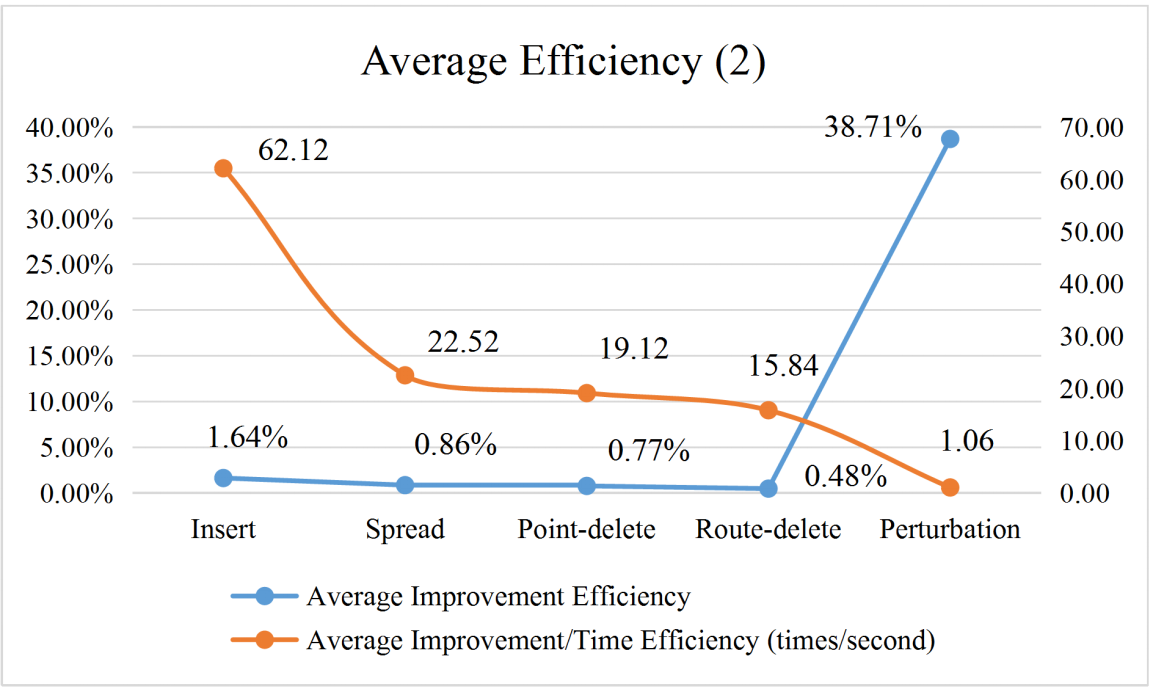


**Fig B Average Efficiency (2)**

Fig B shows the performance of five operators in the MS_VND algorithm with the empirical values in the article, result shows that Reassign-vehicle will take a lot of CPU time. So the choosing sequence of the operators is determined as *Insert/Spread/Point-delete/Route-delete/Perturbation* for the VND, the VNS, the MS_VND and the MS_VNS choosing probability of Reassign-vehicle will be studied individually.

In order to keep the ratio 9:7:1:1 for *Insert*, *Spread*, *Point-delete* and *Route-delete*, choosing probability of *Reassign-vehicle* is set as 0 (0/18), 1/19 (1/1+18), ..., 9/27(9/9+18) in the MS_VND. The MS_VND with different *p_k_*=0, 1/19, 2/20, 3/21, 4/22, 5/23, 6/24, 7/25, 8/26, 9/27 and 1 are tested by 9 instances. Each instance is tested three time and the other parameters are set as in Table C.


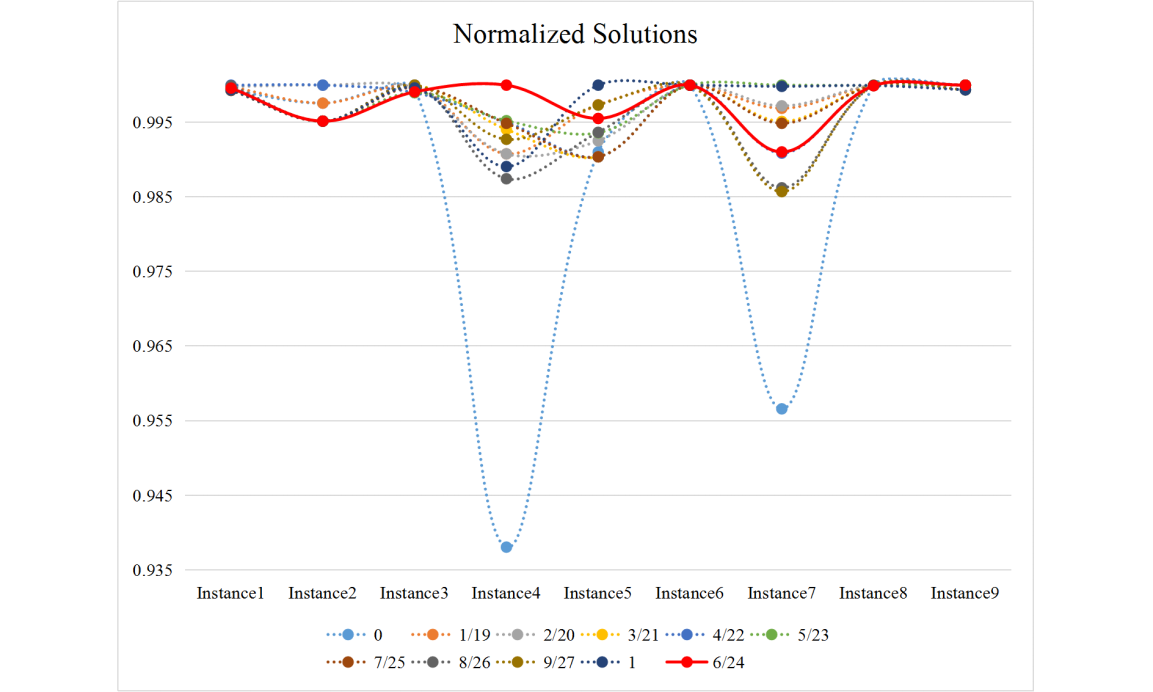


**Fig C Normalized Solutions for *p_k_* of MS_VND**


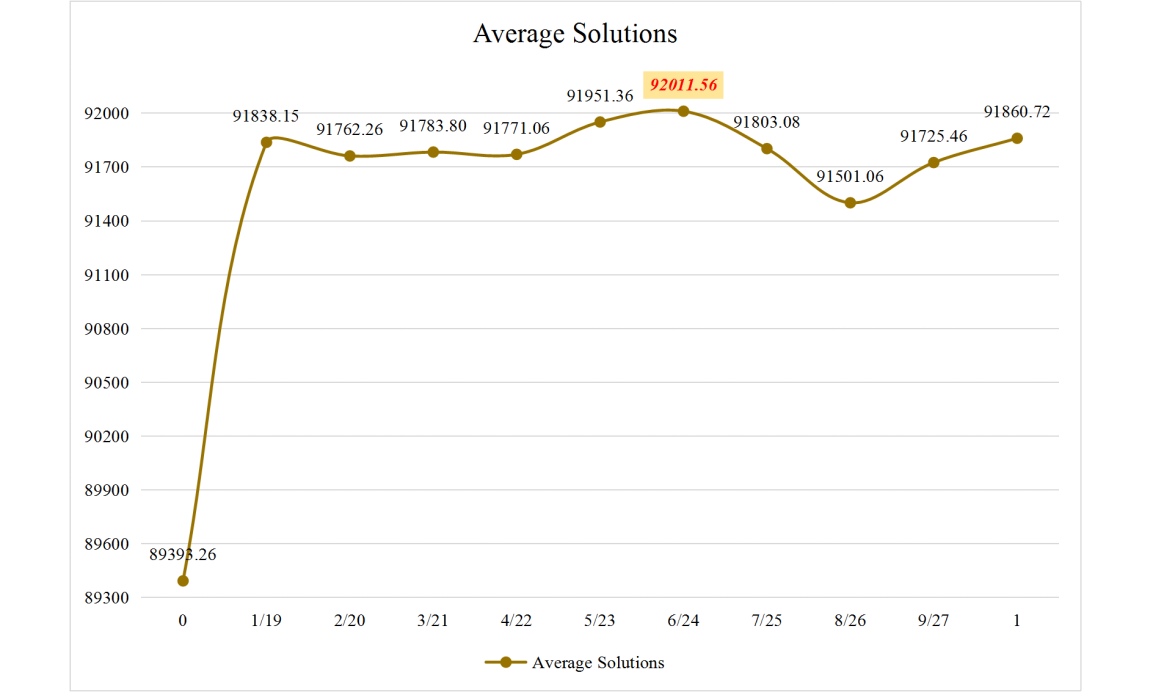


**Fig D Average Solutions for *p_k_* of MS_VND**


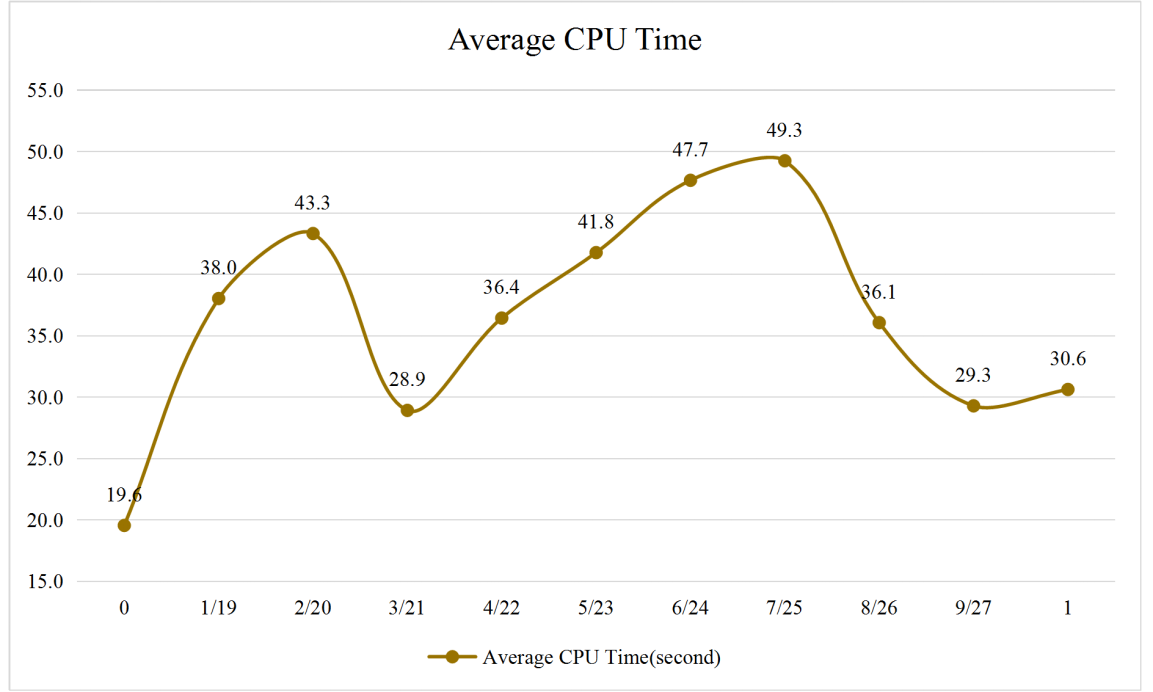


**Fig E Average CPU Time for *p_k_* of MS_VND**


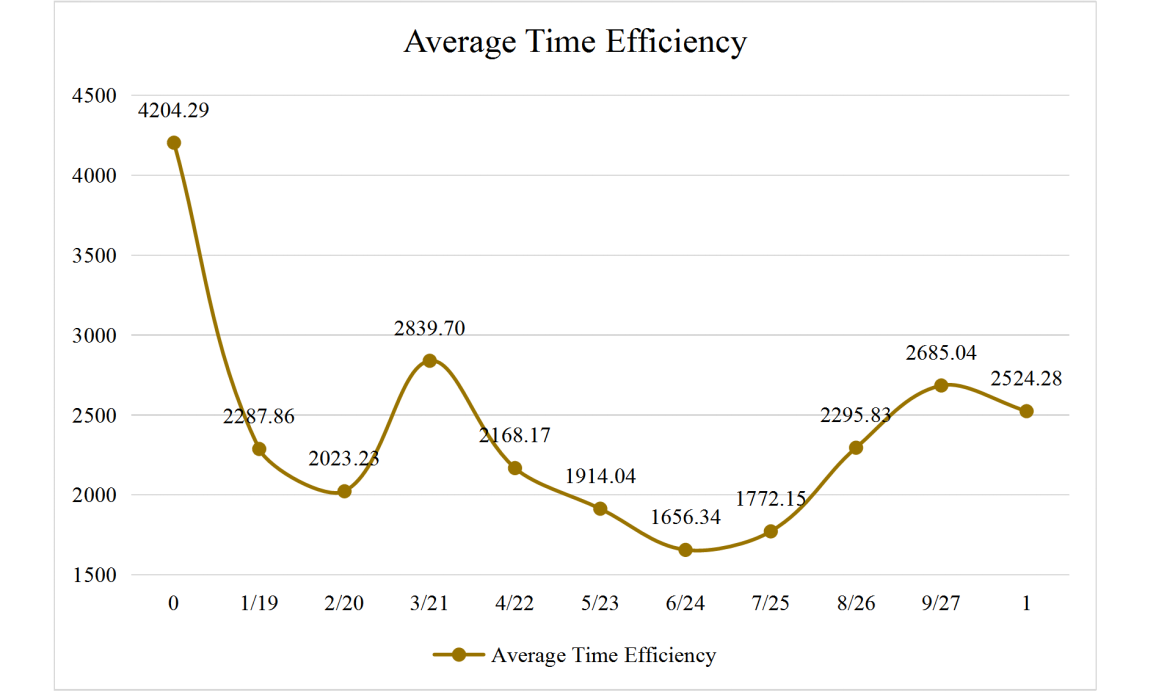


**Fig F Average Time Efficiency for *p_k_* of MS_VND**

According the above 4 figures, the MS_VND with *p*=6/24 and other fixed parameters can obtain the best average solution value without costing too much CPU time, when the other parameters are fixed as in Table C. **So *p_k_*=6/24 is chosen for the MS_VND in our paper.**

(2) testing result of *n*

The MS_VND with different *n*=1, 10, 30, 60, 90, 120, 150, 180, 210 are tested by 9 instances. Each instance is tested three time and the other parameters are set as in Table C.


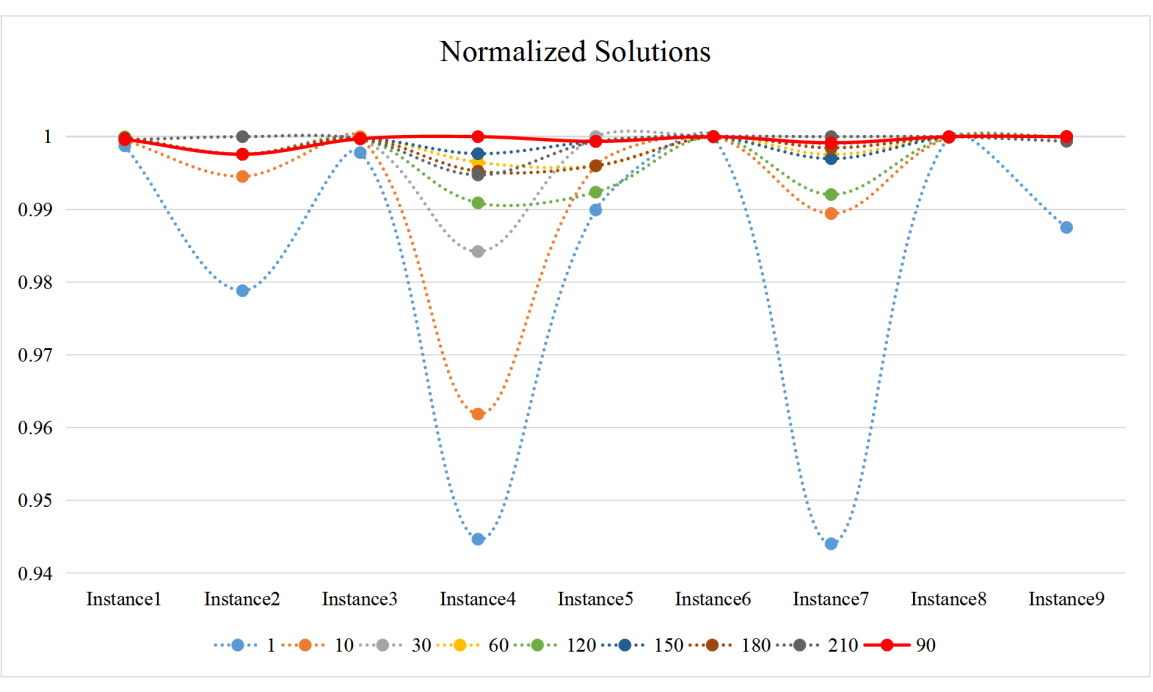


**Fig G Normalized Solutions for *n* of MS_VND**


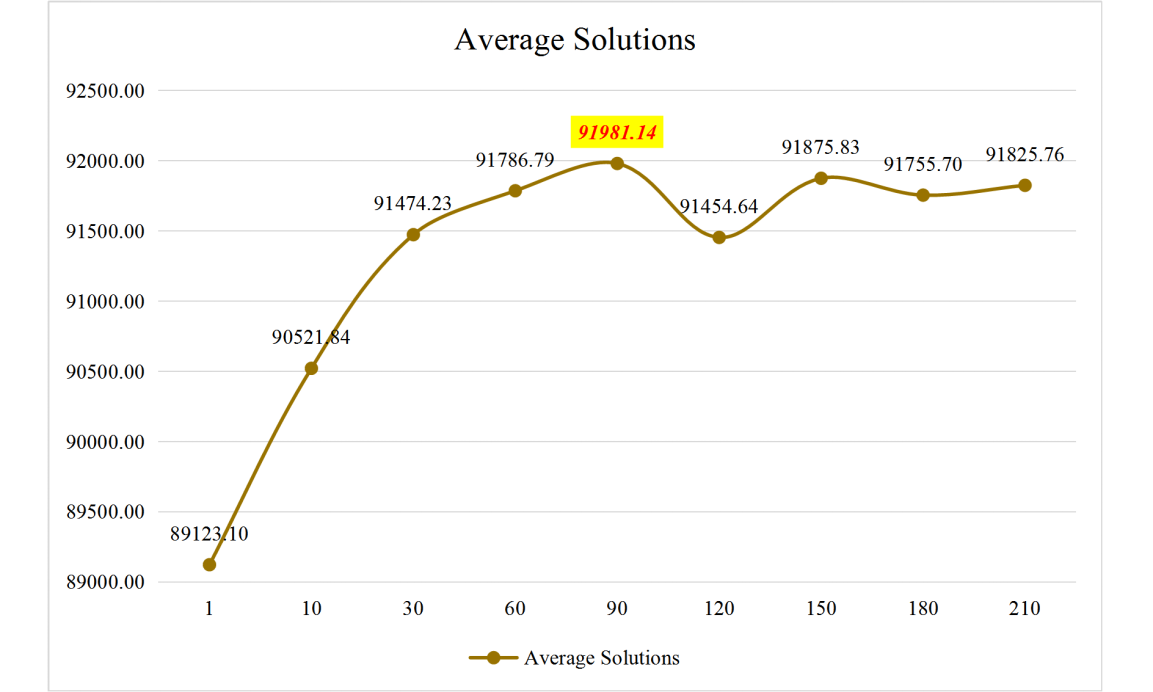


**Fig H Average Solutions for *n* of MS_VND**


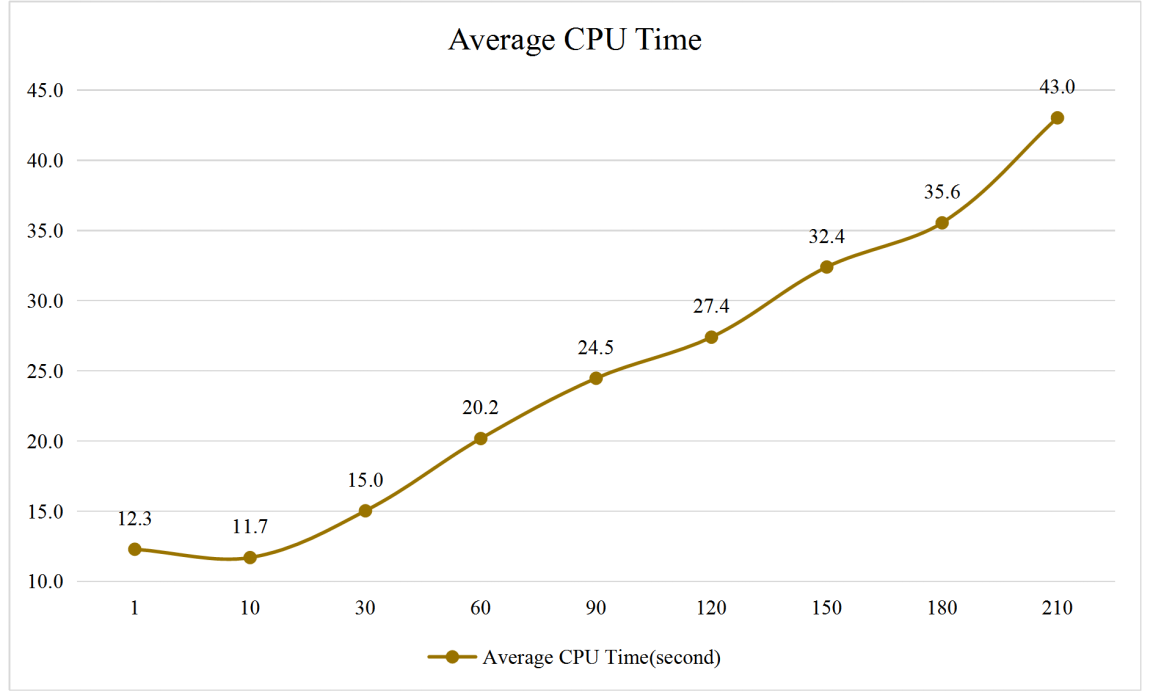


**Fig I Average CPU Time for *n* of MS_VND**


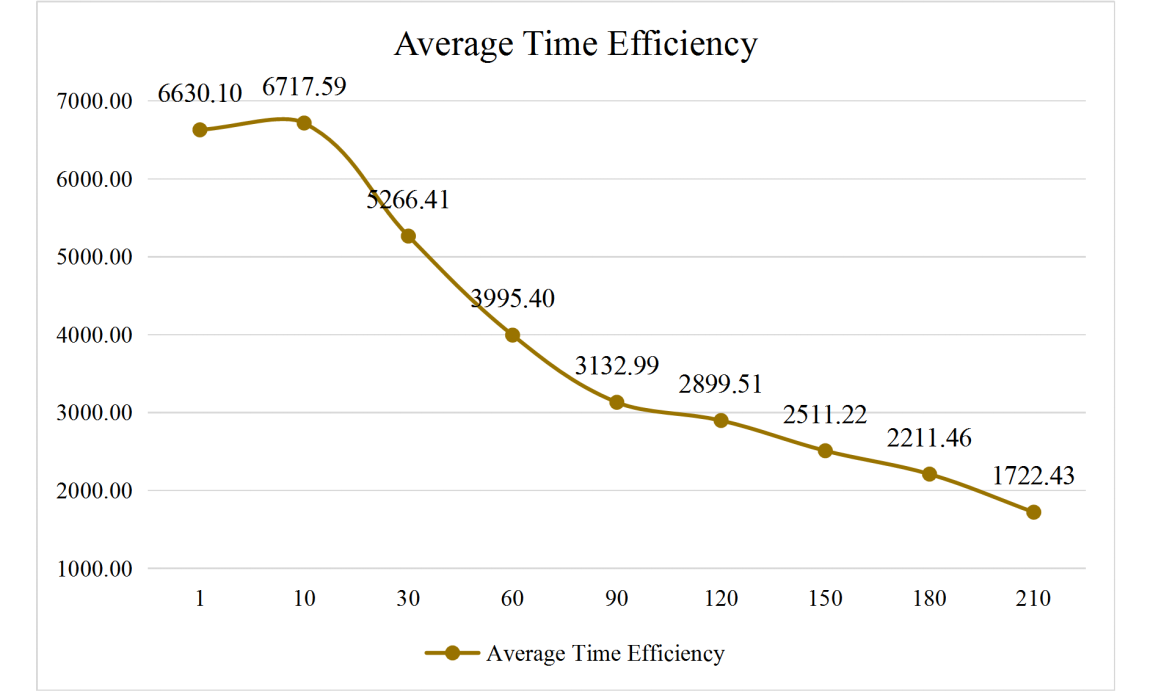


**Fig J Average Time Efficiency for *n* of MS_VND**

According the above 4 figures, the MS_VND with *n*=90 and other fixed parameters can obtain the best average solution value without costing too much CPU time, when the other parameters are fixed as in Table C. **So *n*=90 is chosen for the MS_VND in our paper.**

(3) testing result of *m*

The MS_VND with different *m*=0, 1/64, 1/32, 1/16, 1/8, 1/4, 1/3, 1/2, 1 are tested by 9 instances. Each instance is tested three time and the other parameters are set as in Table C.


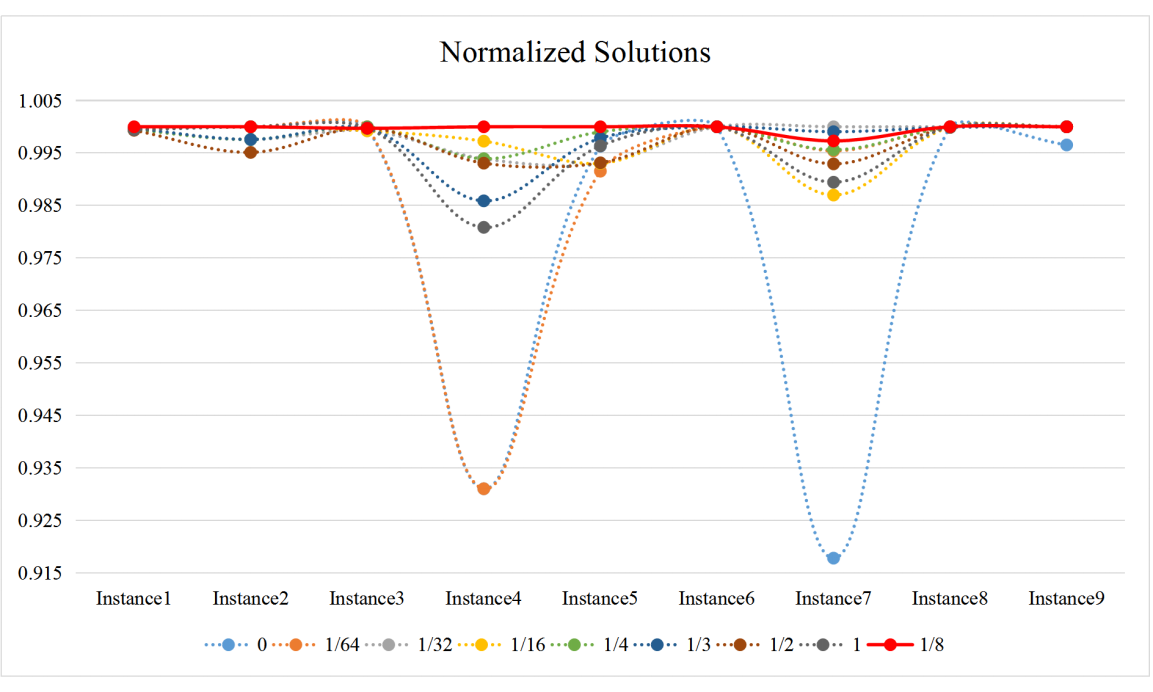


**Fig K Normalized Solutions for *m* of MS_VND**


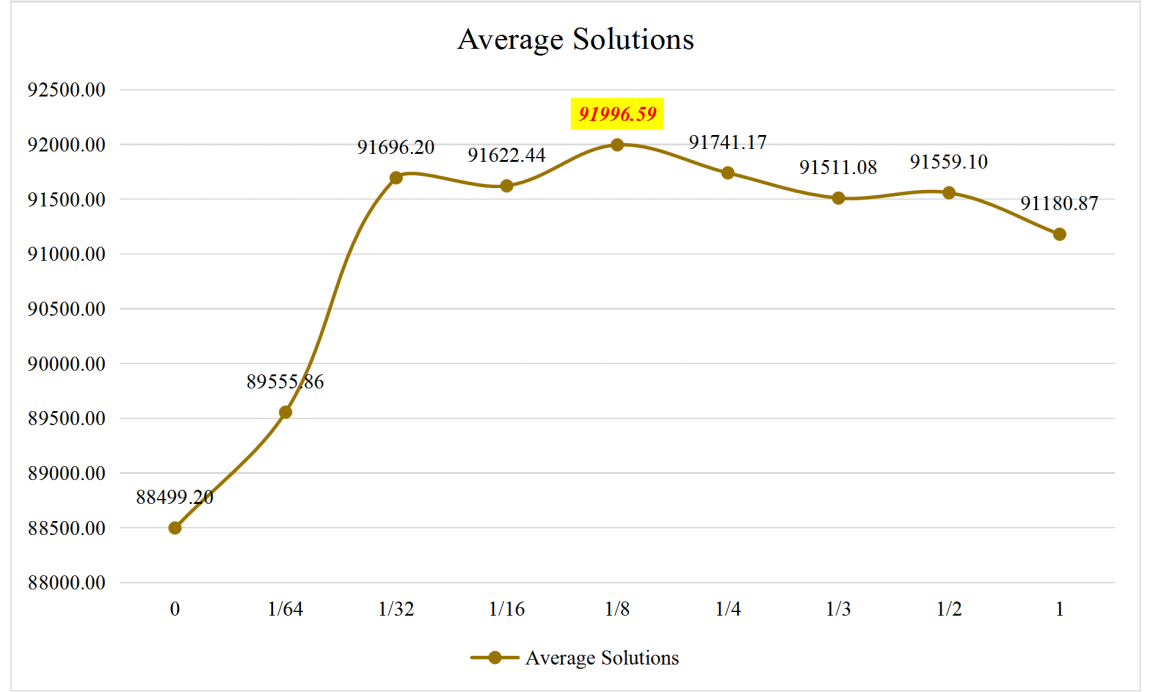


**Fig L Average Solutions for *m* of MS_VND**


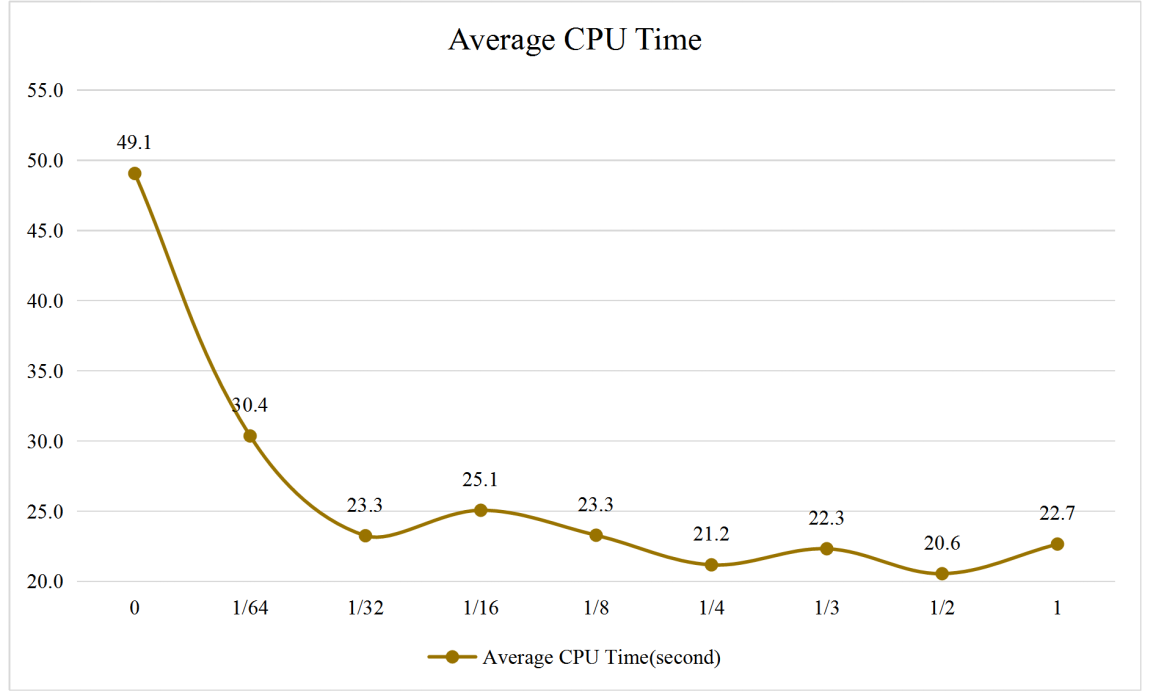


**Fig M Average CPU Time for *m* of MS_VND**


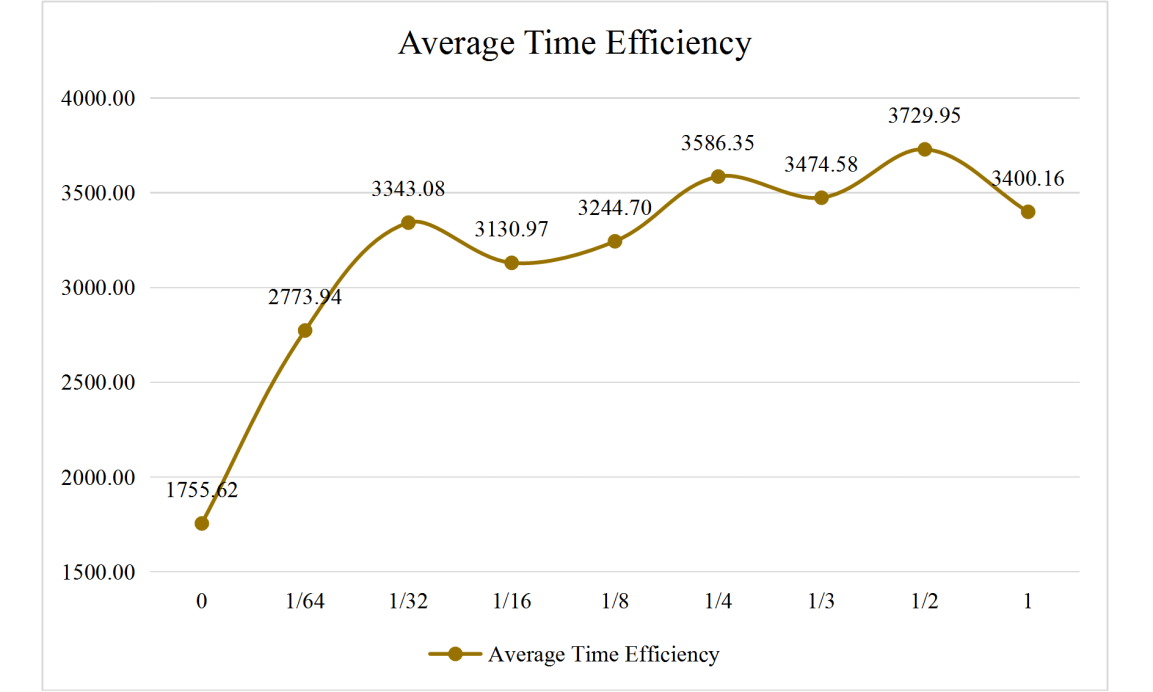


**Fig N Average Time Efficiency for *m* of MS_VND**

According the above 4 figures, the MS_VND with *m*=1/8 and other fixed parameters can obtain the best average solution value without costing too much CPU time, when the other parameters are fixed as in Table C. **So *m*=1/8 is chosen for the MS_VND in our paper.**

(4) testing result of *T_0_*

The MS_VND with different *T_0_*=1, 3, 5, 7, 10, 15, 20, 30, 50, 100 and 150 are tested by 9 instances. Each instance is tested three time and the other parameters are set as in Table C.


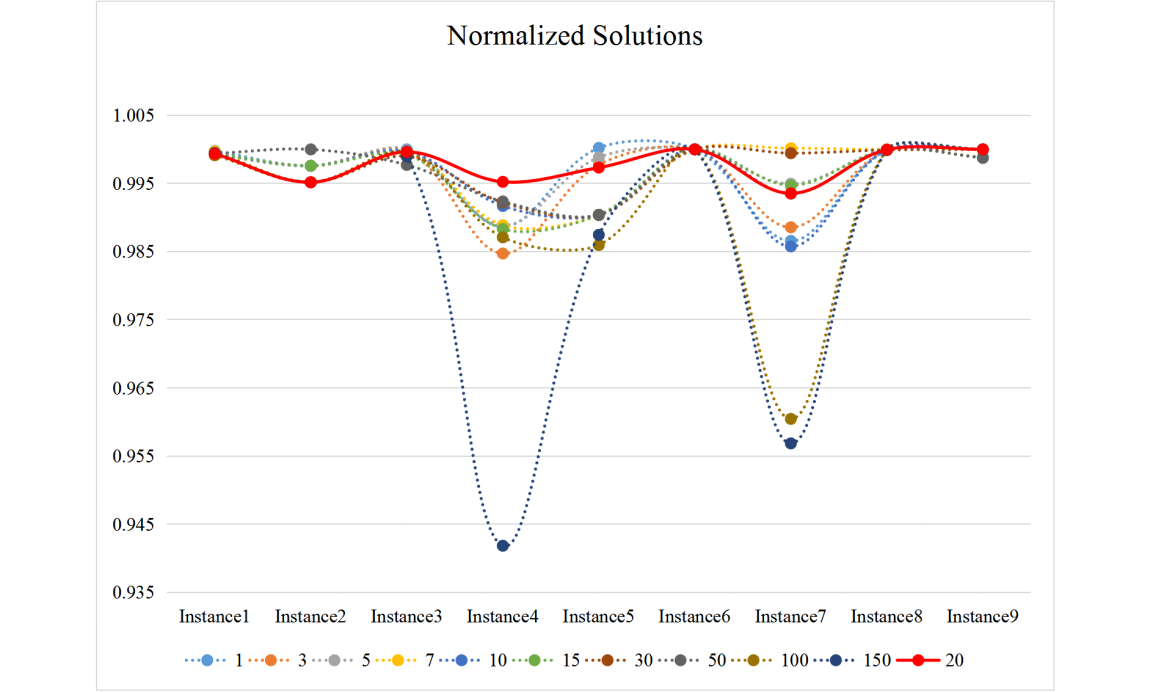


**Fig O Normalized Solutions for *T_0_* of MS_VND**


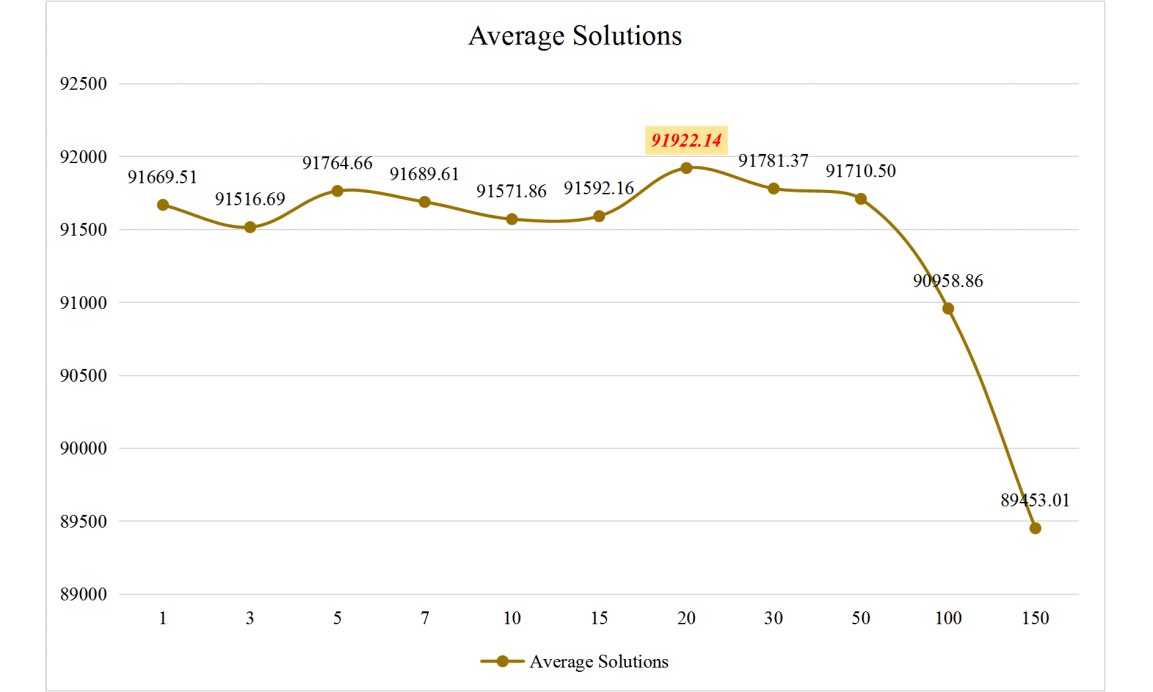


**Fig P Average Solutions for *T_0_* of MS_VND**


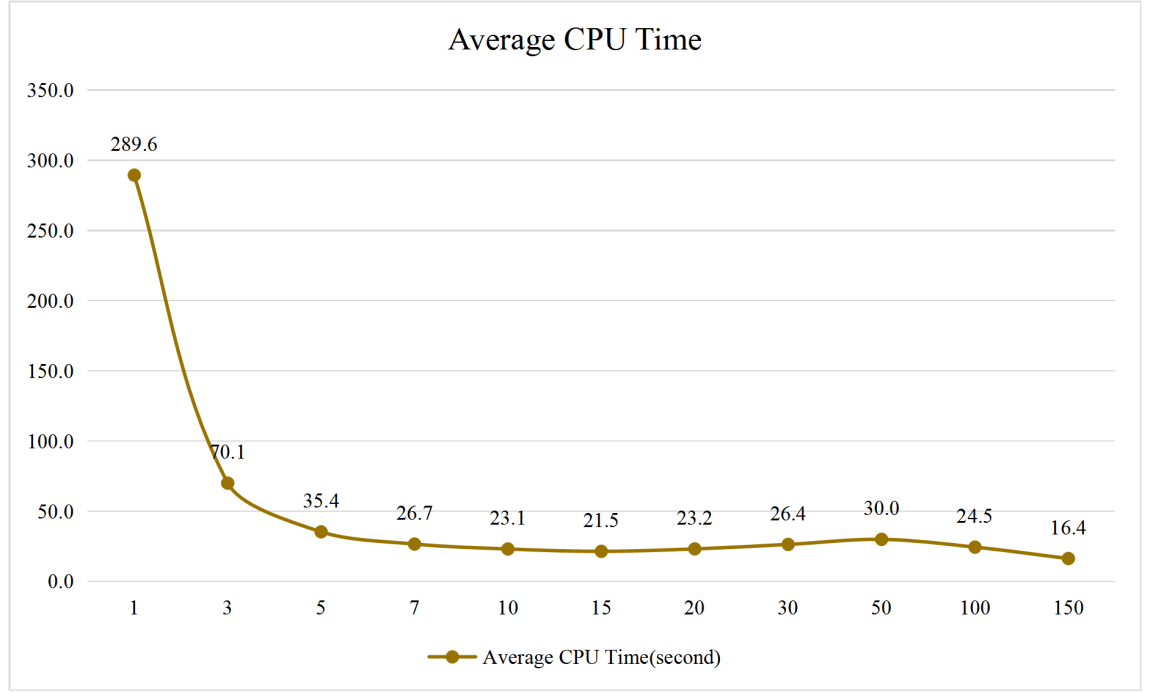


**Fig Q Average CPU Time for *T_0_* of MS_VND**


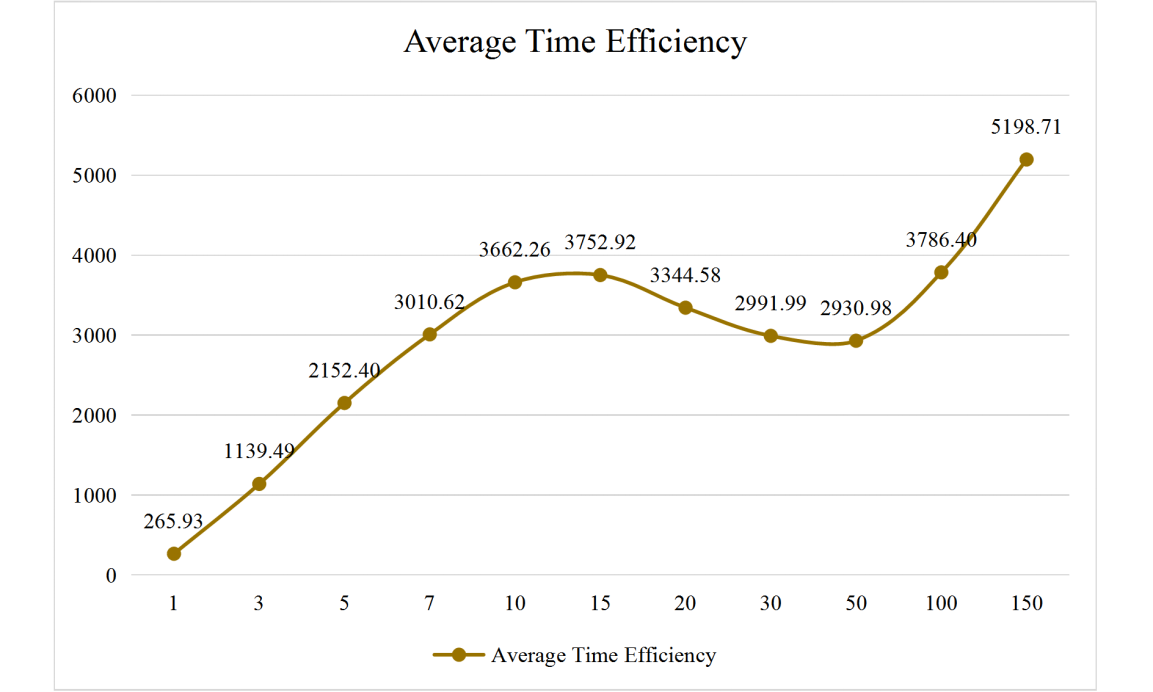


**Fig R Average Time Efficiency for *T_0_* of MS_VND**

According the above 4 figures, the MS_VND with *T_0_*=20 and other fixed parameters can obtain the best average solution value without costing too much CPU time, when the other parameters are fixed as in Table C. **So *T0*=20 is chosen for the MS_VND in our paper.**

(5) testing result of *constant_T*

The MS_VND with different *constant_T*=100, 200, 300, 400, 500, 600, 700, 800, 900, 1000 and 1100 are tested by 9 instances. Each instance is tested three time and the other parameters are set as in Table C.


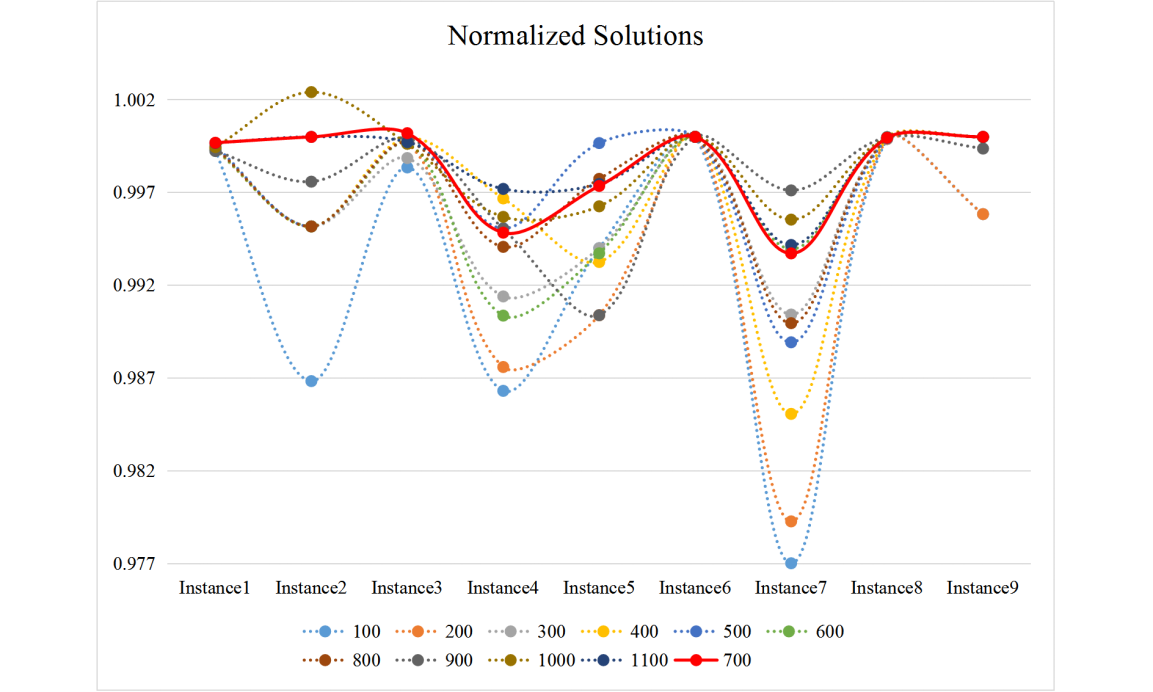


**Fig S Normalized Solutions for *constant_T* of MS_VND**


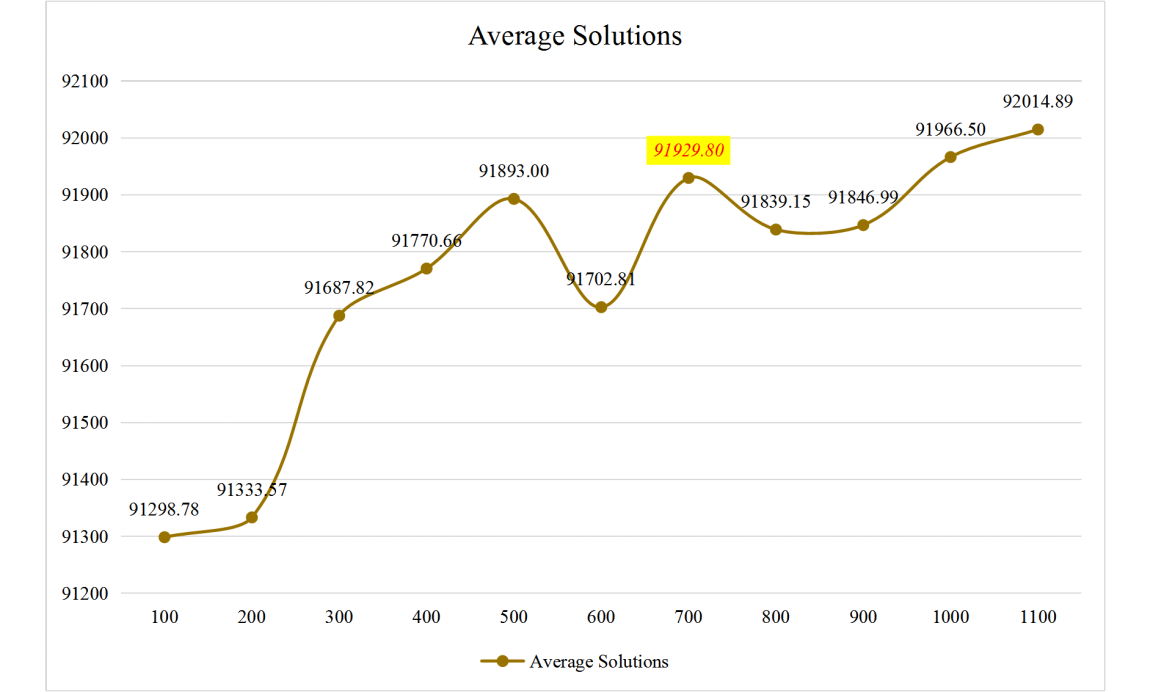


**Fig T Average Solutions for *constant_T* of MS_VND**


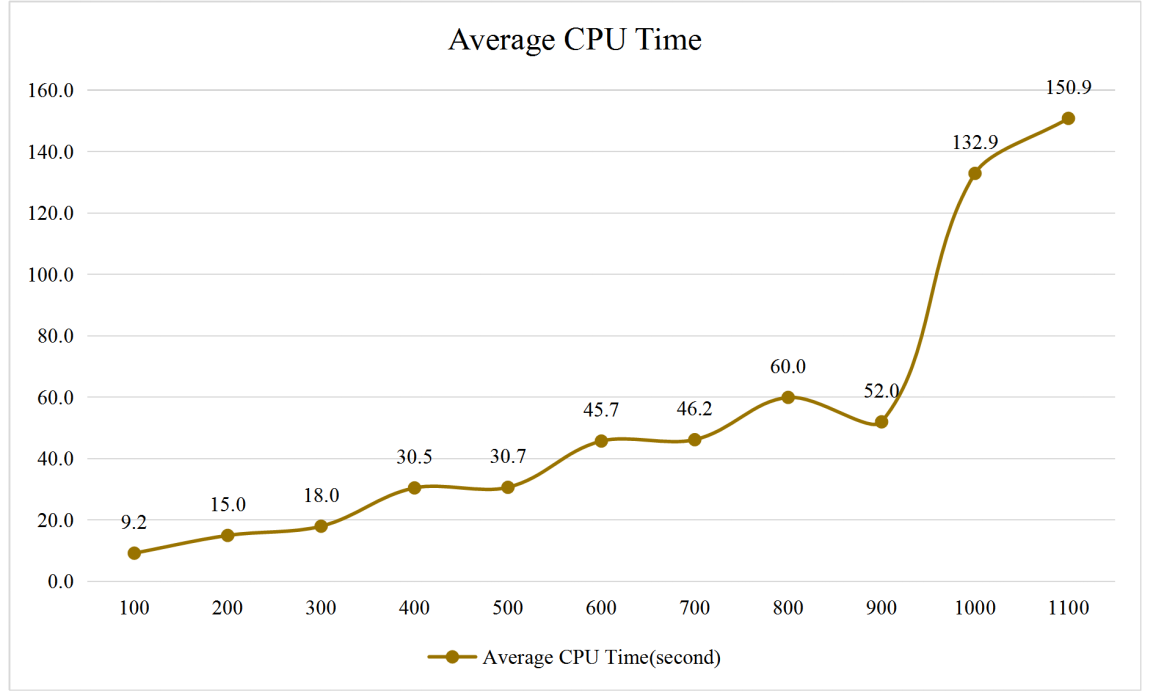


**Fig U Average CPU Time for *constant_T* of MS_VND**


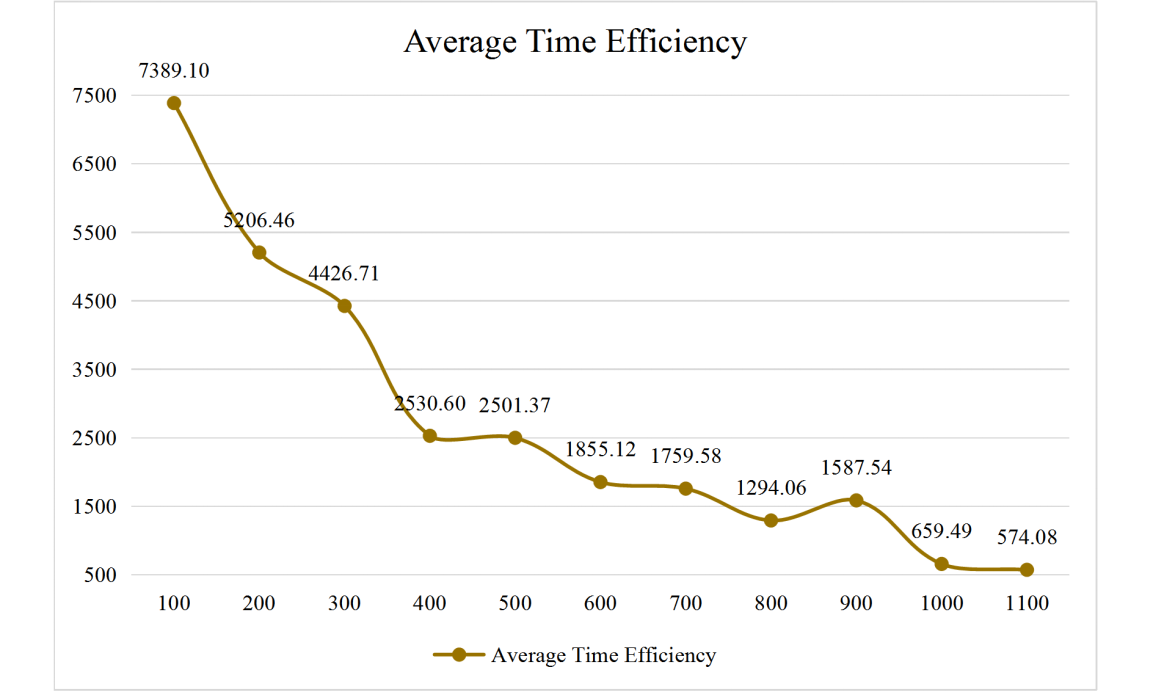


**Fig V Average Time Efficiency for *constant_T* of MS_VND**

According the above 4 figures, the MS_VND with *constant_T*=700 and other fixed parameters can obtain the best average solution value without costing too much CPU time, when the other parameters are fixed as in Table C. **So *constant_T*=700 is chosen for the MS_VND in our paper.**

1. testing result of *K*

*K* is proposed to avoid too many cycles in operator *Spread*. It’s found that “*Spread*” are always executed no more than 3 times in operator *Spread*, and there is not much difference between different *K* when *K*≥3. **So *K*=3 is chosen for the algorithms in our paper.**

In summary, the parameter setting in the MS_VND is determined as in Table C.

- **parameter setting for the VND**

According to Fig A and Fig B, the choosing sequence of the operators is determined as *Insert/Spread/ Point-delete/Route-delete/Perturbation* for the VND, the VNS, the MS_VND and the MS_VNS, and operator choosing ratios between then are determined as 9:7:1:1 for the Perturbation.

For the better comparison, *p_k_*, *constant_T*, *K* for the VND are set as the same as in the MS_VND, the final values of the parameters for the VND are shown in Table E.

**Table E**

| **Parameter setting for the VND** | | |
| --- | --- | --- |
| **Symbol** | **Definition** | **Value** |
| *choosing sequence* | Choosing sequence of operators | *Insert/Spread/Point-delete/Route-delete/Perturbation* |
| *p_k_* | Operator choosing probabilities in ***Perturbation*** | 9/24, 7/24, 1/24, 1/24, 6/24 for *Insert*, *Spread*, *Point-delete*, *Rout-delete*, and *Reassign-vehicle* |
| *T_0_* | Selection controlling value | 20 |
| *constant_T* | Algorithm termination iterations | *constant_T=exp(-20/(2+num_pd-pairs))*700*, *num_pd-pairs* is number of pd-pairs |
| *K* | Iterative numbers controlling value for ***Spread*** | 3 |

- **parameter setting for the VNS**

According to Fig A and Fig B, the choosing sequence of the operators is determined as *Insert/Spread/ Point-delete/Route-delete/Perturbation* for the VND, the VNS, the MS_VND and the MS_VNS, and operator choosing ratios between then are determined as 9:7:1:1 for the Perturbation.

For the better comparison, *p_k_*, *constant_T*, *K* for the VNS are set as the same as in the MS_VND, the final values of the parameters for the VNS are shown in Table F.

**Table F**

| **Parameter setting for the VNS** | | |
| --- | --- | --- |
| **Symbol** | **Definition** | **Value** |
| *choosing sequence* | Choosing sequence of operators | *Insert/Spread/Point-delete/Route-delete/Perturbation* |
| *p_k_* | Operator choosing probabilities in ***Perturbation*** | 9/24, 7/24, 1/24, 1/24, 6/24 for *Insert*, *Spread*, *Point-delete*, *Rout-delete*, and *Reassign-vehicle* |
| *constant_T* | Algorithm termination iterations | *constant_T=exp(-20/(2+num_pd-pairs))*700*, *num_pd-pairs* is number of pd-pairs |
| *K* | Iterative numbers controlling value for ***Spread*** | 3 |

- **parameter setting for the MS_VNS**

According to Fig A and Fig B, the choosing sequence of the operators is determined as *Insert/Spread/ Point-delete/Route-delete/Perturbation* for the VND, the VNS, the MS_VND and the MS_VNS, and operator choosing ratios between then are determined as 9:7:1:1 for the Perturbation.

For the better comparison, *p_k_*, *K* for the MS_VNS are set as the same as in the MS_VND. *n* for the MS_VNS will be studied individually, because it is found that *n* has great influences on the solution quality and CPU time of the MS_VNS.


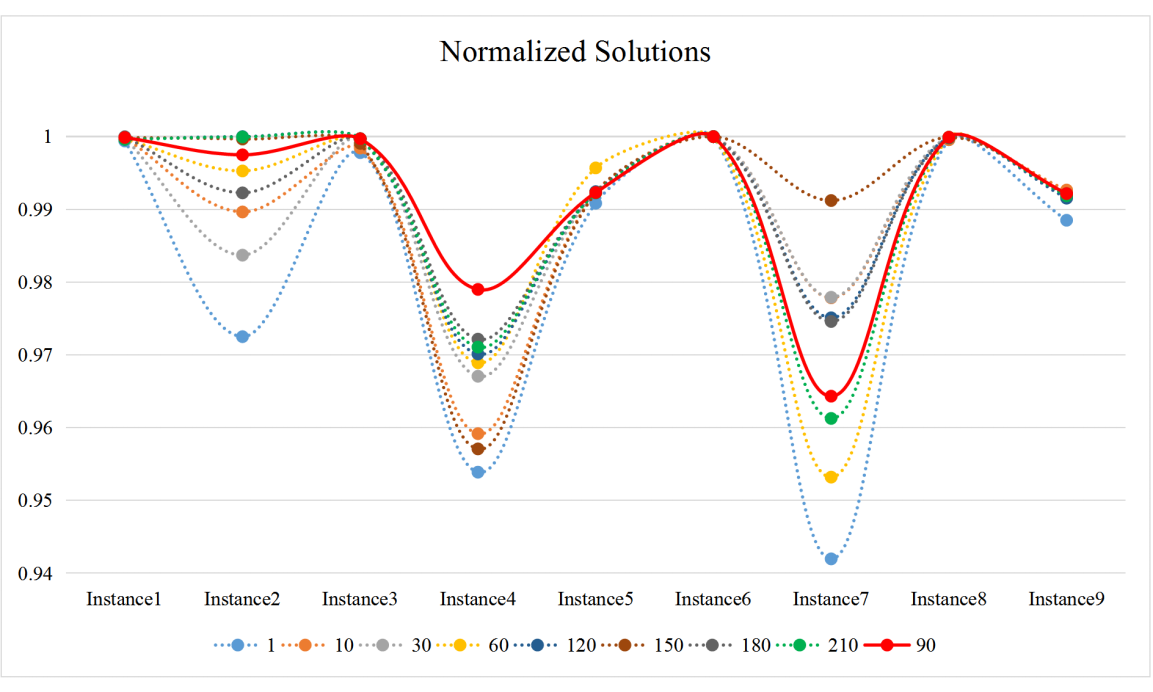


**Fig W Normalized Solutions for *n* of MS_VNS**


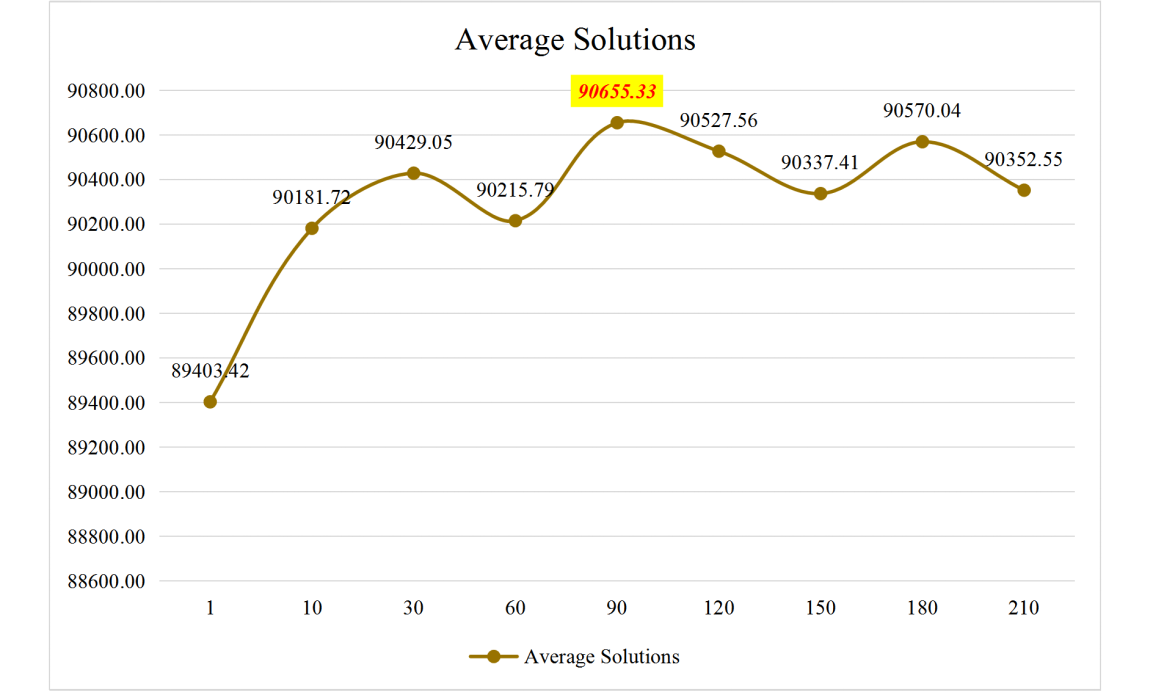


**Fig X Average Solutions for *n* of MS_VNS**


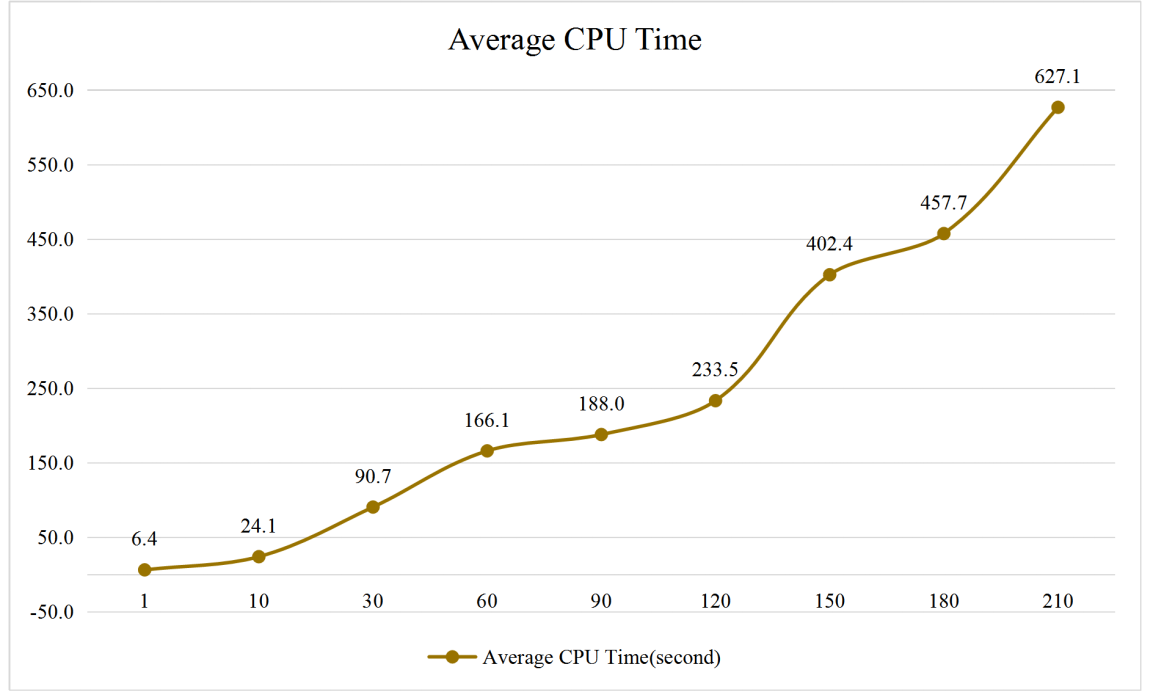


**Fig Y Average CPU Time for *n* of MS_VNS**


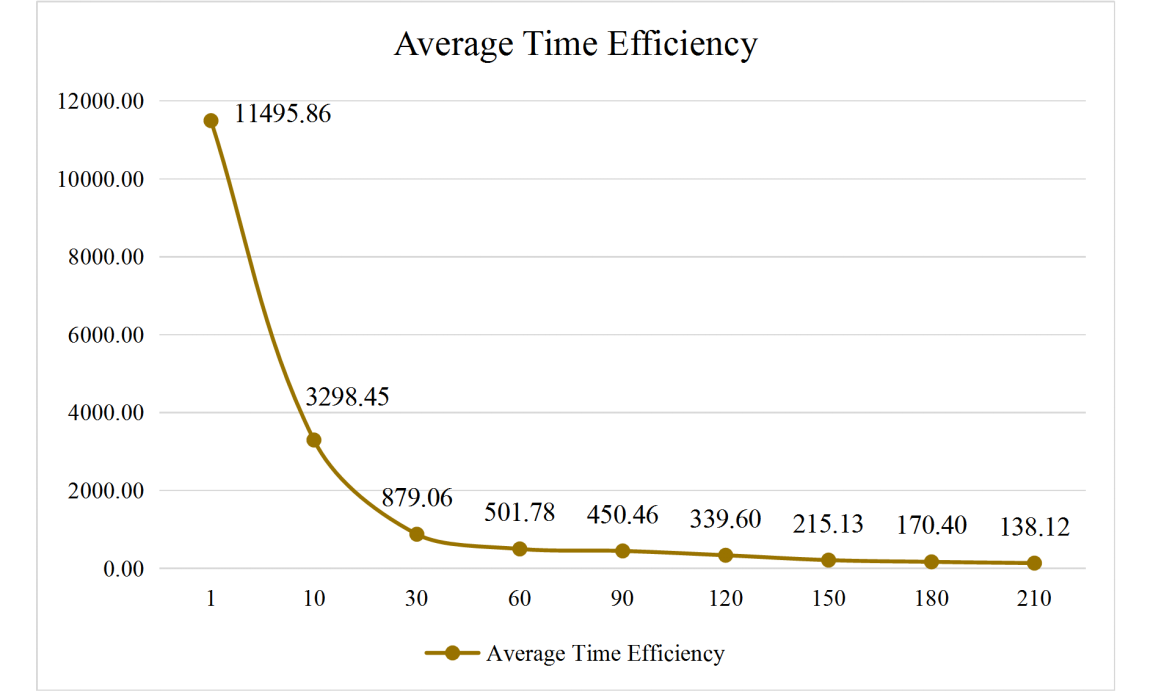


**Fig Z Average Time Efficiency for *n* of MS_VNS**

According the above 4 figures, the MS_VNS with *n*=90 and other fixed parameters can obtain the best average solution value without costing too much CPU time, when the other parameters are fixed as in Table C. **So *n*=90 is chosen for the MS_VNS in our paper.**

The final parameter setting for the MS_VNS are shown in Table G.

**Table G**

| **Parameter setting for the MS_VNS** | | |
| --- | --- | --- |
| **Symbol** | **Definition** | **Value** |
| *choosing sequence* | Choosing sequence of operators | *Insert/Spread/Point-delete/Route-delete/Perturbation* |
| *p_k_* | Operator choosing probabilities in ***Perturbation*** | 9/24, 7/24, 1/24, 1/24, 6/24 for *Insert*, *Spread*, *Point-delete*, *Rout-delete*, and *Reassign-vehicle* |
| *constant_T* | Algorithm termination iterations | *constant_T=exp(-20/(2+num_pd-pairs))*700*, *num_pd-pairs* is number of pd-pairs |
| *K* | Iterative numbers controlling value for ***Spread*** | 3 |
